# Resveratrol reduces RVLM neuron activity *via* activating the AMPK/Sirt3 pathway in stress-induced hypertension

**DOI:** 10.1016/j.jbc.2025.108394

**Published:** 2025-03-10

**Authors:** Lin-Ping Wang, Tian-Feng Liu, Teng-Teng Dai, Xin Deng, Lei Tong, Qiang-Cheng Zeng, Qing He, Zhang-Yan Ren, Hai-Li Zhang, Hai-Sheng Liu, Yan-Fang Li, Wen-Zhi Li, Shuai Zhang, Dong-Shu Du

**Affiliations:** 1 School of Environmental and Chemical Engineering, Shanghai University, Shanghai, China; 2 School of Life Sciences, Shanghai University, Shanghai, China; 3 Shanghai Institute of Cardiovascular Diseases, Zhongshan Hospital, Institute of Biomedical Science, Fudan University, Shanghai, China; 4 College of Life Sciences, Dezhou University, Dezhou, Shandong, China; 5 College of Agriculture and Bioengineering, Heze University, Heze, Shandong, China; 6 Department of Preventive Medicine, Heze Medical College, Heze, Shandong, China; 7 Department of Urology, School of Medicine, Shanghai General Hospital, Shanghai Jiao Tong University, Shanghai, China; 8 International Cooperation Laboratory of Molecular Medicine, Academy of Chinese Medical Sciences, Zhejiang Chinese Medical University, Hangzhou, Zhejiang, China

**Keywords:** mitochondrial oxidative stress, reactive oxygen species, RVLM, apoptosis, ferroptosis

## Abstract

Neuronal hyperexcitability in the rostral ventrolateral medulla (RVLM), driven by oxidative stress, plays a crucial role in stress-induced hypertension (SIH). While resveratrol (RSV) is known for its antioxidant properties, its effects on RVLM neurons in SIH remain unclear. We investigated this using an SIH rat model exposed to electric foot shocks and noise stimulation for 15 days. Analysis of RVLM tissue revealed increased mitochondrial damage, oxidative stress, apoptosis, and dysregulated ferroptosis in SIH rats. RSV microinjection into the RVLM reduced blood pressure, sympathetic vascular tone, and neuronal excitability. Both *in vivo* and *in vitro* studies showed that RSV treatment alleviated mitochondrial oxidative stress, apoptosis, and ferroptosis through AMPK activation and subsequent Sirt3 upregulation. These therapeutic effects were blocked by either AMPK inhibition or Sirt3 knockdown. Our findings demonstrate that RSV attenuates SIH by activating the AMPK/Sirt3 pathway, thereby reducing RVLM oxidative stress and cell death.

Hypertension, a highly prevalent disease, poses a significant risk for stroke, heart dysfunction, myocardial infarction, and renal damage (1). The onset of hypertension is influenced by multiple factors, including high-salt diet, age, and genetic predisposition (2). Extensive literature has established that prolonged psychological and mental stress can elevate blood pressure (BP), commonly termed SIH (3, 4, 5). Enhanced sympathetic nerve activity is well-documented in the pathogenesis of hypertension (6), and sympathoexcitation induced by stressors is considered the primary driver of SIH (7, 8, 9). The hypothalamic paraventricular nucleus, rostral ventrolateral medulla (RVLM), and nucleus tractus solitarius are key cerebral nuclei responsible for regulating sympathetic tone and pressor response (6, 10). The RVLM, housing presympathetic neurons, plays a predominant role in the sympathetic regulation of BP (11). Neuronal overactivity within the RVLM leads to enhanced sympathetic outflow and elevated BP (12). Conversely, suppressing RVLM neuronal excitability can alleviate sympathetic overdrive and impede hypertension development. Therefore, elucidating the precise mechanisms underlying neuronal hyperactivation in the RVLM and developing effective treatments are crucial for managing SIH.

Oxidative stress arises when the equilibrium between reactive oxygen species (ROS) generation and antioxidant defense system is disrupted (13). Mitochondria, central regulators of energy metabolism and redox homeostasis, are both the primary intracellular origin and target of ROS (14). Cumulative evidence indicates that mitochondrial dysfunction in neurons leads to ROS overproduction, subsequently contributing to oxidative stress and playing a role in various grave diseases, including Parkinson’s disease, epilepsy, and neurogenic hypertension (15, 16, 17). Our recent works have confirmed that damaged mitochondria within RVLM neurons induce oxidative stress, increase neuronal excitability and sympathetic tone, ultimately promoting SIH progression (18, 19). Elevated oxidative stress is associated with programmed cell death events, including apoptosis and ferroptosis (20). Ferroptosis distinguishes itself from classical cell death mechanisms by its reliance on excessive intracellular iron, ROS accumulation, and lipid peroxidation (21, 22). Impaired endogenous antioxidant defenses also contribute to ferroptosis. Studies have revealed the regulatory role of ferroptosis in the development of stroke, kidney failure, and neurodegenerative diseases (23, 24, 25). Ferroptosis inhibition has been shown to attenuate hippocampal neuronal excitability in pentylenetetrazole-kindled epilepsy (26), but its involvement in SIH progression remains unclear.

RSV, a natural polyphenol compound found in plants like blueberries, grapes, and mulberries (27), exhibits diverse pharmacological properties, including antioxidant and neuroprotective characteristics, contributing to well-documented health benefits (28). Studies have confirmed RSV’s ability to mitigate myocardial ischemic injury by enhancing antioxidant defenses (29) and prevent oxidative stress–mediated podocyte apoptosis, thereby reducing the damage to kidney tissue in diabetes (30). RSV has also been investigated as a potential therapy for Parkinson’s disease and other neurological disorders (31, 32) and possesses the capacity to eliminate ferroptosis due to its effective ROS scavenging ability (33). However, the impact of RSV on mitochondrial oxidative stress, apoptosis, and ferroptosis in RVLM neurons during SIH remains elusive. Given our previous study highlighting the vital role of the AMPK-dependent Sirt3 activation in hindering SIH development (18), exploring the potential involvement of the AMPK/Sirt3 pathway in this process is warranted.

Herein, we revealed that RSV abolished mitochondria-associated oxidative stress and subsequent apoptosis and ferroptosis by enhancing the AMPK/Sirt3 pathway activity. This, in turn, ameliorated RVLM neuronal excitability, sympathetic tone, and BP. This study provided a novel therapeutic avenue for SIH treatment, with RSV as the core therapeutic agent.

## Results

### Chronic stress induces mitochondrial dysfunction and cell death in the RVLM

**Figure 1:**
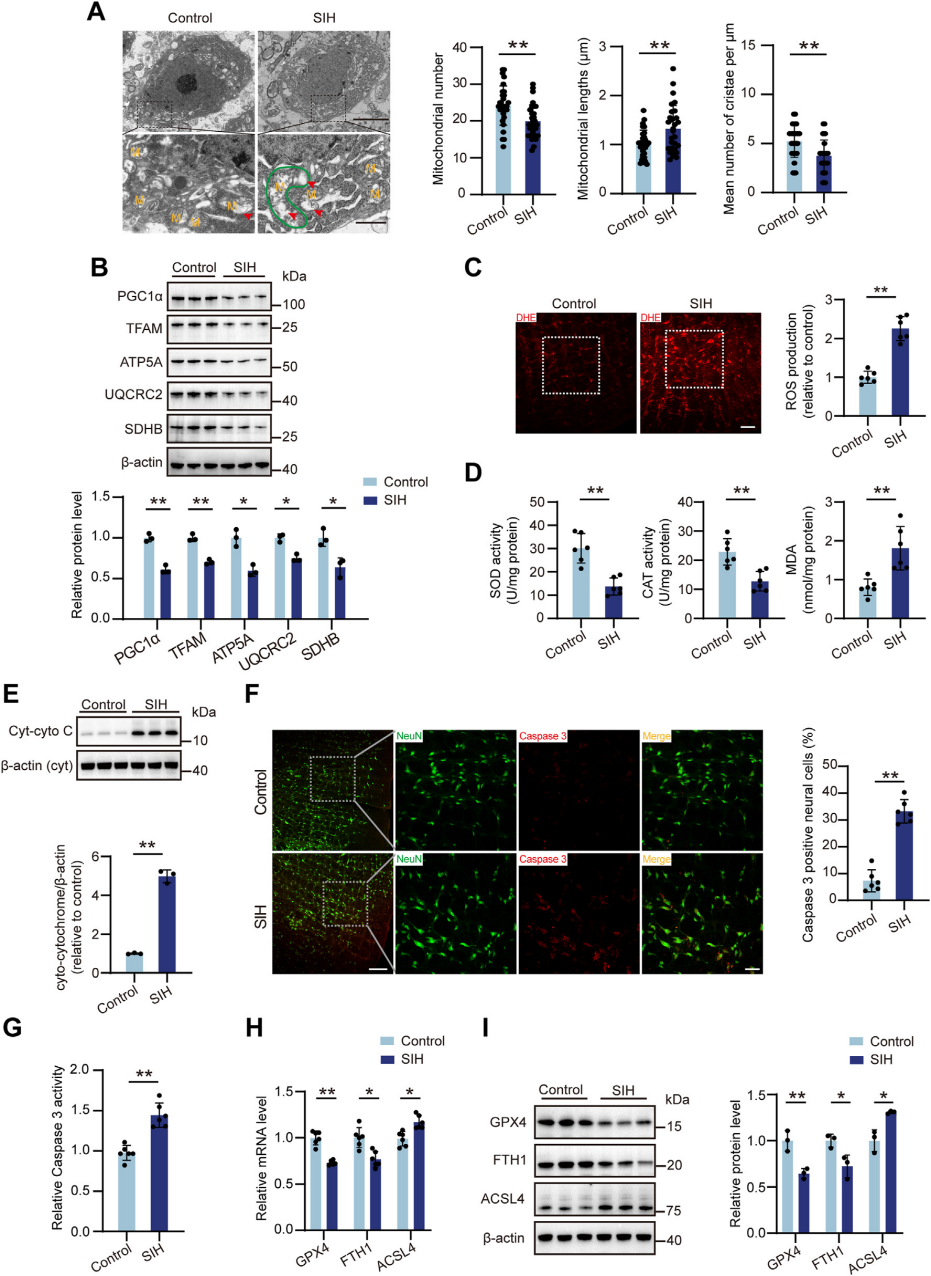
**Chronic stress elevated mitochondrial oxidative stress accompanied by the occurrence of apoptosis and ferroptosis in the RVLM of rats.***A*, the ultrastructural changes in RVLM neuronal mitochondria were observed by TEM. M, mitochondria; outlined in *green* indicates swollen mitochondria; *red* arrows indicate cristae loss. Quantifying the mitochondrial number and length, and the number of mitochondrial cristae. n = 30 neurons or mitochondria from three rats per group. Scale bar represents 5 or 1 μm. *B*, the protein levels of PGC-1α, TFAM, and mitochondrial subunits, including ATP5A, UQCRC2, and SDHB, were measured by Western blot. n = 3 rats per group. *C*, ROS production was evaluated by DHE staining. n = 6 rats per group. Scale bar represents 100 μm. *D*, SOD, CAT, and MDA levels were quantified by using corresponding kits. n = 6 rats per group. *E*, the protein expression of Cyt-cyto C analyzed by Western blot. n = 3 rats per group. *F*, immunofluorescence double staining detecting the co-expression of caspase 3 and NeuN. n = 6 rats per group. Scale bar represents 100 or 50 μm. *G*, caspase 3 activity determined using a caspase 3 activity kit. n = 6 rats per group. *H* and *I*, the mRNA and protein expression of GPX4, FTH1, and ACSL4. n = 6 (mRNA levels) or 3 (protein levels) rats per group. Data are presented as mean ± SD. In (*A*), significance was tested using the unpaired two-tailed Student’s *t* test. In *B*-*I*, significance was tested using the Mann-Whitney U test. ∗*p* < 0.05, ∗∗*p* < 0.01. CAT, catalase; Cyt-cyto C, cytoplasmic cytochrome C; DHE, dihydroethidium; MDA, malondialdehyde; ROS, reactive oxygen species; RVLM, rostral ventrolateral medulla; SD, standard deviation; SOD, superoxide dismutase; TEM, transmission electron microscopy.

To determine whether mitochondrial alterations were involved in the modulation of RVLM neuron activity in rats, a half-month of continuous chronic stress in the rats was employed. Initially, we investigated the effects of chronic stress on mitochondrial morphology and homeostasis within RVLM neurons. Ultrastructural analysis of neuronal mitochondria in SIH rats revealed notable alterations compared to normal controls. RVLM neurons in SIH rats exhibited noticeable mitochondrial swelling (outlined in green) and increased loss of cristae (red arrows). Transmission electron microscopy (TEM) further confirmed a reduction in the mitochondrial count and cristae number in SIH rats, accompanied by an increase in mitochondrial length (Fig. 1*A*). Mitochondrial biogenesis plays a critical role in maintaining mitochondrial function (34). Western blot analysis demonstrated significantly lower levels of PGC-1α and TFAM, key regulators of mitochondrial biogenesis (35), in SIH groups than control groups (Fig. 1*B*). Furthermore, the protein expressions of several crucial mitochondrial subunits, including ATP5A, UQCRC2, and SDHB, were also downregulated in SIH rats, suggesting compromised mitochondrial metabolism (Fig. 1*B*). Mitochondria, serving as a crucial producer of ROS, show a tight linkage between their dysfunction and the enhancement of oxidative stress levels (36). ROS production within the RVLM was assessed using dihydroethidium (DHE) fluorescence dye staining. Results indicated elevated ROS levels in SIH rats, evidenced by an increase in DHE fluorescence intensity (Fig. 1*C*). Concurrently, the antioxidant enzyme activities of superoxide dismutase (SOD) and catalase (CAT) were significantly decreased in SIH rats, while malondialdehyde (MDA) content, a marker of lipid peroxidation, was markedly increased (Fig. 1*D*). Subsequently, Western blot analysis revealed a significant elevation in cytoplasmic cytochrome C (Cyt-cyto C) levels in SIH rats compared to normal controls (Fig. 1*E*), suggesting mitochondrial dysfunction and release of cytochrome C into the cytosol in SIH rats. This process might initiate caspase 3 activation, driving the mitochondrial apoptotic pathway. Immunofluorescence analysis and caspase 3 activity assays also confirmed a substantial increase in both caspase 3–positive neuronal cells (Fig. 1*F*) and caspase 3 activity (Fig. 1*G*) within the RVLM region of SIH rats compared to controls. To quantify ferroptosis, we examined the expression levels of key ferroptosis-related factors. Notably, mRNA and protein levels of GPX4 and FTH1 were significantly reduced in SIH rats, while ACSL4 levels were markedly elevated (Fig. 1, *H* and *I*). These findings collectively suggest that chronic stress–induced mitochondrial morphological abnormalities and functional disruptions within RVLM neurons exacerbate oxidative stress, ultimately contributing to both apoptosis and ferroptosis.

### RSV attenuates SIH *via* RVLM microinjection

**Figure 2:**
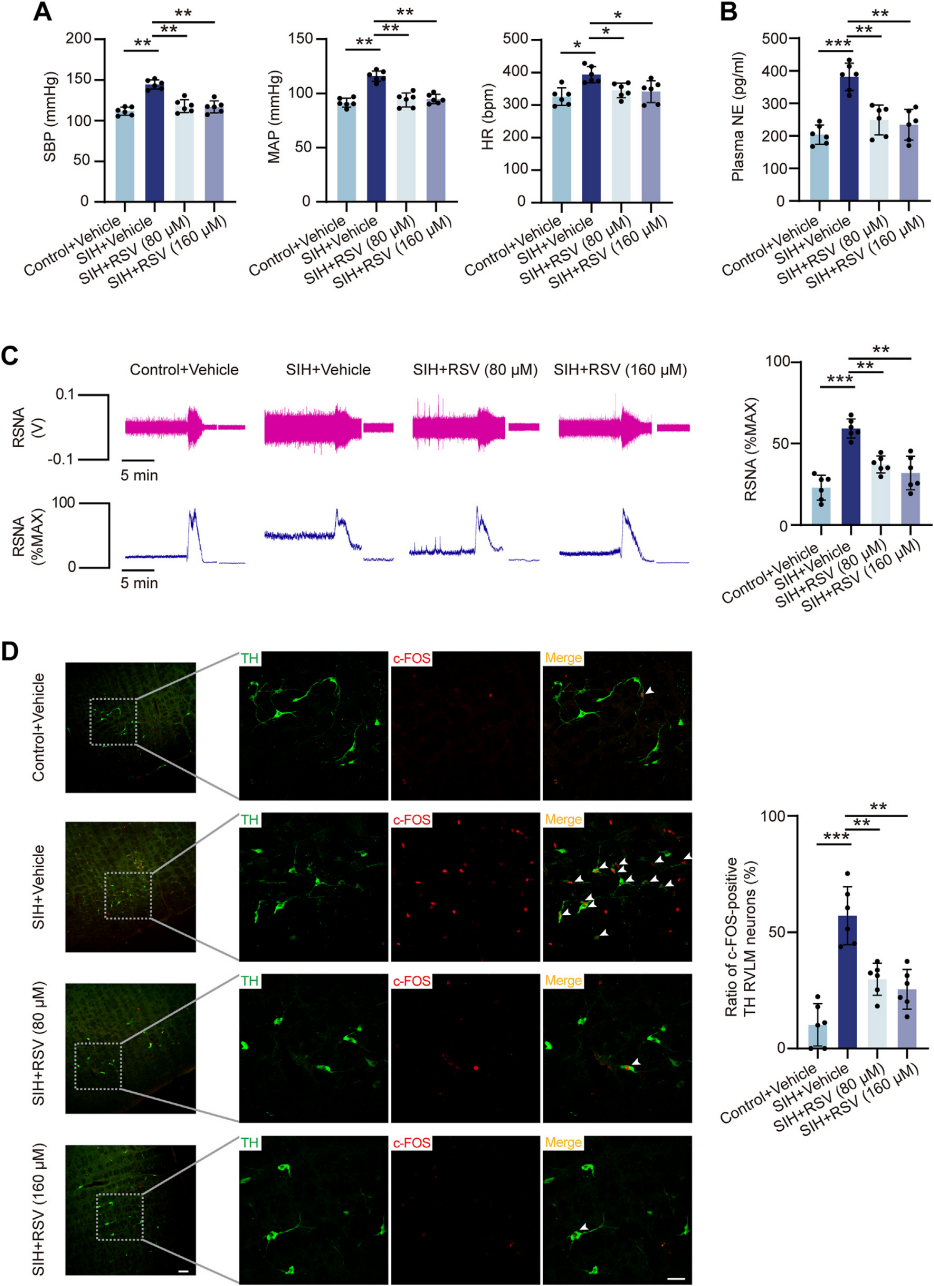
**RSV administration in the RVLM of rats improved the symptoms of SIH.***A*, the femoral artery cannulation method performed to detect the BP and HR in rats after RSV intervention. n = 6 rats per group. *B* and *C*, plasma NE ELISA and RSNA recording tests used to observe the effect of RSV treatment on sympathetic nerve activity. n = 6 rats per group. *D*, neuronal excitability in the RVLM tested by immunostaining with c-Fos and TH. n = 6 rats per group. Scale bar represents 100 or 50 μm. Data are presented as mean ± SD. In (*A–D*), significance was evaluated using the Kruskal–Wallis test and *post hoc* Dunn test. ∗*p* < 0.05, ∗∗*p* < 0.01, ∗∗∗*p* < 0.001. BP, blood pressure; ELISA, enzyme-linked immunosorbent assay; HR, heart rate; NE, norepinephrine; RSNA, renal sympathetic nerve activity; RSV, resveratrol; RVLM, rostral ventrolateral medulla; SD, standard deviation; SIH, stress-induced hypertension; TH, tyrosine hydroxylase.

To investigate the therapeutic potential of RSV on SIH in rats, RSV was administered bilaterally into the RVLM *via* microinjections on days 10 and 15 in rats after stress exposure. Treatment with RSV at concentrations of 80 μM and 160 μM resulted in a significant decrease in both BP and heart rate (HR) levels in SIH rats (Fig. 2*A*). Next, the effects of RSV on sympathetic nerve activity were assessed by measuring plasma norepinephrine (NE) and renal sympathetic nerve activity (RSNA). RSV treatment effectively restored both plasma NE levels and RSNA to normal levels in SIH rats (Fig. 2, *B* and *C*). Furthermore, the impact of RSV on RVLM neuronal activity was evaluated using immunofluorescence staining. The results indicated that the RSV administration in SIH rats significantly reduced the number of c-Fos−expressing tyrosine hydroxylase (TH)-positive neurons within the RVLM (Fig. 2*D*). These findings suggest that RSV exerts antihypertensive effects by reducing neuronal excitability in the RVLM and suppressing sympathetic tone, thereby mediating the neurogenic regulation of SIH.

### RSV mitigates mitochondria-associated oxidative stress, apoptosis, and ferroptosis in the RVLM of the SIH rats

**Figure 3:**
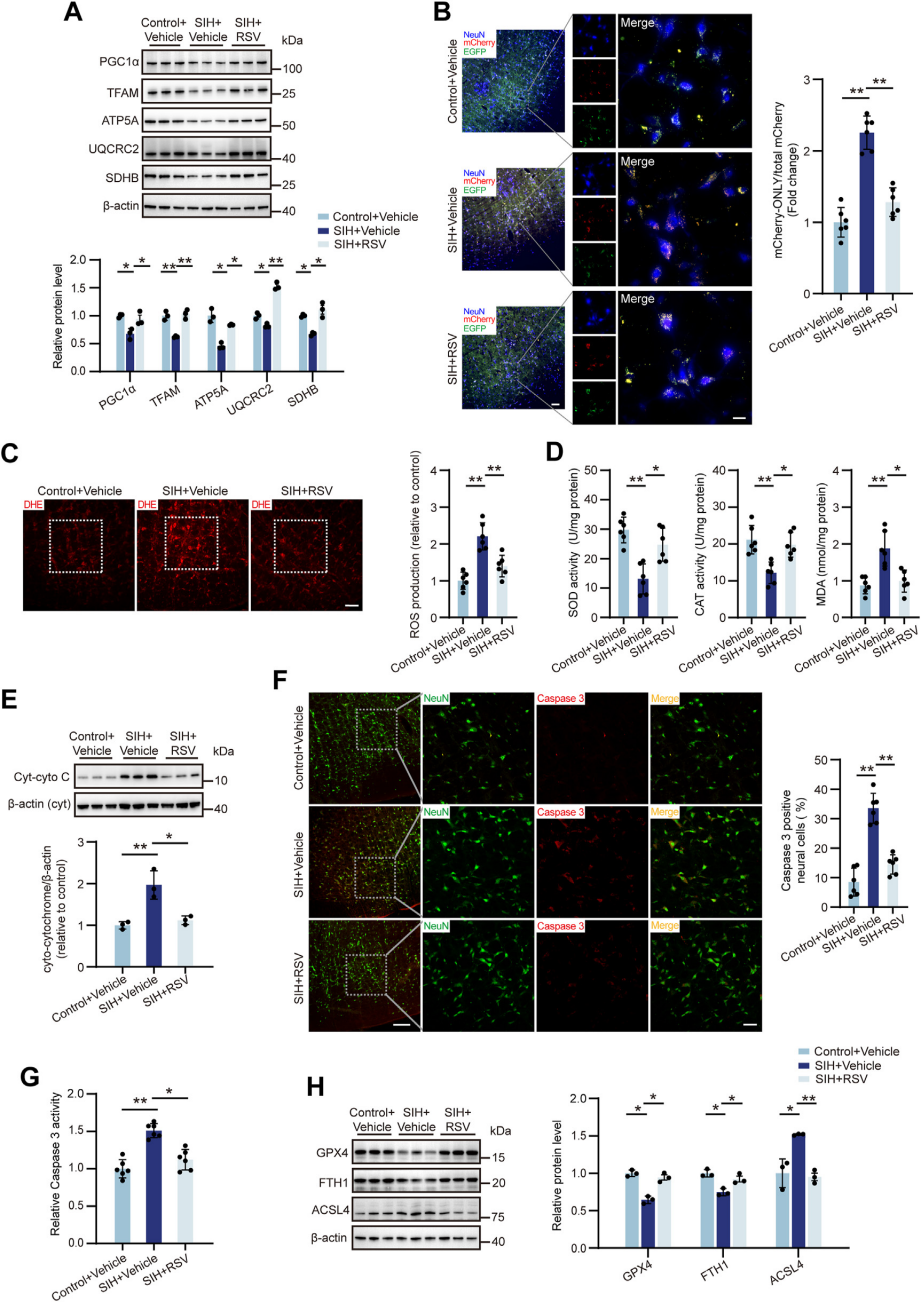
**RSV attenuated mitochondrial oxidative stress, apoptosis, and ferroptosis in the RVLM of SIH rats.***A*, effects of RSV on PGC-1α, TFAM, and mitochondrial subunits including ATP5A, UQCRC2, and SDHB protein expression. n = 3 rats per group. *B*, effects of RSV on the degree of mitochondrial degradation in rats by assessing the ratio value of mCherry-ONLY/total mCherry puncta. n = 6 rats per group. Scale bar represents 100 or 20 μm. *C* and *D*, effects of RSV on ROS, SOD, CAT, and MDA levels. n = 6 rats per group. Scale bar represents 100 μm. *E*, effect of RSV on Cyt-cyto C protein expression. n = 3 rats per group. *F* and *G*, effect of RSV on caspase 3 expression and activity. n = 6 rats per group. Scale bar represents 100 or 50 μm. *H*, effects of RSV on GPX4, FTH1, and ACSL4 protein expression. n = 3 rats per group. Data are presented as mean ± SD. In (*A–H*), significance was evaluated using the Kruskal–Wallis test and *post hoc* Dunn test. ∗*p* < 0.05, ∗∗*p* < 0.01. CAT, catalase; Cyt-cyto C, cytoplasmic cytochrome C; DHE, dihydroethidium; MDA, malondialdehyde; ROS, reactive oxygen species; RSV, resveratrol; RVLM, rostral ventrolateral medulla; SD, standard deviation; SIH, stress-induced hypertension.

To elucidate the protective effects of RSV in SIH, we initially assessed its impact on mitochondrial homeostasis within the RVLM. Administration of RSV (80 μM) to SIH rats significantly upregulated PGC-1α, TFAM, and mitochondrial subunits including ATP5A, UQCRC2, and SDHB in the RVLM (Fig. 3*A*). Mitochondrial fission dynamics and mitophagy are crucial for maintaining optimal mitochondrial function (37). Western blot analysis revealed that chronic stress reduced mitochondrial fission and promoted fusion in the RVLM of rats, as evidenced by downregulation of the fission-related protein DRP1 and upregulation of the fusion-related protein MFN2 levels (Fig. S1*A*). RSV treatment reversed this trend by inducing an increase in DRP1 expression without significantly affecting MFN2 levels. Furthermore, heightened levels of PINK1 and Parkin proteins in SIH rats suggested activation of the PINK1/Parkin-mediated mitophagy pathway (Fig. S1*B*). RSV administration further augmented Parkin expression, while PINK1 protein levels remained relatively unchanged.

To directly assess mitochondrial degradation, a tandem fluorophore reporter system incorporating acid-sensitive EGFP and acid-resistant mCherry was employed (38). EGFP fluorescence was quenched in the acidic lysosomal environment, whereas mCherry fluorescence persisted. Consequently, the presence of mCherry-ONLY (mCherry-positive/EGFP-negative) puncta indicated degrading mitochondria. A significant increase was observed in mCherry-ONLY puncta within RVLM neurons of SIH rats compared to control rats (Fig. 3*B*). Notably, RSV treatment significantly reduced the number of mCherry-ONLY puncta in SIH rats, suggesting its ability to counteract mitochondrial damage induced by chronic stress. Oxidative stress markers, including ROS and MDA, were markedly elevated in SIH rats compared to controls (Fig. 3, *C* and *D*). Conversely, the activities of antioxidant enzymes SOD and CAT were diminished in SIH rats, but RSV administration reversed these alterations.

To investigate the potential of RSV to counteract mitochondrial oxidative stress damage, we assessed key markers of apoptosis and ferroptosis. RSV treatment significantly reduced Cyt-cyto C expression compared to the SIH plus vehicle group (Fig. 3*E*). Additionally, RSV treatment reversed the increased number of caspase 3–positive neural cells and caspase 3 activity observed in the RVLM of SIH rats, confirming its capacity to alleviate mitochondria-mediated apoptosis (Fig. 3, *F* and *G*). In contrast to GPX4 and FTH1 levels, ACSL4 expression was significantly higher in the SIH plus vehicle group than both the control plus vehicle and SIH plus RSV groups (Fig. 3*H*). These findings demonstrate that RSV treatment effectively counteracts the detrimental effects of chronic stress on the RVLM, suppressing oxidative stress damage, apoptosis, and ferroptosis.

### RSV ameliorates mitochondrial dysfunction, apoptosis, and ferroptosis *in vitro*

**Figure 4:**
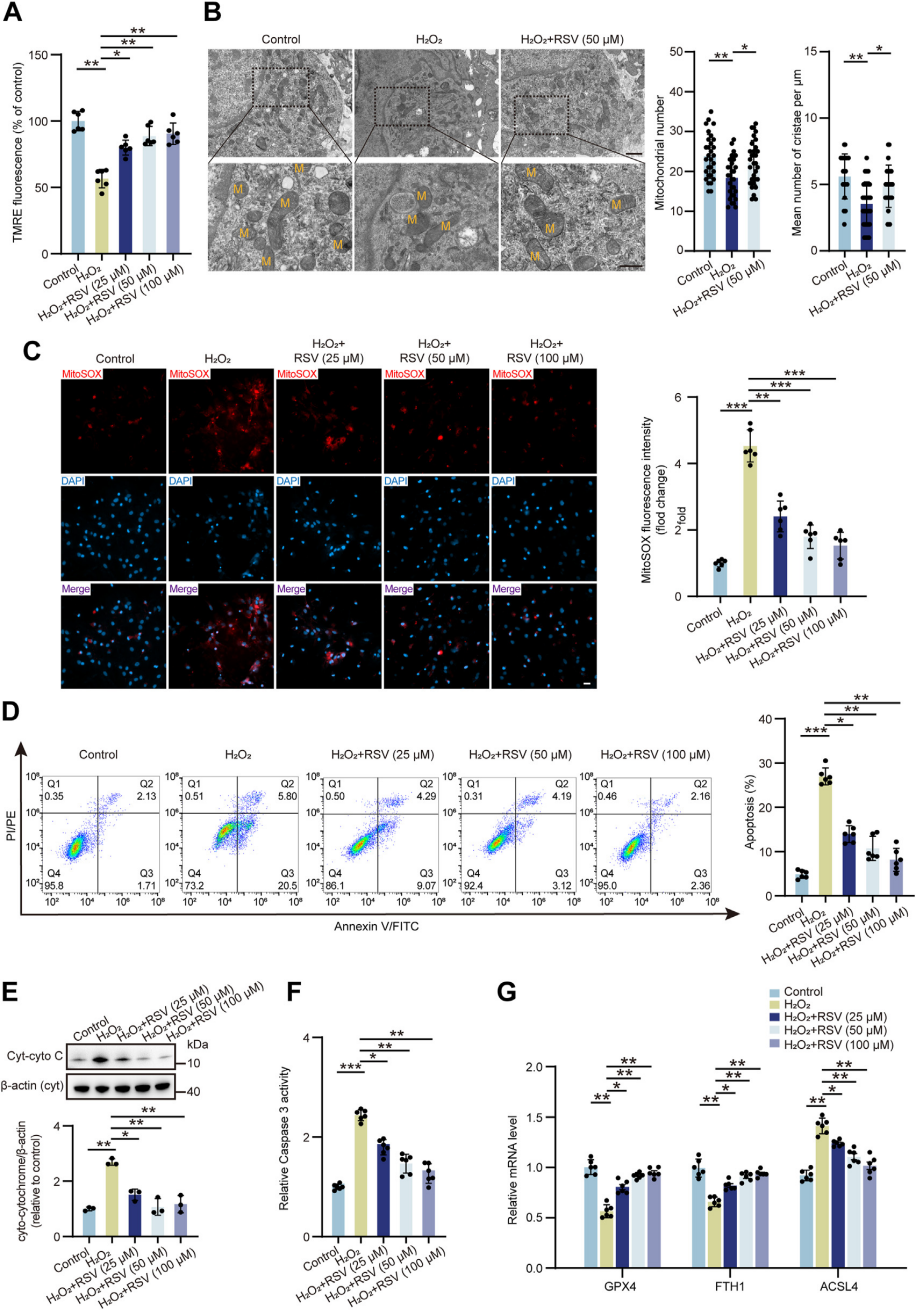
**Mitochondrial oxidative stress, apoptosis, and ferroptosis in RVLM primary neurons induced by H_2_O_2_ were alleviated by RSV.***A*, MMP detected by monitoring TMRE fluorescence intensity. n = 6 of independent cell culture preparations. *B*, the mitochondria within the cell body of RVLM primary neurons imagined by transmission electron microscopy. Quantification of mitochondrial count and cristae number. n = 30 RVLM primary neurons or mitochondria from three of independent cell culture preparations. Scale bar represents 1 μm or 500 nm. *C*, mitochondrial ROS production was determined using MitoSOX staining. n = 6 of independent cell culture preparations. Scale bar represents 20 μm. *D*, apoptosis determined using flow cytometry. n = 6 of independent cell culture preparations. *E*, the protein expression of Cyt-cyto C measured using Western blot. n = 3 of independent cell culture preparations. *F*, caspase 3 activity determined using caspase 3 activity kit. n = 6 of independent cell culture preparations. *G*, the mRNA expression of *Gpx4*, *Fth1*, and *Acsl4* detected by qRT-PCR. n = 6 of independent cell culture preparations. Data are presented as mean ± SD. In (*B*), significance was evaluated using the one-way ANOVA and *post hoc* Bonferroni test. In (*A* and *C–G*), significance was evaluated using the Kruskal–Wallis test and *post hoc* Dunn test. ∗*p* < 0.05, ∗∗*p* < 0.01, ∗∗∗*p* < 0.001. Cyt-cyto C, cytoplasmic cytochrome C; M, mitochondria; MMP, mitochondrial membrane potential; qRT-PCR, quantitative real-time PCR; ROS, reactive oxygen species; RSV, resveratrol; RVLM, rostral ventrolateral medulla; SD, standard deviation.

To investigate the protective effects of RSV against mitochondrial dysfunction, apoptosis, and ferroptosis, *in vitro* experiments were conducted using RVLM primary neurons. H_2_O_2_ was utilized to induce oxidative stress (39, 40), with the optimal concentration determined *via* cell counting kit-8 (CCK-8) assay (Fig. S2). Subsequent experiments employed 100 μM H_2_O_2_. RSV treatment (25, 50, and 100 μM) significantly attenuated H_2_O_2_-induced mitochondrial membrane potential (MMP) decline as assessed by TMRE staining (Fig. 4*A*). TEM revealed that H_2_O_2_ exposure resulted in a decrease in mitochondria density and cristae number, effects reversed by RSV treatment (Fig. 4*B*). Furthermore, MitoSox staining demonstrated that RSV treatment significantly reduced the H_2_O_2_-induced increase in mitochondrial ROS production (Fig. 4*C*). Flow cytometry analysis of apoptosis detection, alongside Cyt-cyto C expression and caspase 3 activity assessments, disclosed that H_2_O_2_-induced RVLM neuronal apoptosis and increased both Cyt-cyto C expression and caspase 3 activity (Fig. 4, *D*–*F*). These effects were largely abolished by RSV treatment. To evaluate ferroptosis, *Gpx4* and *Fth1* mRNA levels, along with *Acsl4* expression, were examined. H_2_O_2_ exposure decreased *Gpx4* and *Fth1* while increasing *Acsl4* expression (Fig. 4*G*), a trend reversed by RSV treatment. Collectively, these findings demonstrate that RSV effectively mitigates H_2_O_2_-induced mitochondrial oxidative stress, apoptosis, and ferroptosis in RVLM primary neurons.

### The AMPK/Sirt3 signaling mediates RSV-induced mitochondrial protection in SIH rats

**Figure 5:**
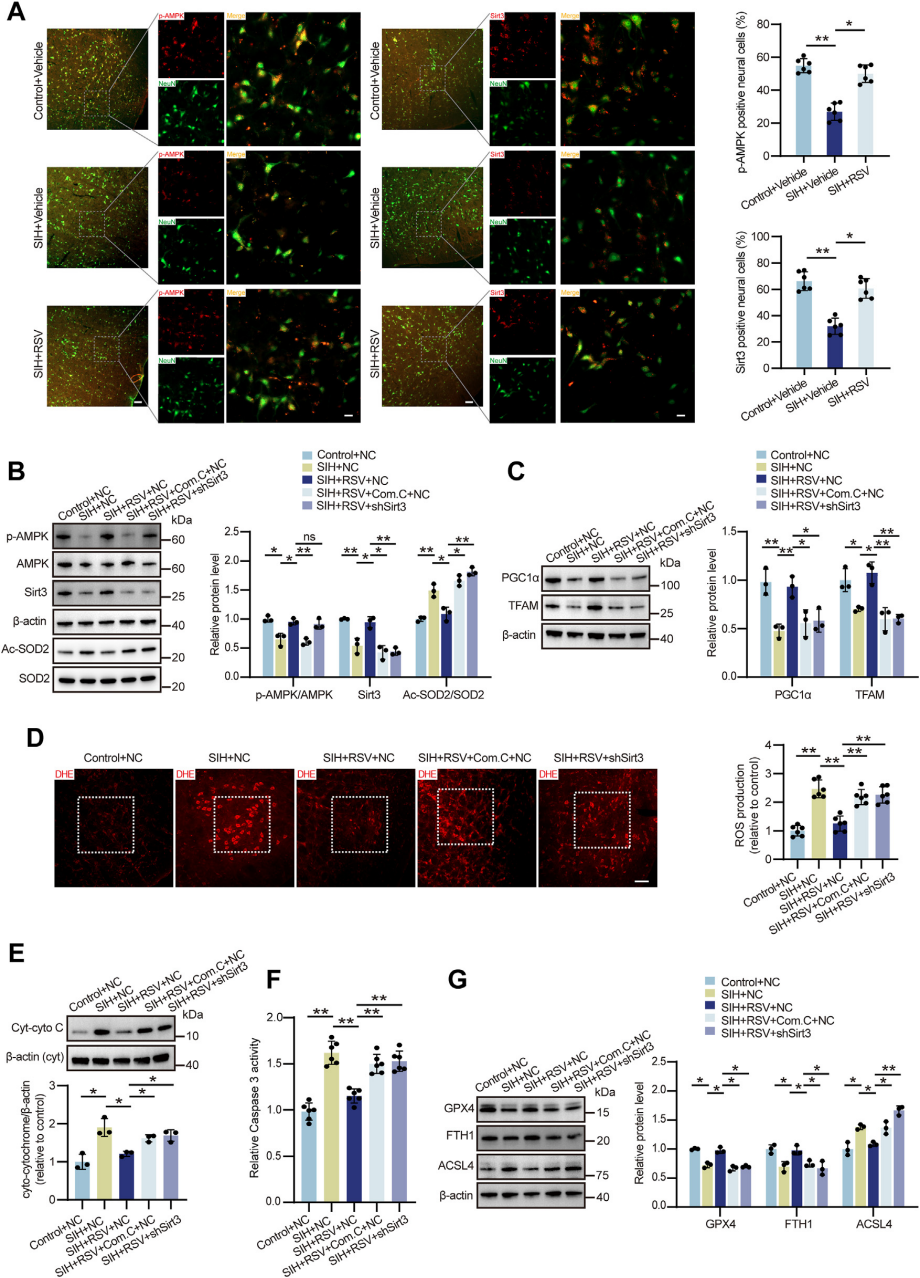
**AMPK/Sirt3 signaling mediated the protective effects of RSV against mitochondrial oxidative stress, apoptosis, and ferroptosis in the RVLM of SIH rats.***A*, immunofluorescence double staining performed to detect the co-expression of p-AMPK and NeuN, as well as the co-expression of Sirt3 and NeuN. n = 6 rats per group. Scale bar represents 100 or 20 μm. *B*, quantitative analysis of Western blot results for p-AMPK/AMPK, Sirt3/β-actin, and Ac-SOD2/SOD2. n = 3 rats per group. *C*, the protein expression of PGC-1α and TFAM determined by Western blot. n = 3 rats per group. *D*, ROS production evaluated by DHE staining. n = 6 rats per group. Scale bar represents 100 μm. *E*, the protein expression of Cyt-cyto C was examined by Western blot analysis. n = 3 rats per group. *F*, caspase 3 activity was assessed using a caspase 3 activity kit. n = 6 rats per group. *G*, the protein expression of GPX4, FTH1, and ACSL4 analyzed by Western blot. n = 3 rats per group. Data are presented as mean ± SD. In (*A–G*), significance was evaluated using the Kruskal–Wallis test and *post hoc* Dunn test. n.s., no significance. ∗*p* < 0.05, ∗∗*p* < 0.01. Cyt-cyto C, cytoplasmic cytochrome C; DHE, dihydroethidium; ROS, reactive oxygen species; RSV, resveratrol; RVLM, rostral ventrolateral medulla; SD, standard deviation; SIH, stress-induced hypertension.

To elucidate the molecular mechanisms underlying RSV's neuroprotective effects, we investigated the role of AMPK/Sirt3 signaling. Our previous work demonstrated that AMPK activation of Sirt3 is crucial for ameliorating mitochondrial dysfunction and oxidative stress in SIH (18). Western blot and immunofluorescence analyses revealed significantly reduced p-AMPK and Sirt3 levels in the RVLM of SIH rats treated with vehicle compared to those receiving RSV treatment (Figs. 5*A* and S3). Conversely, pharmacological inhibition of AMPK with compound C (Com.C) abolished the RSV-mediated upregulation of both p-AMPK and Sirt3 (Fig. 5*B*). *Sirt3* knockdown using shRNA significantly attenuated the RSV-induced increase in Sirt3 expression but did not affect p-AMPK levels, indicating that Sirt3 acts downstream of AMPK and mediates RSV's beneficial effects (Fig. 5*B*).

Since activated Sirt3 deacetylates SOD2, enhancing its enzymatic activity, we assessed SOD2 acetylation levels. Western blot analysis demonstrated increased SOD2 acetylation in the RVLM of SIH rats, which was significantly reduced by RSV treatment through the AMPK/Sirt3 signaling activation (Fig. S3). Furthermore, both AMPK and Sirt3 inhibition prevented RSV-induced SOD2 deacetylation (Fig. 5*B*).

To examine the impact of AMPK/Sirt3 inactivation on RSV's neuroprotective effects, we assessed mitochondrial function, oxidative stress, apoptosis, and ferroptosis. Com.C or *Sirt3* shRNA treatment significantly attenuated RSV-mediated preservation of mitochondrial function compared to the SIH plus RSV plus NC shRNA group (Figs. 5*C* and S4). RSV treatment inhibited ROS generation, MDA content accumulation, and SOD/CAT activity decrease in SIH rats, but these effects were largely abolished by AMPK or Sirt3 inhibition (Figs. 5*D* and S5). Moreover, AMPK/Sirt3 inactivation disrupted RSV's ability to reduce apoptosis levels as evidenced by the upregulation of Cyt-cyto C and caspase 3 activity (Fig. 5, *E* and *F*). Finally, RSV administration increased GPX4 and FTH1 expression and reduced ACSL4 levels in SIH rats. These beneficial effects were suppressed by Com.C or *Sirt3* shRNA treatment (Fig. 5*G*). Collectively, these findings display that RSV's neuroprotective effects against mitochondrial oxidative stress, apoptosis, and ferroptosis in the RVLM of SIH rats are dependent on the AMPK/Sirt3 pathway activation.

### The AMPK/Sirt3 signaling is essential for RSV's neuroprotective effects against H_2_O_2_-induced damage

**Figure 6:**
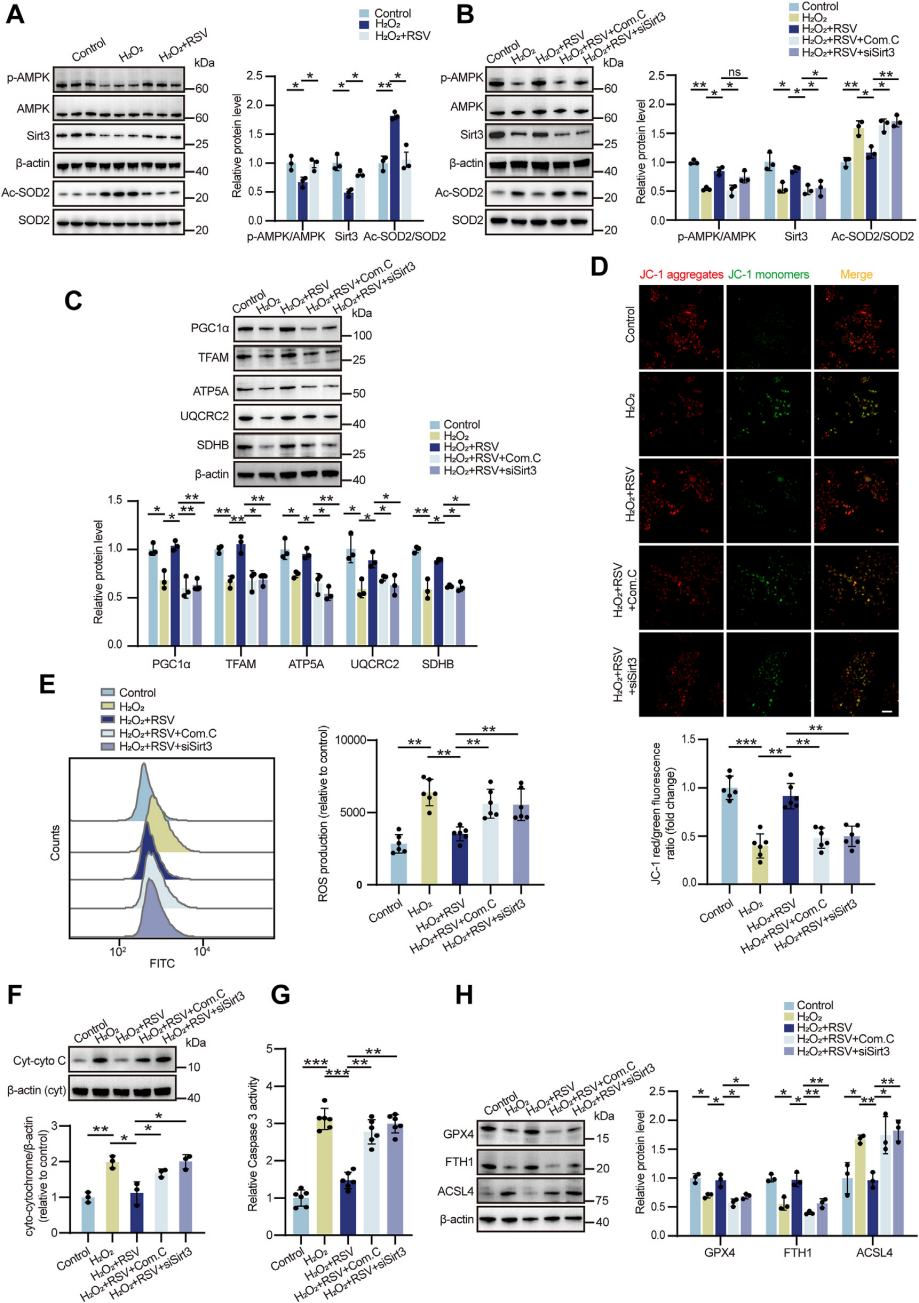
**RSV restricted H_2_O_2_-induced mitochondrial oxidative stress, apoptosis, and ferroptosis by modulating the AMPK/Sirt3 pathway in N2a cells.***A* and *B*, quantitative analysis of Western blot results for p-AMPK/AMPK, Sirt3/β-actin, and Ac-SOD2/SOD2. n = 3 of independent cell culture preparations. *C*, the protein expression of PGC-1α, TFAM, and mitochondrial subunits including ATP5A, UQCRC2, and SDHB examined by Western blot. n = 3 of independent cell culture preparations. *D*, MMP determined using the JC-1 staining. n = 6 of independent cell culture preparations. Scale bar represents 50 μm. *E*, ROS production determined using flow cytometry. n = 6 of independent cell culture preparations. *F*, the protein expression of Cyt-cyto C performed using Western blot. n = 3 of independent experiments. *G*, caspase 3 activity measured using a caspase 3 activity kit. n = 6 of independent experiments. *H*, the protein expression of GPX4, FTH1, and ACSL4 measured by Western blot. n = 3 of independent cell culture preparations. Data are presented as mean ± SD. In (*A-H*), significance was evaluated using the Kruskal–Wallis test and *post hoc* Dunn test. n.s., no significance. ∗*p* < 0.05, ∗∗*p* < 0.01, ∗∗∗*p* < 0.001. Cyt-cyto C, cytoplasmic cytochrome C; MMP, mitochondrial membrane potential; ROS, reactive oxygen species; RSV, resveratrol; SD, standard deviation.

To elucidate the role of the AMPK/Sirt3 pathway in mediating RSV's neuroprotective effects, we conducted *in vitro* experiments using mouse neuroblastoma N2a cells. Treatment with various concentrations of RSV (25, 50, and 100 μM) significantly increased both the p-AMPK/AMPK ratio and Sirt3 protein levels compared to untreated controls (Fig. S6, *A* and *B*). Conversely, cotreatment with Com.C (10 and 20 μM) effectively suppressed RSV-induced AMPK/Sirt3 pathway activation. CCK-8 assay demonstrated that varying doses of H_2_O_2_ significantly reduced N2a cell viability (Fig. S7). We subsequently utilized a model of oxidative stress induced by 100 μM H_2_O_2_, as previously described (41), to investigate RSV's protective effects. Successful knockdown of *Sirt3* expression in N2a cells was confirmed *via* quantitative real-time PCR (qRT-PCR) and Western blot analysis (Fig. S8, *A* and *B*). RSV treatment significantly elevated AMPK phosphorylation and Sirt3 protein expression while simultaneously decreasing SOD2 acetylation levels compared to the H_2_O_2_-treated group (Fig. 6*A*). Cotreatment with Com.C or *Sirt3* siRNA, however, abrogated these effects, confirming the critical role of the AMPK/Sirt3 pathway in mediating RSV's neuroprotective actions (Fig. 6*B*).

To further investigate the impact of AMPK/Sirt3 signaling on mitochondrial function, we assessed protein levels of key mitochondrial biogenesis markers. Western blot analysis revealed that Com.C and *Sirt3* siRNA cotreatment significantly reduced protein levels of PGC-1α, TFAM, as well as mitochondrial subunits ATP5A, UQCRC2, and SDHB compared to the H_2_O_2_ plus RSV group (Fig. 6*C*). While RSV treatment effectively mitigated H_2_O_2_-induced declines in MMP, this protective effect was reversed by Com.C or *Sirt3* siRNA treatment (Fig. 6*D*). Flow cytometry confirmed elevated ROS levels in cells cotreated with Com.C and *Sirt3* siRNA compared to the H_2_O_2_ plus RSV group (Fig. 6*E*). Furthermore, RSV attenuated H_2_O_2_-induced decreases in SOD and CAT activities, as well as MDA accumulation (Fig. S9), but these beneficial effects were reversed by AMPK/Sirt3 pathway inhibition.

Analysis of apoptosis and ferroptosis levels further supported the role of the AMPK/Sirt3 signaling. RSV treatment attenuated H_2_O_2_-induced elevations in Cyt-cyto C protein expression and caspase 3 activity (Fig. 6, *F* and *G*). However, this anti-apoptotic effect was significantly hindered by Com.C or *Sirt3* siRNA cotreatment. We also observed that RSV's ability to prevent ferroptosis was compromised in the absence of AMPK/Sirt3 signaling, as evidenced by decreased GPX4 and FTH1 protein levels and increased ACSL4 expression (Fig. 6*H*). Collectively, these data establish the crucial role of the AMPK/Sirt3 pathway in mediating RSV's neuroprotective effects against mitochondrial oxidative stress, apoptosis, and ferroptosis.

### The AMPK/Sirt3 activation mediates the antihypertensive effects of RSV in SIH rats

**Figure 7:**
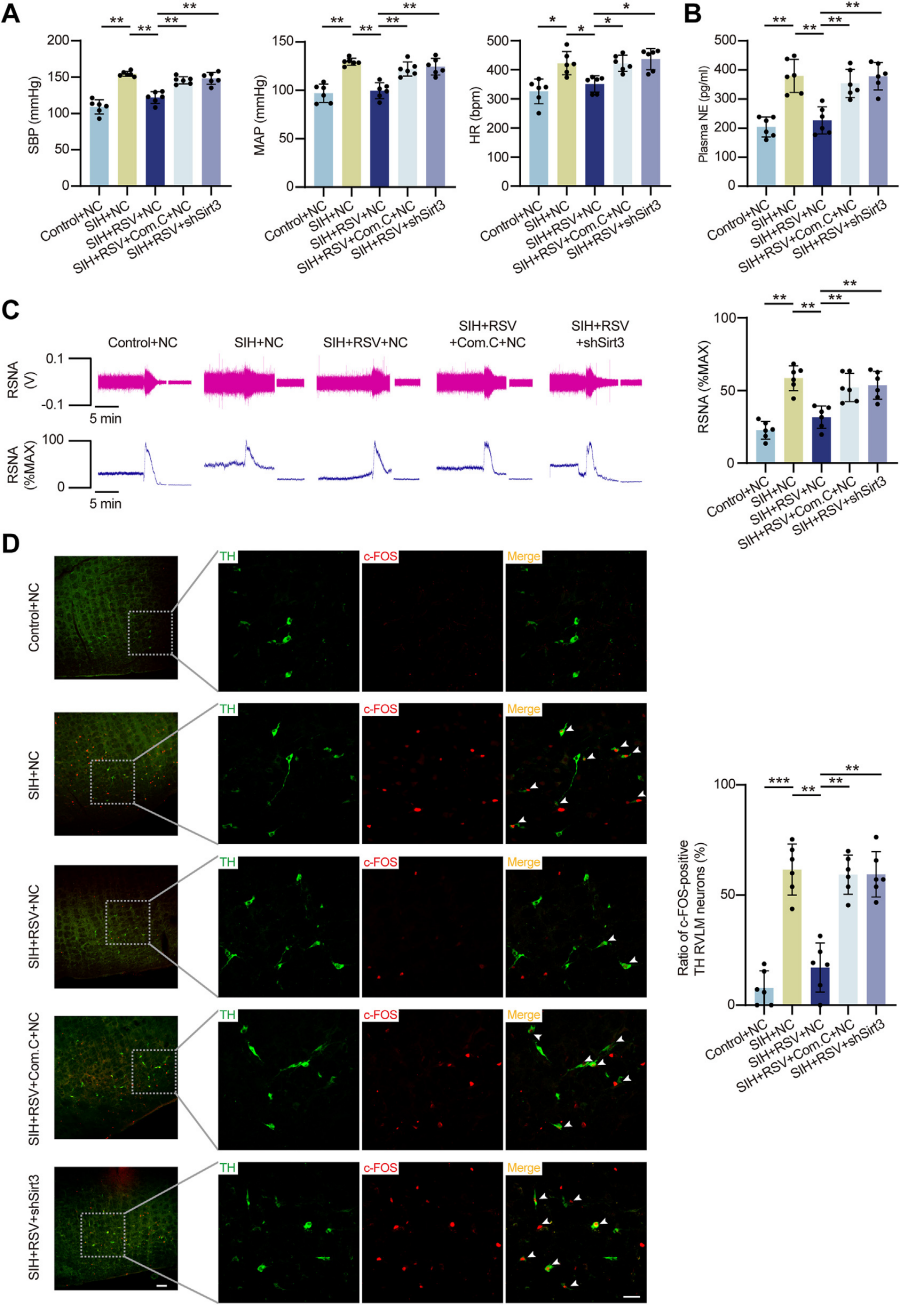
**RSV ameliorated mitochondria-related oxidative stress, apoptosis, and ferroptosis by activating the AMPK/Sirt3 pathway, inhibiting RVLM neuronal excitability and sympathetic overdrive, and ultimately slowing SIH progression.***A*, the BP and HR levels measured in rats. n = 6 rats per group. *B* and *C*, plasma NE ELISA and RSNA evaluated in rats. n = 6 rats per group. *D*, the percentage of c-Fos colocalized with TH. n = 6 rats per group. Scale bar represents 100 or 50 μm. Data are presented as mean ± SD. In (*A–D*), significance was evaluated using the Kruskal–Wallis test and *post hoc* Dunn test. ∗*p* < 0.05, ∗∗*p* < 0.01, ∗∗∗*p* < 0.001. BP, blood pressure; ELISA, enzyme-linked immunosorbent assay; HR, heart rate; NE, norepinephrine; RSNA, renal sympathetic nerve activity; RSV, resveratrol; RVLM, rostral ventrolateral medulla; SD, standard deviation; SIH, stress-induced hypertension; TH, tyrosine hydroxylase.

To investigate the potential antihypertensive effects of RSV in SIH *via* activation of the AMPK/Sirt3 pathway, we assessed physiological and biochemical parameters. RSV administration into the RVLM significantly reduced both BP and HR levels in SIH rats compared to controls. This hypotensive effect was abolished by co-administration of the AMPK inhibitor or *Sirt3* knockdown (Fig. 7*A*). Sympathetic activity was evaluated through plasma NE ELISA assays and recordings of RSNA. RSV treatment significantly reduced elevated NE levels and RSNA observed in SIH rats. However, these reductions were attenuated by both AMPK inhibition and Sirt3 downregulation (Fig. 7, *B* and *C*). Immunofluorescent staining was utilized to assess RVLM neuronal excitability, revealing that RSV-mediated reduction in c-Fos−expressing TH-positive neurons within the RVLM was inhibited by AMPK/Sirt3 pathway inactivation in SIH rats (Fig. 7*D*). Collectively, these findings indicate that RSV mitigates mitochondrial oxidative stress, apoptosis, and ferroptosis through activation of the AMPK/Sirt3 pathway, contributing to the suppression of hypertension development in SIH rats.

## Discussion

While numerous studies highlight the antioxidant and neuroprotective properties of RSV, particularly their ability to activate AMPK signaling, less is known about their specific effects on mitochondrial homeostasis within the RVLM during SIH. This study demonstrates that RSV administration effectively alleviates oxidative stress in mitochondria and inhibits apoptosis and ferroptosis within the RVLM of SIH rats. These beneficial effects appear to be mediated by the activation of the AMPK/Sirt3 pathway.

Stress, particularly chronic psychological stress induced by factors such as anxiety, high-intensity workloads, and noise pollution, is associated with an increased risk of hypertension (3, 4, 5). Long-term electrical foot shocks combined with noisy stimulation act as conditioned stimuli, inducing both psychological and physiological stress in rats, ultimately leading to elevated blood pressure responses. This stress-induced increase in blood pressure is primarily mediated by the sympathetic nervous system, and neurons within the cardiovascular center, particularly those located in the RVLM, exert a critical influence on sympathetic outflow and the progression of SIH (8, 9, 18, 19, 42).

Mitochondria are essential organelles vital for maintaining normal neuronal function (43). Our previous research reported that mitochondrial injury is correlated with the etiology of SIH (18, 19). In this study, we observed compromised mitochondrial homeostasis in the RVLM neurons of SIH rats. This manifested as disrupted mitochondrial structure and morphology, impaired biogenesis, and altered oxidative metabolism. Furthermore, these dysfunctional mitochondria exhibited increased susceptibility to oxidative damage, contributing to neuronal apoptosis and ferroptosis.

Elevated oxidative stress is a well-established trigger for cellular apoptosis (44). Our study identified significant apoptosis in the RVLM neurons of SIH rats using various markers: increased Cyt-cyto C expression, quantification of caspase 3–positive neuronal cells, and measurement of caspase 3 activity. GPX4, a crucial antioxidant enzyme involved in ferroptosis suppression (45, 46), was found to be downregulated in these neurons. While the role of ferroptosis in various diseases is increasingly recognized (47, 48), its contribution to neurogenic regulation of hypertension remains underexplored. Our findings demonstrated significantly decreased GPX4 and FTH1 protein levels, alongside increased ACSL4 expression within the RVLM of SIH rats, indicating that chronic stress exacerbated oxidative stress–related ferroptosis in this brain region. These results highlight the crucial role of dysfunctional mitochondria in RVLM neurons, driving both apoptosis and ferroptosis and contributing significantly to SIH development.

Polyphenols, renowned for their antioxidant properties, have garnered attention for their potential benefits in aging and age-related diseases (49, 50). RSV, a natural polyphenol with established antioxidant capacity (51), has demonstrated promising effects in mitigating diabetes progression (52) and reversing myocardial damage induced by sepsis-induced cardiomyopathy (53). This study systematically evaluated the therapeutic efficacy of RSV against SIH. Our findings demonstrate that RSV administration significantly reduced BP, HR, plasma NE levels, RSNA activity, and the number of c-Fos−expressing TH-positive RVLM neurons in SIH rats. *In vivo* and *in vitro* studies further revealed that RSV effectively maintained mitochondrial homeostasis and ameliorated mitochondrial dysfunction, hindering SIH progression. This was evidenced by increased protein levels of mitochondrial biogenesis markers and subunits, improved mitochondrial dynamics, and rescued MMP depolarization. Furthermore, RSV treatment resulted in enhanced SOD and CAT activities, decreased ROS levels and MDA content, and suppressed apoptosis and ferroptosis. Collectively, these results demonstrate that RSV effectively mitigates SIH by alleviating oxidative stress within RVLM mitochondria and suppressing apoptosis and ferroptosis, thereby restraining sympathetic hyperactivity and lowering blood pressure.

AMPK, a ubiquitous serine/threonine protein kinase, plays a critical role in cellular metabolic balance and oxidative stress regulation (54). Several studies have highlighted the potential antioxidant and neuroprotective properties of the AMPK pathway activation by polyphenols in various diseases (55, 56, 57), suggesting its involvement in RSV's inhibitory effects on mitochondrial oxidative stress, apoptosis, and ferroptosis within RVLM neurons during SIH. Sirt3, a critical regulator of mitochondrial biogenesis and oxidative metabolism, maintains mitochondrial homeostasis (58). Previous research demonstrated that Sirt3 upregulation in the RVLM, dependent on AMPK activation, suppressed sympathetic overdrive and SIH progression (18). In both SIH rats and H_2_O_2_-stimulated N2a cells, we observed a significant decrease in the p-AMPK/AMPK ratio and Sirt3 levels, which were restored upon RSV administration. This suggests that chronic stress–induced AMPK signaling inhibition leads to reduced Sirt3 expression.

**Figure 8:**
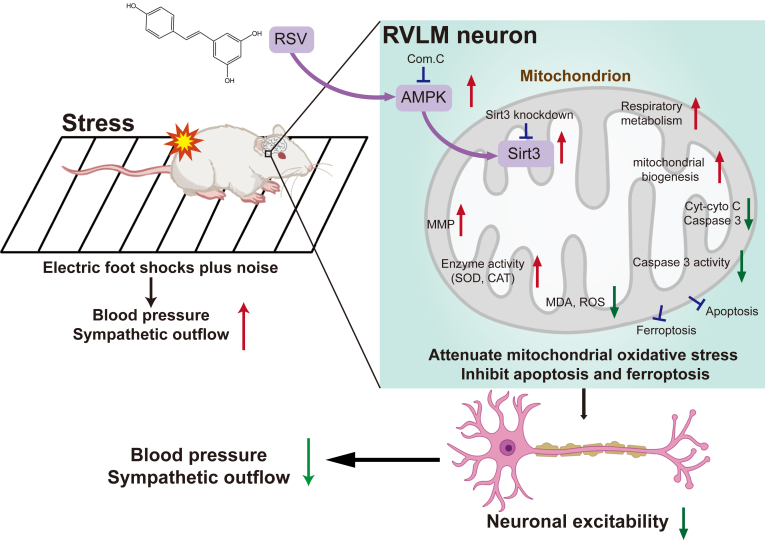
**A schematic illustration displays the hypotensive mechanisms of resveratrol in the RVLM.** The mechanisms involving the activation of the AMPK/Sirt3 pathway by RSV were instrumental in alleviating stress-induced rises in BP, sympathetic overdrive, and neuronal excitability by decreasing mitochondrial oxidative stress, apoptosis, and ferroptosis in the RVLM. BP, blood pressure; RSV, resveratrol; RVLM, rostral ventrolateral medulla.

RSV may promote Sirt3 expression by activating the AMPK signaling pathway within the RVLM, thereby contributing to its pharmacological and antihypertensive effects in SIH rats. In both *in vivo* and *in vitro* experiments, RSV's ability to alleviate mitochondrial oxidative stress and prevent apoptosis and ferroptosis was significantly hindered by an AMPK inhibitor or Sirt3 depletion. Interestingly, AMPK inhibition reversed the increase in p-AMPK and Sirt3 expression induced by RSV, while Sirt3 knockdown abolished the protective effects of RSV without significantly altering AMPK phosphorylation levels. Furthermore, evidence indicated that RSV-triggered AMPK/Sirt3 activation promoted the deacetylation of SOD2, a downstream target of Sirt3, an effect impeded by AMPK inhibition or Sirt3 depletion. These findings strongly support Sirt3 as a crucial downstream factor of AMPK in mediating RSV's sympathetic regulation and hypotensive action. Therefore, we conclude that RSV effectively impedes SIH development through its regulation of RVLM neuronal mitochondrial oxidative stress, apoptosis, and ferroptosis, with the underlying mechanism involving RSV-induced Sirt3 expression *via* AMPK activation (Fig. 8). These findings highlight RSV's potential as a novel therapeutic strategy for managing SIH.

However, further research is warranted to address several limitations of this study. Firstly, while our investigation demonstrated the efficacy of short-term RSV delivery within the RVLM, long-term studies are necessary to evaluate its sustained antihypertensive effects and potential adverse consequences. Secondly, although RSV effectively inhibited chronic stress–induced excitation of RVLM neurons in rats, it remains unclear whether this effect will translate to other brain regions or humans. Additionally, our study focused solely on young rats (8 weeks old), while age-related increases in oxidative stress are well documented (59). Expanding our research to include diverse age groups will provide a more comprehensive understanding of RSV's impact on SIH across the lifespan. Finally, while we demonstrated that RSV promotes Sirt3 expression through AMPK activation to inhibit SIH development, further exploration is needed to elucidate whether other pathways contribute to RSV's antihypertensive effects.

## Experimental procedures

### Materials and antibodies

Plasma NE ELISA kit was purchased from FineTest. Mitochondria/cytosol fractionation kit (abs9344) was purchased from Absin. Restore Western blot stripping (21059) was purchased from Thermo Fisher Scientific. TransZol Up (ET111-01) was purchased from TransGen. Hifair II 1st Strand cDNA Synthesis Kit (11119ES60), Hieff qPCR SYBR Green Master Mix (11203ES08), DAPI Fluoromount-G (36308ES11), and Annexin V-FITC/PI apoptosis detection kit (40302ES60) were purchased from Yeasen. Bicinchoninic acid protein assay kit, DHE (S0063), dichlorofluorescein (DCF, S0033M-1), total SOD assay kit with WST-8 (S0101S), CAT activity assay kit (S0051), MMP assay kit (C2006), caspase 3 activity assay kit (C1116), and mitochondrial superoxide assay kit with MitoSOX Red (S0061S) were purchased from Beyotime. Lipid peroxidation MDA assay kit (A003-1-2) was purchased from Nanjing Jiancheng. CCK-8 assay (B34302) was purchased from Bimake. Lipofectamine 2000 transfection reagent (11668500) was purchased from Invitrogen. Sirt3 shRNA lentivirus and NC shRNA lentivirus were obtained from GenePharma. An adeno-associated virus (AAVs) containing MitoEGFPmCherry, Sirt3 siRNA, and scrambled siRNA were carried out at Hanbio. RSV (HY-16561) and Com.C (HY-13418A) were offered by MedChemExpress.

Antibodies used for immunofluorescence and Western blot were as follows: c-Fos (#2250, CST), TH (sc-25269, Santa Cruz), caspase 3 (sc-56053, Santa Cruz), NeuN (ab177487, Abcam), NeuN (ab104224, Abcam), p-AMPK (AF5908, Beyotime), Sirt3 (#2627, CST), goat anti-rabbit AF594 (111-585-003, Jackson ImmunoResearch), goat-anti mouse FITC (115-095-003, Jackson ImmunoResearch), PGC1α (A19674, ABclonal), TFAM (A3173, ABclonal), ATP5A (GB113455, Servicebio), UQCRC2 (sc-390378, Santa Cruz), SDHB (AG3213, Beyotime), DRP1 (ab184247, Abcam), MFN2 (340604, ZenBio), PINK1 (AF7755, Beyotime), Parkin (ET1702-60, Huabio), cytochrome C (AF2047, Beyotime), GPX4 (A11243, ABclonal), FTH1 (A19544, ABclonal), ACSL4 (A20414, ABclonal), AMPK (AF1627, Beyotime), Ac-SOD2 (ab137037, Abcam), SOD2 (GB111875-100, Servicebio), and β-actin (HRP-60008, Proteintech).

### Animals

Two hundred and sixty 8-week-old male and female Sprague–Dawley rats, weighing between 250*g* and 300*g*, were obtained from the Animal Resources Center, Shanghai Medical College of Fudan University and housed in a specified pathogen-free facility with a 12-h light/dark cycle. The room temperature was 23 ± 1 °C and the humidity was controlled. Water and food are provided regularly. Our experimental procedures were conducted in conformity with the Animal Experimental Research Center of Shanghai University (Permit No. SYXK (HU) 2019-0020) and also following the Guidelines for the Care and Use of Experimental Animals. The establishment of the SIH rat model was informed by our past investigations (60). In brief, we put the rats in the stressed groups into an electrical cage with a grid floor. The intermittent electrical shocks about 35 to 80 V were applied for a duration ranging from 50 to 100 ms every 2 to 30 s randomly. The buzzer was used to produce the noises (88–98 dB) as a conditioned stimulus. The stressors outlined above were applied to the rats for 2 h twice daily, persisting for half a month. For control groups, the rats were put in the same conditions without foot shocks and noises. Fig. S10 showed the general study design. No inclusion and exclusion criteria were predetermined. Male and female rats were used randomly and no major sex-specific differences were observed.

### Isolation of primary neurons and treatment

RVLM tissues from Sprague–Dawley rats aged less than 24 h were isolated using the punch-out technique (42, 61), based on established research methods and with guidance from the standard rat atlas. Tissue samples were treated with 0.125% trypsin (containing 100 U DNase I) for 20 min at room temperature, followed by centrifugation at 1000 rpm for 10 min. The supernatant was decanted, and the resulting pellets were suspended in DMEM/F12 medium enriched with 10% FBS, 1% penicillin-streptomycin, and 1% L-glutamine. After the initial plating period of 6 h, the medium was replaced with a new neurobasal medium supplemented with 2% B27, 1% penicillin–streptomycin, and 1% l-glutamine. The cells were then cultured at 37 °C in a 5% CO_2_-humidified environment for 7 to 10 days. To evaluate the inhibitory effect of RSV on mitochondrial oxidative stress–induced apoptosis and ferroptosis, RVLM primary neurons were preliminarily treated with 25, 50, or 100 μM of RSV for 4 h and then subjected to 100 μM of H_2_O_2_ for 12 h, in accordance with a previous study with minor modifications (62).

### N2a cell culture and treatment

The mouse neuroblastoma N2a cells were purchased from ATCC and cultured in Dulbecco’s modified Eagle’s medium containing 10% fetal bovine serum at 37 °C in a 5% CO_2_-humidified environment. The cells were identified by short tandem repeat profiling and were *mycoplasma* negative. An appropriate concentration of RSV was selected for AMPK/Sirt3 pathway activation. N2a cells were treated with vehicle or various doses of RSV (25, 50, and 100 μM) for 4 h. Com.C-treated cells were pre-incubated with Com.C (10 and 20 μM) for 2 h before RSV (50 μM) supplement for another 4 h. Then, 50 μM RSV and 20 μM Com.C were selected for the subsequent experiments. The N2a cell oxidative stress model was constructed using 100 μM H_2_O_2_ for 24 h. The *in vitro* study using N2a cells was divided into two experiments. Experiment 1 comprised three groups. The cells in the first group were pretreated with the vehicle without H_2_O_2_ treatment (control group), those in the second group were pretreated with the vehicle for 4 h and then exposed to H_2_O_2_ (100 μM, 24 h, H_2_O_2_ group), and those in the third group were pretreated with RSV (50 μΜ, 4 h) and then exposed to H_2_O_2_ (H_2_O_2_ plus RSV group). Experiment 2 was divided into five groups: control, H_2_O_2_, H_2_O_2_ plus RSV, H_2_O_2_ plus RSV plus Com.C, and H_2_O_2_ plus RSV plus Sirt3 siRNA groups. The cells in the control, H_2_O_2_, H_2_O_2_ plus RSV, and H_2_O_2_ plus RSV plus Com.C groups were transfected with scrambled siRNA. The cells in the H_2_O_2_ plus RSV plus Sirt3 siRNA group were transfected with Sirt3 siRNA. The incubation time and doses used were selected according to previous studies and manufacturers’ instructions (63, 64, 65).

### Assessments of BP and HR

A femoral artery cannulation method was employed to record the BP and HR in anesthetized rats as we reported before (19). The rats were placed dorsally on a temperature-controlled pad in an anesthetized state. Here, the inhaled isoflurane (1.5–3%) was used. Using blunt dissection, the right femoral artery and vein of the rats were isolated. After the placement of a polyethylene catheter into the right femoral artery, a pressure transducer was connected to transmit the BP signals to a PowerLab system (AD Instruments). The computer calculated HR by analyzing the phasic arterial BP wave automatically.

### Monitoring of RSNA

Recording of RSNA was conducted as we tested before (19). Each rat was anesthetized with inhaled isoflurane as mentioned above. Through left retroperitoneal dissection, the left renal sympathetic nerve was exposed and attached to platinum/iridium recording electrodes. Subsequently, the silicone gel was used to encapsulate the electrode–nerve complex, separating the electrodes and the sympathetic nerve from surrounding tissues and preventing desiccation. The nerve activity was amplified (×1000) and filtered (bandpass 100–3000 Hz). Using the PowerLab system, the RSNA signal was collected. The maximum RSNA value was assessed in rats euthanized using narcotic overdose (200 mg kg^−1^ pentobarbital, i.v.). The period of 20 to 30 min post-rat demise was dedicated to capturing the background noise. The baseline RSNA level was calculated as a ratio to the maximum RSNA after the elimination of background noise.

### Detection of plasma NE

In an anesthetized state, we collected the blood samples by cardiac puncture and drained them into precooling anticoagulant tubes. The supernatants were obtained through centrifugation at 4 °C for 15 min at 1000*g* for detection. The plasma NE level was detected *via* ELISA. According to the instructions with the ELISA kit, the OD value was tested at 450 nm using a microplate reader (Molecular Devices).

### Transmission electron microscopy

The electron microscopy procedures were conducted according to our previous publication (18). Brain tissues containing the RVLM were dissected into specimens at a thickness of 1 mm. Subsequently, the tissues and the harvested RVLM primary neuronal cells were immersed in the fixative solution at 4 °C overnight. Then, a 2-h exposure of 1% osmium tetroxide was applied to these specimens, followed by gradient dehydration using ethanol, and embedding in fresh epoxy resin. Finally, these samples underwent slicing using an ultramicrotome and then were treated with a mixture of 2% uranyl acetate and lead citrate before being analyzed through a Hitachi HT7800 TEM (Hitachi). Quantification of mitochondrial number and length in neurons, as well as the count of mitochondrial cristae, was performed *via* ImageJ software.

### Immunofluorescence

Rats under pentobarbital sodium anesthesia (50 mg/kg, i.p.) were perfused with a heparinized saline solution followed by a paraformaldehyde solution. The rat brain tissues were exposed to 4% paraformaldehyde for postfixation lasting 12 h. Afterward, they underwent dehydration in 20% and 30% sucrose solutions at 4 °C until they reached the bottom. A cryostat (HM525, Microm) facilitated the slicing of frozen coronal slices at a thickness of 30 μm, encompassing the RVLM. Following the RVLM coronal slices preparation, permeabilization was achieved with 0.3% Triton X-100 for 30 min, followed by blocking nonspecific binding using 5% goat serum for 1 h at room temperature. The sections were subsequently incubated with primary antibodies, including TH, c-Fos, NeuN, Caspase 3, p-AMPK, and Sirt3, overnight at 4 °C. After the sections had been washed, they underwent incubation with goat anti-rabbit AF594 and goat anti-mouse FITC secondary antibodies for 2 h at 37 °C. The collection of fluorescent images was carried out using a confocal laser scanning microscope (LSM880, ZEISS).

### Image quantification procedures

A confocal laser scanning microscope (LSM880, ZEISS) at ×40 magnification was utilized to acquire a minimum of 12 images, creating confocal Z-stack images. To assess the volume of mCherry-ONLY signals, the confocal images were converted into 3D reconstruction files using Imaris 9.7 software. The numbers of EGFP puncta and mCherry puncta were quantified using the spots module in Imaris 9.7. The mCherry-ONLY signal (mCherry-positive/EGFP-negative) was determined by subtracting the number of EGFP puncta from the total mCherry puncta. Additional images were processed and analyzed using Image J software.

### Western blot

We isolated the total protein using a lysis buffer. Cytosol can be extracted using a mitochondria/cytosol fractionation kit. The sediment was removed after centrifugation at 12,000 rpm for 15 min and the supernatant was collected. The protein concentration was determined by using the bicinchoninic acid protein assay kit. The separation of protein samples was carried out through 4%–20% SDS-PAGE, with subsequent transfer to polyvinylidene fluoride membranes for analysis. Treatment with a blocking buffer (0.05% Tween 20 and 5% nonfat milk) at room temperature lasted for 2 h on the membranes. After that, they underwent primary antibody incubation at 4 °C overnight and secondary antibody treatment at 37 °C for 1 h. For close bands, we used Restore Western blot stripping to remove the previous antibodies for half an hour. Then, different primary antibodies were applied to the membranes. Finally, the detection of immunoreactive bands was achieved by utilizing a super ECL reagent and an automatic analysis system. The quantitative analysis of gray values was performed using Image J software. The levels of phosphorylated or acetylated proteins were calculated relative to the corresponding total protein levels, while the levels of the remaining proteins are relative to those of β-actin. The primary antibodies included PGC1α, TFAM, ATP5A, UQCRC2, SDHB, cytochrome C, GPX4, FTH1, ACSL4, DRP1, MFN2, PINK1, Parkin, Ac-SOD2, SOD2, p-AMPK, AMPK, Sirt3, and β-actin, and the secondary antibodies included goat anti-mouse IgG and goat anti-rabbit IgG.

### Quantitative real-time PCR

The transZol Up was employed for the extraction of total RNA from RVLM tissues, N2a cells, or primary RVLM neurons according to the manufacturer’s guidelines. complementary DNA (cDNA) synthesis was performed using 1 μg of RNA with a Hifair II 1st Strand cDNA Synthesis Kit. By employing the Hieff qPCR SYBR Green Master Mix and cDNA in combination with gene-specific primers (Table S1), qRT-PCR analysis was conducted. The level of relative gene expression was normalized to *Gapdh*.

### RVLM microinjection

Consistent with earlier publications, RVLM microinjection protocols were implemented (18). After being anesthetized, the rats were positioned face-down. Their heads were secured to a stereotaxic apparatus. The skull was visible under the midline scalp incision and was further adjusted to the same level. AAVs: MitoEGFPmCherry served for monitoring mitochondrial degradation, with EGFP and mCherry fluorophores targeting mitochondria through fusion with 29 amino acids of human cytochrome C oxidase subunit 8 (38). RSV was dissolved in a vehicle (0.05% DMSO plus saline) and used at concentrations of 80 and 160 μM. Com.C was dissolved in a vehicle and used at concentrations of 100 nM. These doses were based on preliminary experiments and previous studies (66, 67, 68). Using a specific glass micropipette, RSV, Com.C, AAVs:MitoEGFPmCherry, Sirt3 shRNA lentivirus, or NC shRNA lentivirus was administered through bilateral microinjections into the RVLM (caudal to lambdoid suture, 3.7–4.0 mm; lateral to the midline, 2 mm; and ventral to the dura, 8.0 mm) at 50 nl/side according to the standard rat atlas of Paxinos and Watson (61). Fig. S10 illustrated the specific time points at which the microinjections were administered. To confirm the injection site, the RVLM underwent microinjection, with 50 nl of 2% pontamine sky blue being administered (Fig. S11). The incision was cleaned and sutured after microinjection.

### Measurement of intracellular ROS levels

The ROS generation *in vivo* was measured by DHE staining. After incubation with 5 μM DHE at 37 °C for half an hour, the RVLM sections were washed with PBS. The confocal laser scanning microscope was utilized for the detection of fluorescence signals. The ROS levels *in vitro* were determined by measuring DCF fluorescence with flow cytometry. Equal cell numbers were stained with DCF. All the data were analyzed by FlowJo software for geometric mean fluorescence intensity.

### Measurement of mitochondrial ROS levels

RVLM primary neurons, after varied treatments, were incubated with 10 μM MitoSOX for 30 min to analyze mitochondrial ROS levels. The cells were then triple-washed with warm PBS, fixed with DAPI Fluoromount-G, and observed utilizing the confocal laser scanning microscope.

### Measurements of SOD activity, CAT activity, and MDA level

The SOD activity, CAT activity, and MDA content in RVLM tissues and N2a cells were detected using corresponding kits in accordance with the manufacturer’s instructions. The absorbance was assessed at 450 (SOD), 520 (CAT), and 530 (MDA) nm using a SpectraMax iD5 microplate reader.

### Caspase 3 activity detection

Assessment of caspase 3 activity was performed using a colorimetric assay kit. Following treatment, RVLM primary neurons were harvested, lysed, and centrifuged to obtain the lysate supernatant. The caspase 3 activity in the supernatant samples was then evaluated adhering to the manufacturer’s protocols. Subsequently, normalization of caspase 3 activity and determination of total protein in each group were carried out by measuring the optical density at a wavelength of 405 nm.

### CCK-8 assay

Cell viability was determined through a CCK-8 assay. Briefly, N2a or RVLM primary neuronal cells (1 × 10^4^ cells/well) were placed in 96-well plates and cultured for 24 h. Subsequently, the N2a cells were exposed to various doses (0, 25, 50, 100, 200, and 400 μM) of H_2_O_2_ for another 24 h. The RVLM primary neuronal cells were exposed to various doses (0, 25, 50, 100, and 200 μM) of H_2_O_2_ for another 12 h. Following this step, a newly prepared medium with 10% CCK-8 reagent was added to replace the original medium in all wells. The cells were incubated for half an hour. The optical density was read with a SpectraMax iD5 microplate reader at 450 nm.

### Cell transfection

N2a cells were grown in 6-well or 12-well plates and were transfected with *Sirt3* siRNA or scrambled siRNA at 80% confluence for 2 days according to the manufacturer’s guidelines. The siRNA oligonucleotide sequences are shown as follows: *Sirt3* siRNA 5′-GGAUGGACAGGACAGAUAATT-3′ and 5′-UUAUCUGUCCUGUCCAUCCTT-3′.

### Apoptosis detection assay

The apoptosis of RVLM primary neurons was assessed using the Annexin V-FITC/PI apoptosis assay kit according to the manufacturer’s instructions. Briefly, Annexin V-FITC and PI were added to cells suspended in 1× binding buffer. The mixture was incubated for 15 min in the dark at room temperature and analyzed using a flow cytometer within 1 h.

### Detection of MMP

For RVLM primary neurons, MMP was analyzed by monitoring TMRE fluorescence intensity using a SpectraMax iD5 microplate reader. For N2a cells, a commercial kit was used to determine the MMP level. Briefly, after different treatments, the cells were mixed with 500 μl of JC-1 working stock solution and incubated at 37 °C for 20 min. Then, the cells underwent two washes with a JC-1 buffer solution. A laser scanning confocal microscope was used to test the fluorescence intensity. The red/green fluorescence emission intensity ratio quantifies the MMP.

### Statistical analysis

All data were represented by the mean ± SD, and statistical analysis was executed using Prism 9.1 software. Nonparametric tests were utilized for group comparisons when n ≤ 6, while the Shapiro–Wilk test was employed for normality distribution verification when n > 6. Parametric tests were employed for group comparison if the data were normally distributed; otherwise, nonparametric tests were utilized. Significant differences between two groups were assessed with the unpaired two-tailed Student’s *t* test for parametric data and the Mann-Whitney U test for nonparametric data. For comparisons with multiple groups, parametric data underwent analysis with one-way ANOVA and *post hoc* Bonferroni test, while nonparametric data were evaluated using the Kruskal–Wallis test and *post hoc* Dunn test. A *p* value less than 0.05 denoted statistical significance.

## Data availability

All data that support the findings in this study are available from the corresponding authors upon reasonable request.

## Supporting information

This article contains supporting information.

## Conflict of interest

The authors declare that they have no conflicts of interest with the contents of this article.

## Supporting information

Supporting Information

Jbc-checklist

Full unedited blot
