# Supplementary material for: Resveratrol reduces RVLM neuron activity via activating the AMPK/Sirt3 pathway in stress-induced hypertension
Source: J Biol Chem. 2025 Mar 10;301(4):108394. doi: 10.1016/j.jbc.2025.108394 (PMC12002922; doi:10.1016/j.jbc.2025.108394)
Supplement: Supplementary Table 1 [file mmc1.docx]

**Supplementary Table 1** Primers used for quantitative real-time PCR (qRT-PCR)

| Gene | Primer (5’- 3’) |
| --- | --- |
| *Gpx4* | F: ATGAAAGTCCAGCCCAAGG  R: GGTCCTTCTCTATCACCTGGG |
| *Fth1* | F: AACCTGGAGTTGTATGCCTC  R: TTTCTCAGCATGTTCCCTCTC |
| *Acsl4* | F: TTGGCTACTTACCTTTGGCTC  R: AATCACCCTTGCTTCCCTTC |
| *Sirt3* | F: CTACATGCACGGTCTGTCGAA  R: GCCAAAGCGAAGTCAGCCATA |
| *Gapdh* | F: GTCGGTGTGAACGGATTTG  R: TCCCATTCTCAGCCTTGAC |
