# Supplementary material for: Resveratrol reduces RVLM neuron activity via activating the AMPK/Sirt3 pathway in stress-induced hypertension
Source: J Biol Chem. 2025 Mar 10;301(4):108394. doi: 10.1016/j.jbc.2025.108394 (PMC12002922; doi:10.1016/j.jbc.2025.108394)
Supplement: Supporting Information [file mmc2.pdf]

Supporting Information

Title: Resveratrol reduces RVLM neuron activity via activating the AMPK/Sirt3 pathway in stress-induced hypertension

Authors: Lin-Ping Wang[1,2,‡], Tian-Feng Liu[1,2,‡], Teng-Teng Dai[2,‡], Xin Deng[3,‡], Lei Tong[2], Qiang-Cheng Zeng[4], Qing He[4], Zhang-Yan Ren[2], Hai-Li Zhang[5], Hai-Sheng Liu[5], Yan-Fang Li[6], Wen-Zhi Li[7,*], Shuai Zhang[8,*], and Dong-Shu Du[1,2,4,5,6,*]

[1]*School of Environmental and Chemical Engineering, and* [2]*School of Life Sciences, Shanghai University, Shanghai, China;* [3]*Shanghai Institute of Cardiovascular Diseases, Zhongshan Hospital, Institute of Biomedical Science, Fudan University, Shanghai, China;* [4]*College of Life Sciences, Dezhou University, Dezhou, Shandong, China;* [5]*College of Agriculture and Bioengineering, Heze University, Heze, Shandong, China;* [6]*Department of Preventive Medicine, Heze Medical College, Heze, Shandong, China;* [7]*Department of Urology, School of Medicine, Shanghai General Hospital, Shanghai Jiao Tong University, Shanghai, China; and* [8]*International Cooperation Laboratory of Molecular Medicine, Academy of Chinese Medical Sciences, Zhejiang Chinese Medical University, Hangzhou, Zhejiang, China.*

*Corresponding authors: wenchihlee@sina.com (WZL), szhang@zcmu.edu.cn (SZ), and dsdu@shu.edu.cn (DSD).

‡These authors contributed equally to this work.

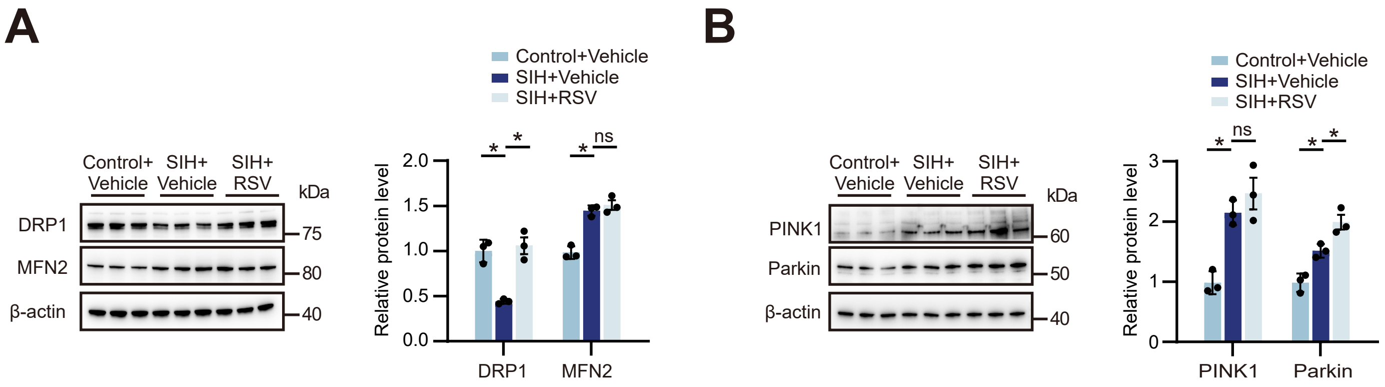

Supplementary Figure 1. Chronic stress reduced mitochondrial fission and promoted fusion in the RVLM of rats. (*A*) The protein levels of mitochondrial fission/fusion protein DRP1 and MFN2 were measured by western blot. n = 3 rats per group. (*B*) The protein levels of mitophagy-related protein PINK1 and Parkin were analyzed by western blot. n = 3 rats per group. Data are presented as mean ± SD. Significance was evaluated using the Kruskal-Wallis test and post hoc Dunn test. n.s., no significance. *$p < 0.05$. RVLM, rostral ventrolateral medulla; SD, standard deviation.

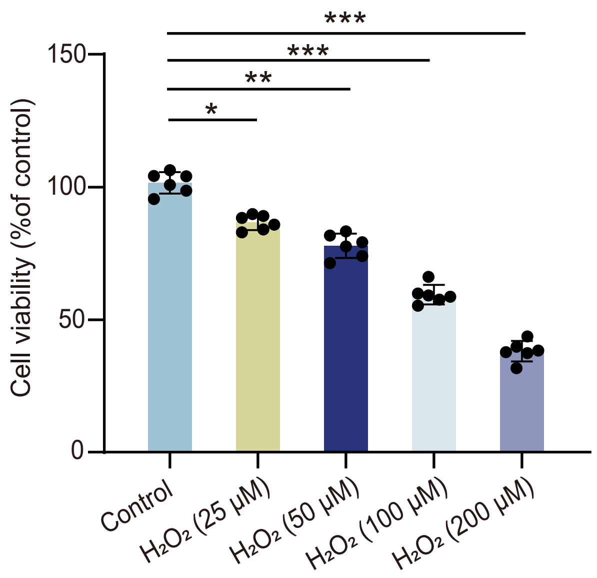

Supplementary Figure 2. Cell viability was determined using the cell counting kit-8 assay after treatment with different concentrations of H_2O_2 (25, 50, 100, and 200 μM) in the RVLM primary neurons. n = 6 of independent cell culture preparations. Data are presented as mean ± SD. Significance was evaluated using the Kruskal-Wallis test and post hoc Dunn test.*$p < 0.05$, **$p < 0.01$, ***$p < 0.001$. RVLM, rostral ventrolateral medulla; SD, standard deviation.

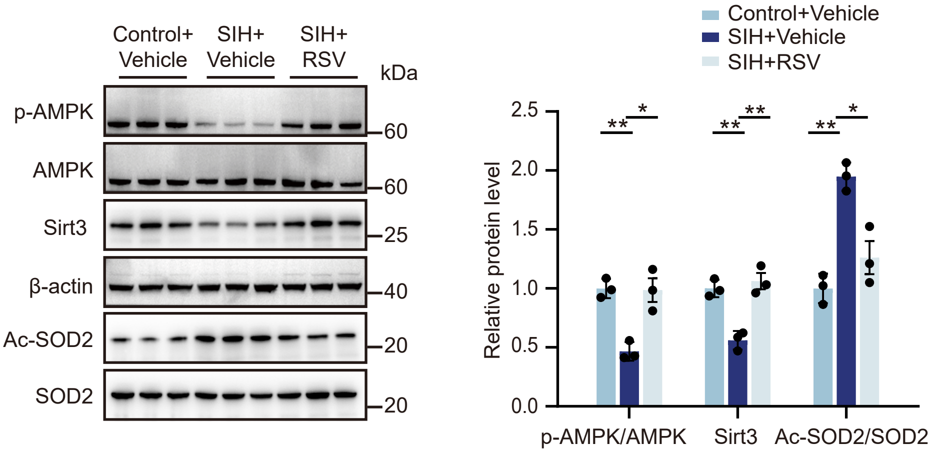

Supplementary Figure 3. Expression levels of p-AMPK/AMPK, Sirt3, and Ac-SOD2/SOD2 in the RVLM of SIH rats receiving corresponding treatment. n = 3 rats per group. Data are presented as mean ± SD. Significance was evaluated using the Kruskal-Wallis test and post hoc Dunn test. *$p < 0.05$, **$p < 0.01$. RVLM, rostral ventrolateral medulla; SD, standard deviation; SIH, stress-induced hypertension.

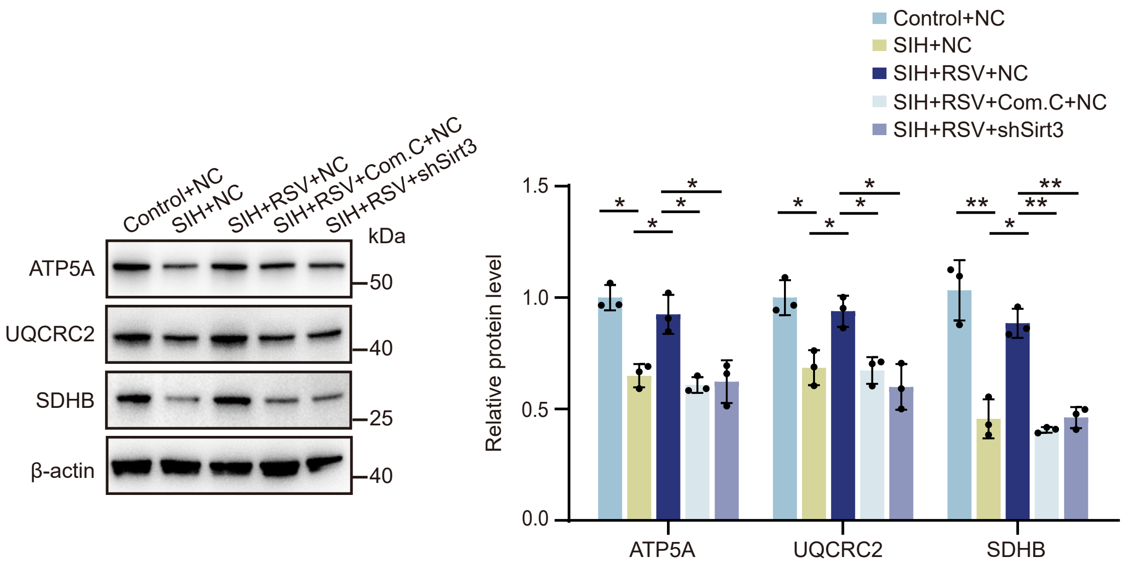

Supplementary Figure 4. The protein expressions of mitochondrial subunits including ATP5A, UQCRC2, and SDHB were measured by western blot. n = 3 rats per group. Data are presented as mean ± SD. Significance was evaluated using the Kruskal-Wallis test and post hoc Dunn test. *p < 0.05, **p < 0.01. SD, standard deviation;

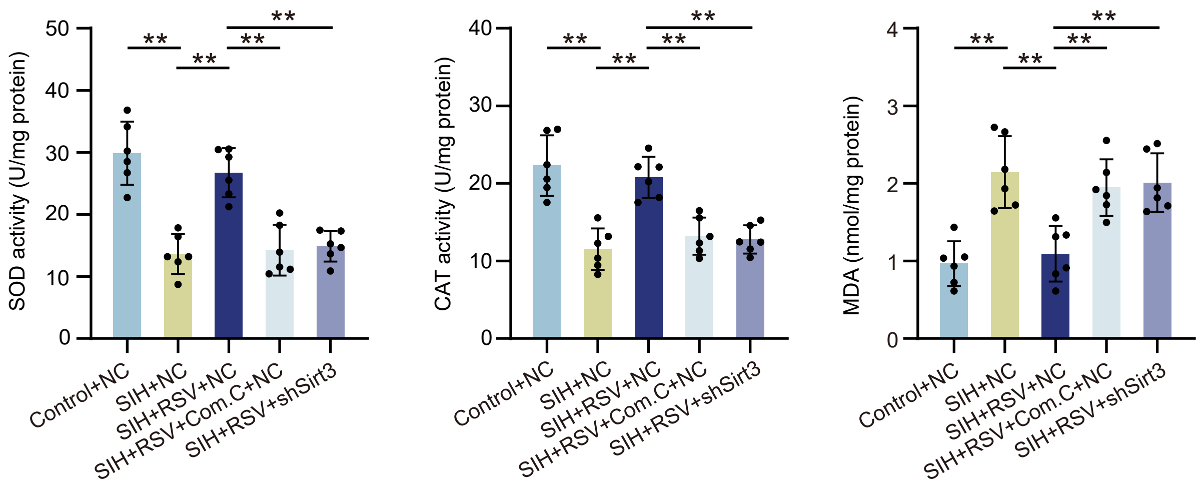

Supplementary Figure 5. The activities of SOD and CAT and the MDA were detected by corresponding kits. n = 6 rats per group. Data are presented as mean ± SD. Significance was evaluated using the Kruskal-Wallis test and post hoc Dunn test. ***$p < 0.01$. CAT, catalase; MDA, malondialdehyde; SD, standard deviation; SOD, superoxide dismutase.

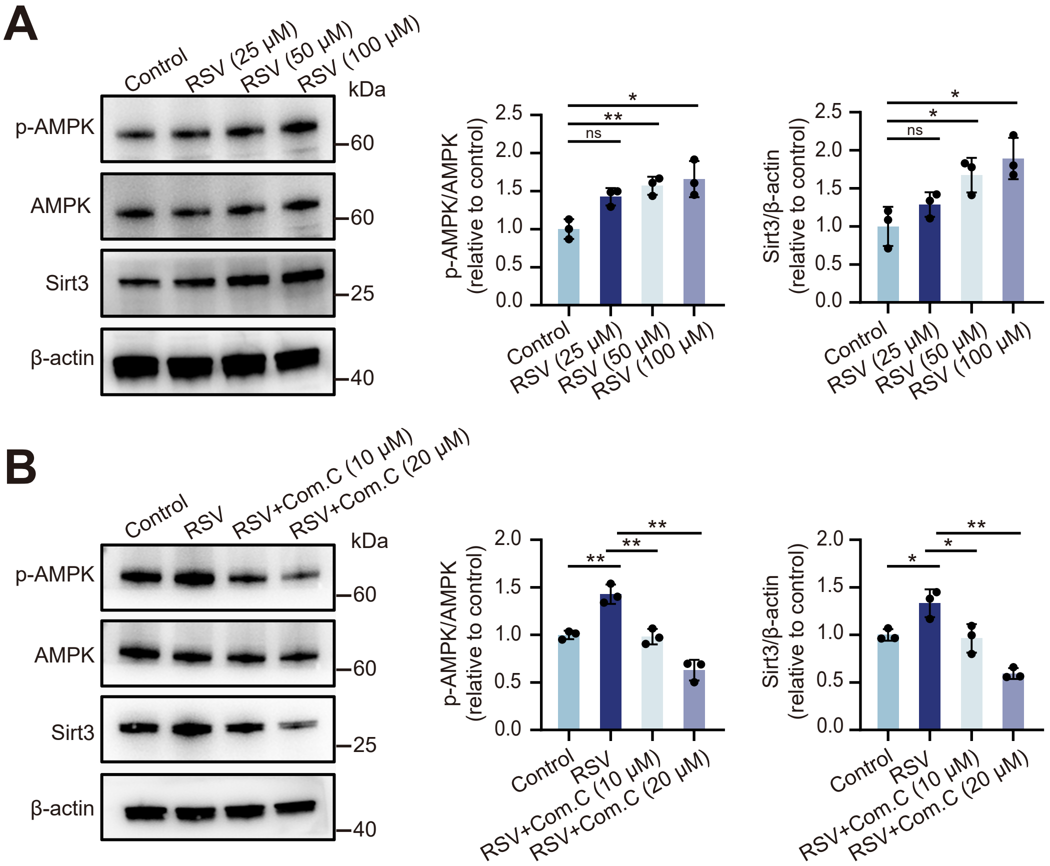

Supplementary Figure 6. RSV and/or Com.C treatment changes the p-AMPK/AMPK ratio and Sirt3 protein levels. The protein expressions of p-AMPK and Sirt3 with various concentrations of RSV treatment (*A*) or combined RSV and Com.C treatement (*B*) were analyzed by Western blot. n = 3 of independent cell culture preparations. Data are presented as mean ± SD. Significance was evaluated using the Kruskal-Wallis test and post hoc Dunn test. n.s., no significance. *$p < 0.05$, **$p < 0.01$. Com.C, Compound C; RSV, resveratrol; SD, standard deviation.

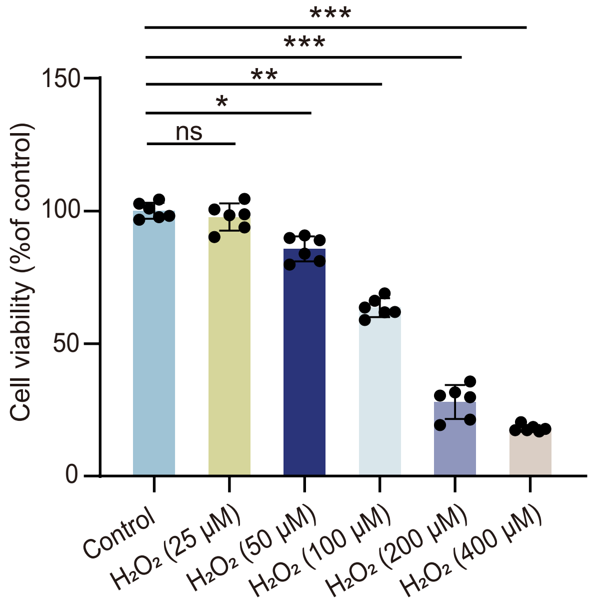

Supplementary Figure 7. Cell viability was determined using the cell counting kit-8 assay after treatment with different concentrations of H_2O_2 (25, 50, 100, 200, and 400 μM) in N2a cells. n = 6 of independent cell culture preparations. Data are presented as mean ± SD. Significance was evaluated using the Kruskal-Wallis test and post hoc Dunn test. n.s., no significance. $*p < 0.05$, $**p < 0.01$, $***p < 0.001$. SD, standard deviation.

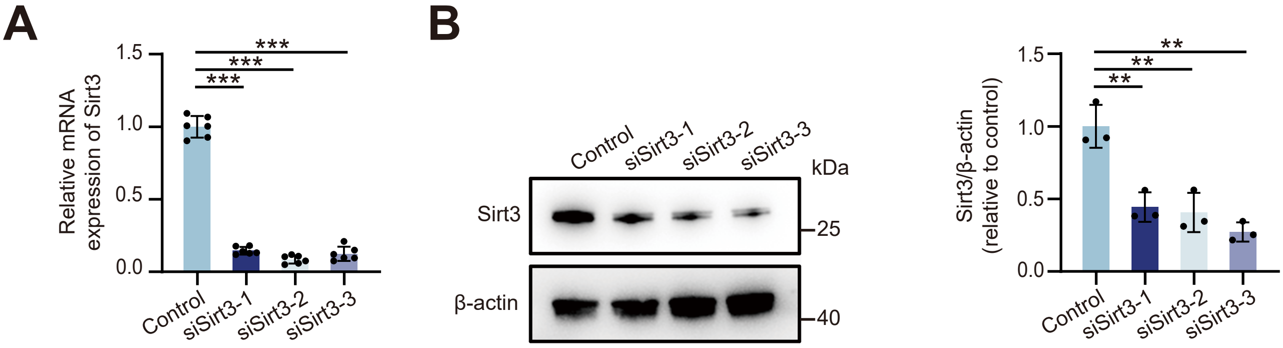

Supplementary Figure 8. qRT-PCR (*A*) and Western blot (*B*) were performed to evaluate the efficiency of interference sequences of *Sirt3* in N2a cells. n = 6 (mRNA levels) or 3 (protein levels) of independent cell culture preparations. Data are presented as mean ± SD. Significance was evaluated using the Kruskal-Wallis test and post hoc Dunn test. $^{**}p < 0.01$, $^{***}p < 0.001$. qRT-PCR, quantitative real-time PCR; SD, standard deviation.

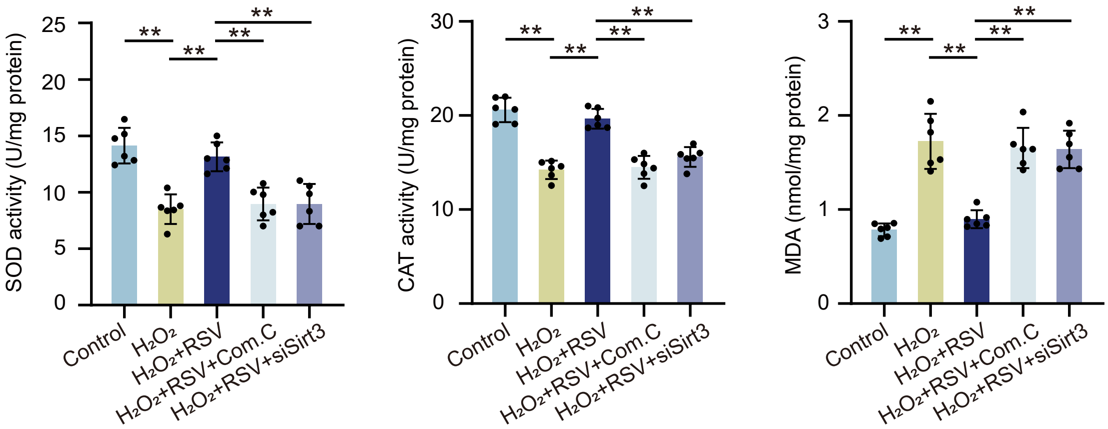

Supplementary Figure 9. The activities of SOD and CAT and the content of MDA were detected by corresponding kits in N2a cells. n = 6 of independent cell culture preparations. Data are presented as mean ± SD. Significance was evaluated using the Kruskal-Wallis test and post hoc Dunn test. $^{**}p < 0.01$. CAT, catalase; MDA, malondialdehyde; SD, standard deviation; SOD, superoxide dismutase.

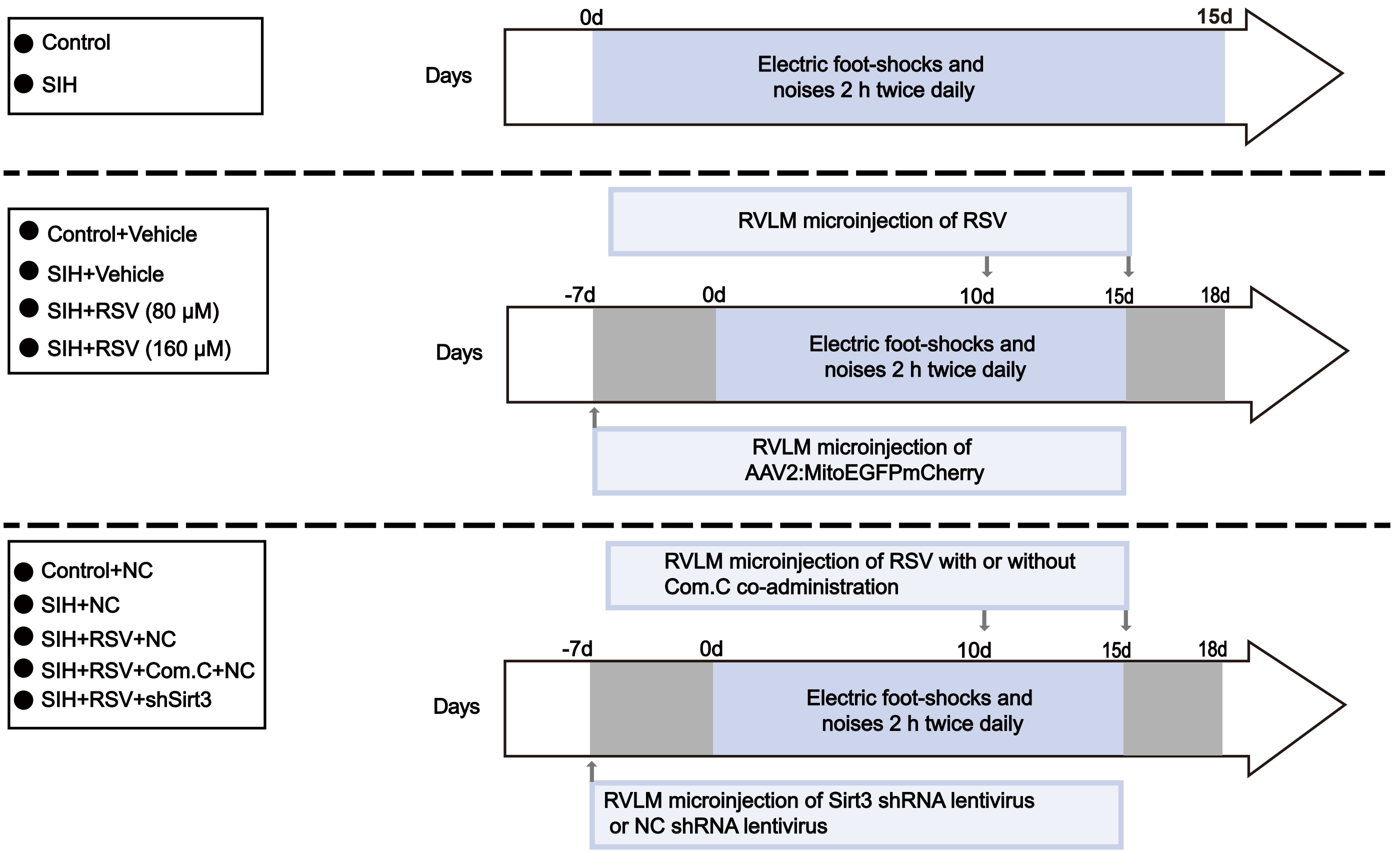

Supplementary Figure 10. Animal experiment timeline. Com.C, compound C; RSV, resveratrol; RVLM, rostral ventrolateral medulla; SIH, stress-induced hypertension.

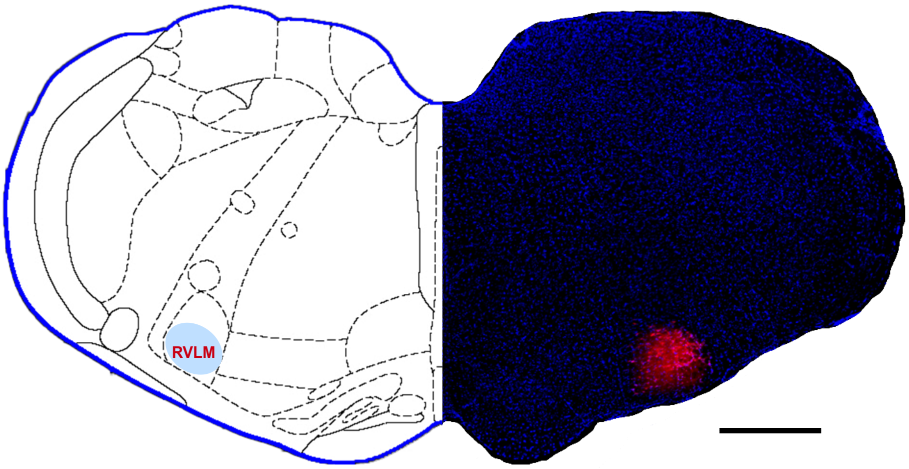

Supplementary Figure 11. Photomicrograph showed diagram of RVLM area by digital Paxino's atlas (left side) and localization of RVLM site (right side). Scale bar = 500 µm. RVLM, rostral ventrolateral medulla.
