# Supplementary material for: Resveratrol reduces RVLM neuron activity via activating the AMPK/Sirt3 pathway in stress-induced hypertension
Source: J Biol Chem. 2025 Mar 10;301(4):108394. doi: 10.1016/j.jbc.2025.108394 (PMC12002922; doi:10.1016/j.jbc.2025.108394)
Supplement: Full unedited blot [file mmc4.pdf]

Full unedited blot

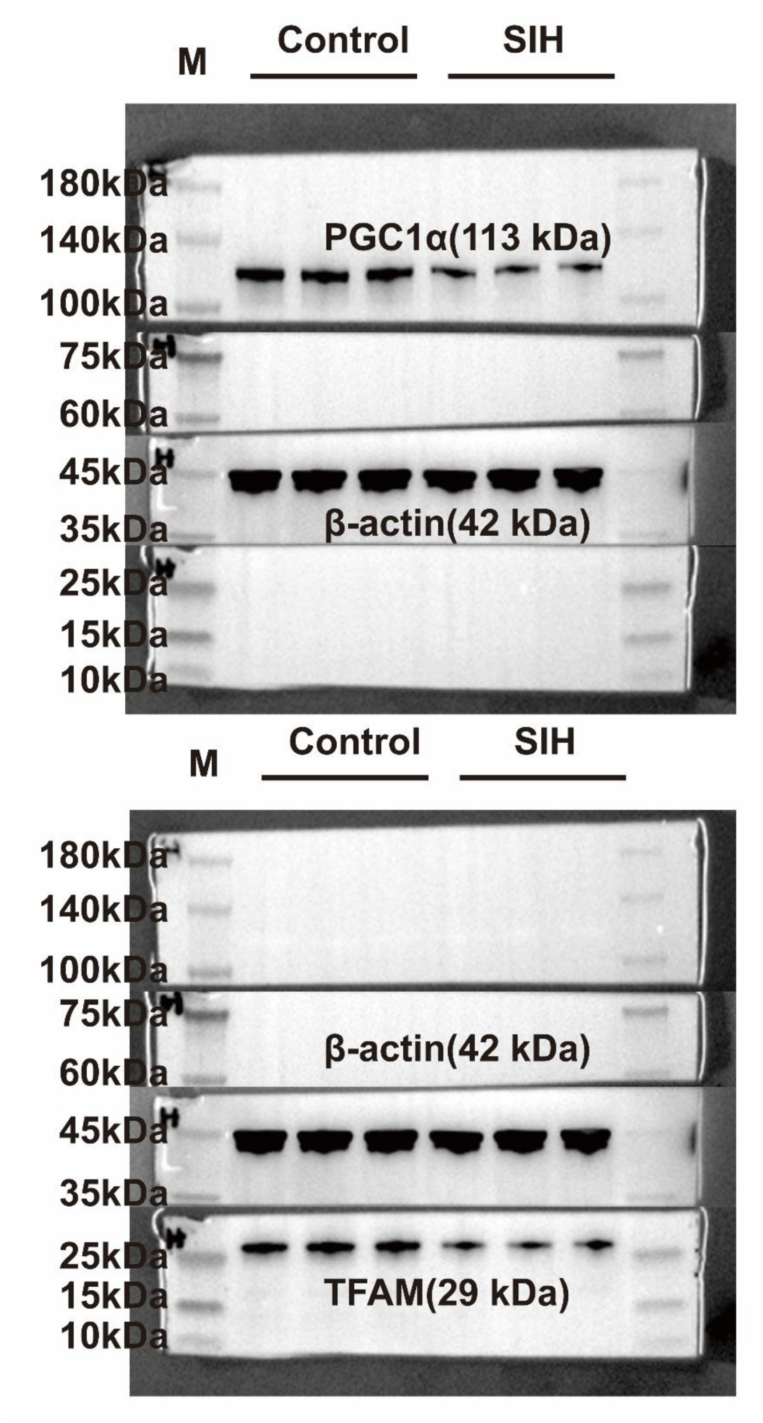

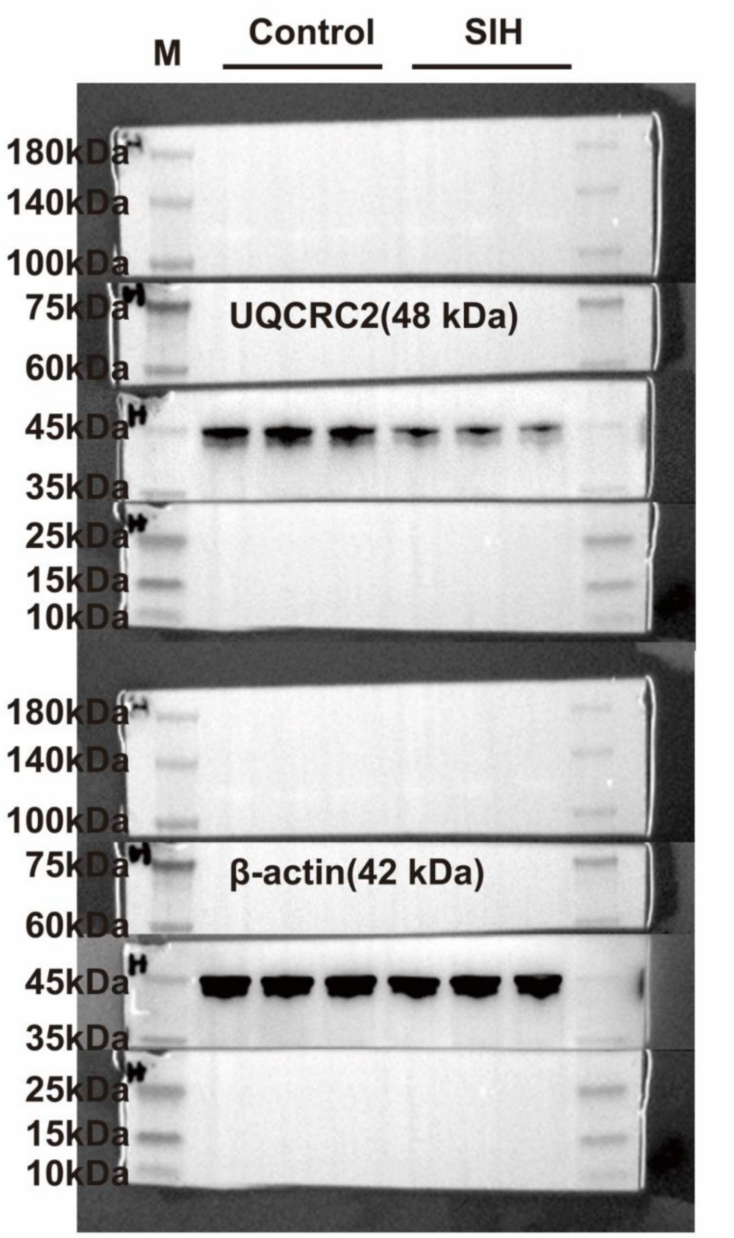
M
Control
SIH
180kDa
140kDa
100kDa
75kDa
UQCRC2(48 kDa)
60kDa
45kDa
35kDa
25kDa
15kDa
10kDa
180kDa
140kDa
100kDa
75kDa
β-actin(42 kDa)
60kDa
45kDa
35kDa
25kDa
15kDa
10kDa

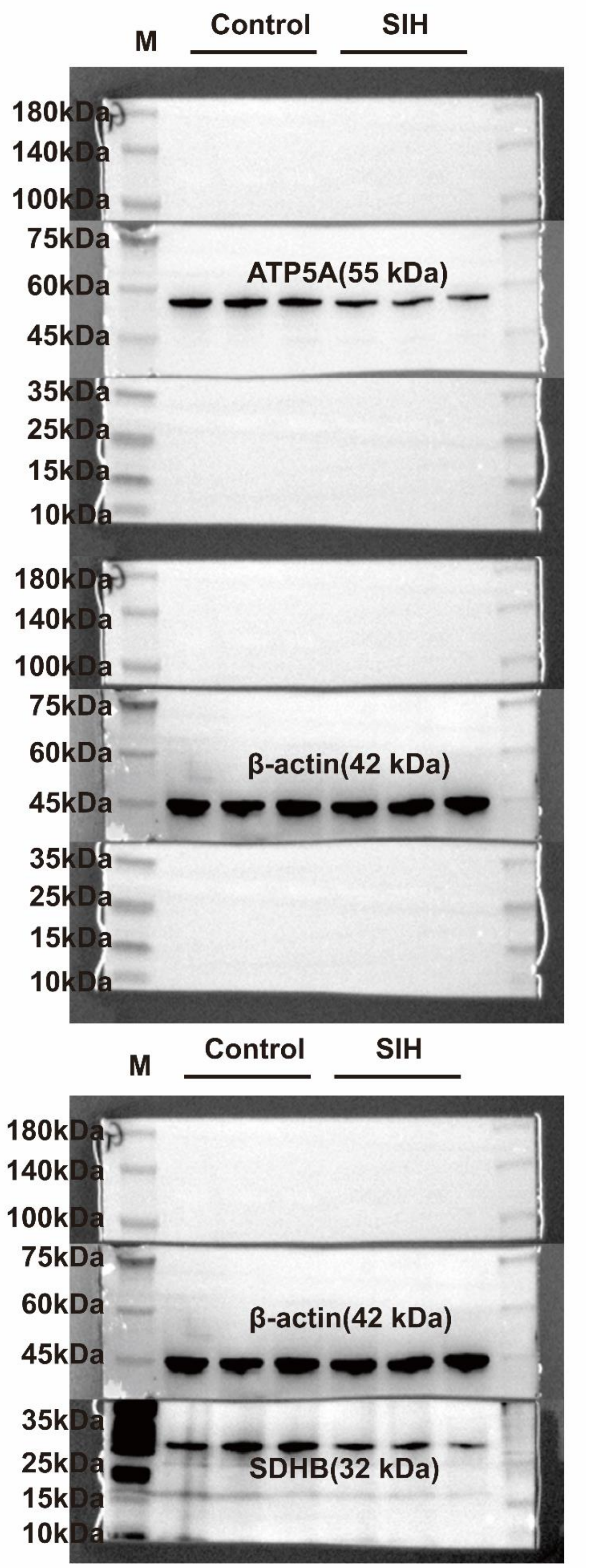

Full unedited blot for Figure 1B. M, marker; SIH, stress-induced hypertension.

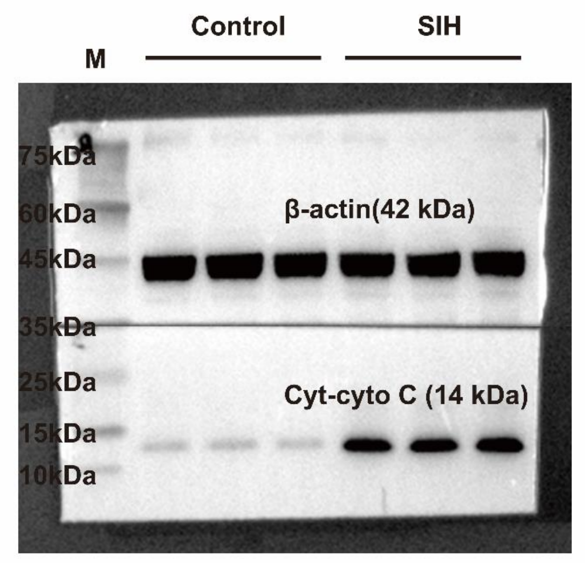

Full unedited blot for Figure 1E. M, marker; SIH, stress-induced hypertension.

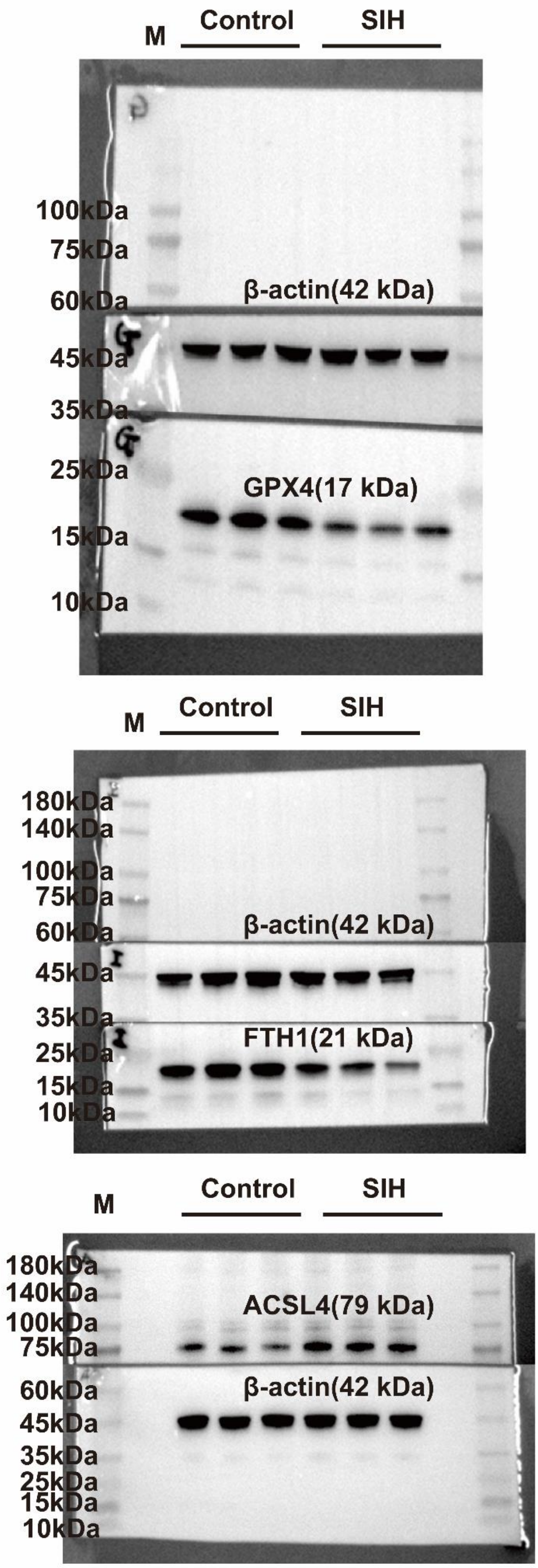

Full unedited blot for Figure 1I. M, marker; SIH, stress-induced hypertension.

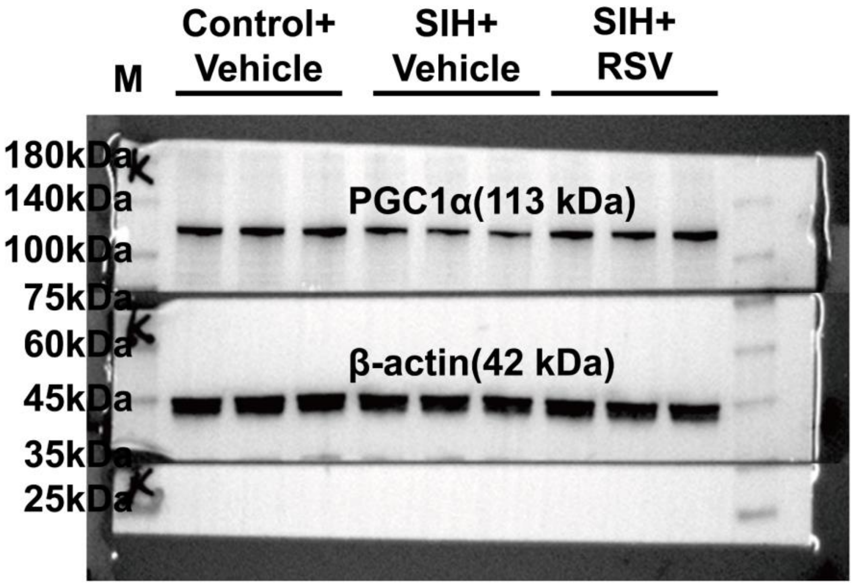
Control+ Vehicle
SIH+ Vehicle
SIH+ RSV
M
180kDa
140kDa
100kDa
75kDa
60kDa
45kDa
35kDa
25kDa
PGC1α(113 kDa)
β-actin(42 kDa)

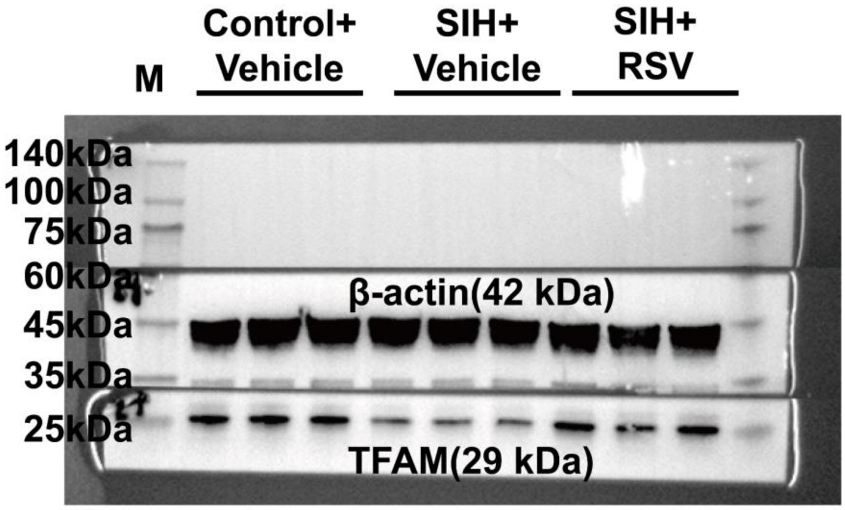
Control+ Vehicle
SIH+ Vehicle
SIH+ RSV
M
140kDa
100kDa
75kDa
60kDa
45kDa
35kDa
25kDa
β-actin(42 kDa)
TFAM(29 kDa)

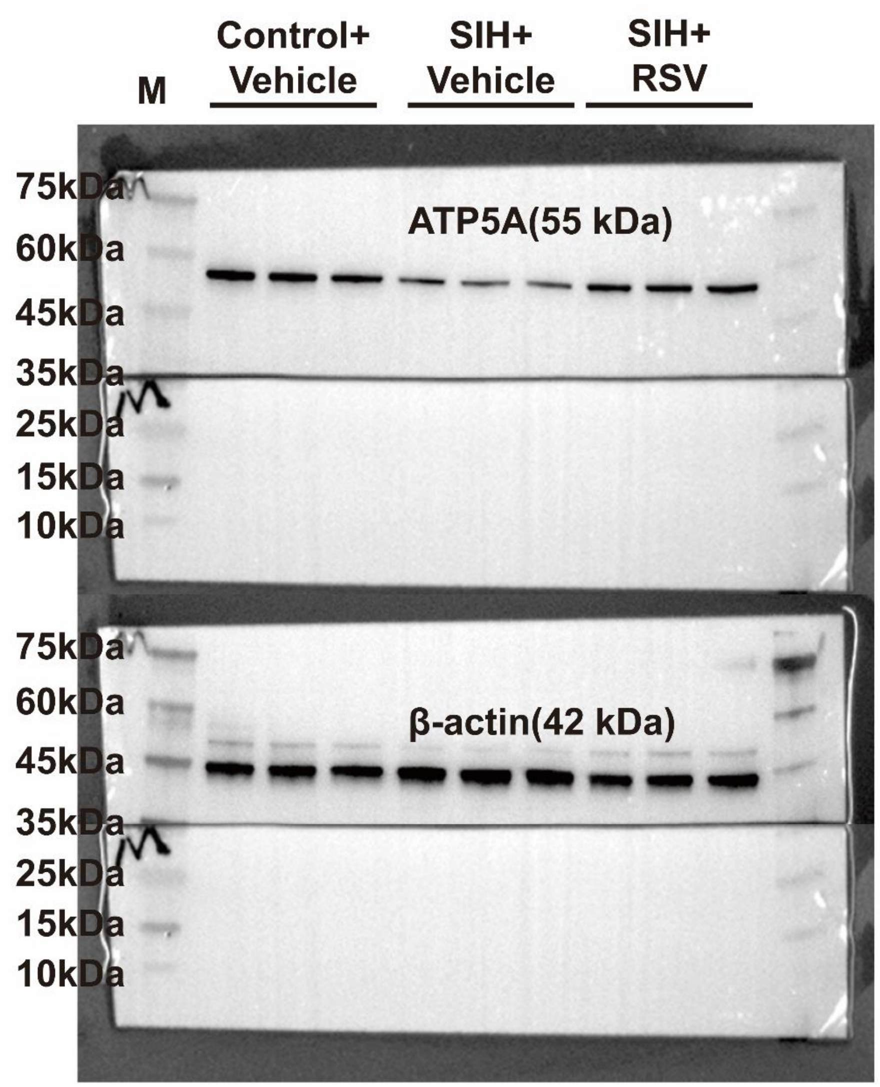
M
Control+ Vehicle
SIH+ Vehicle
SIH+ RSV
ATP5A(55 kDa)
β-actin(42 kDa)
75kDa
60kDa
45kDa
35kDa
25kDa
15kDa
10kDa
75kDa
60kDa
45kDa
35kDa
25kDa
15kDa
10kDa

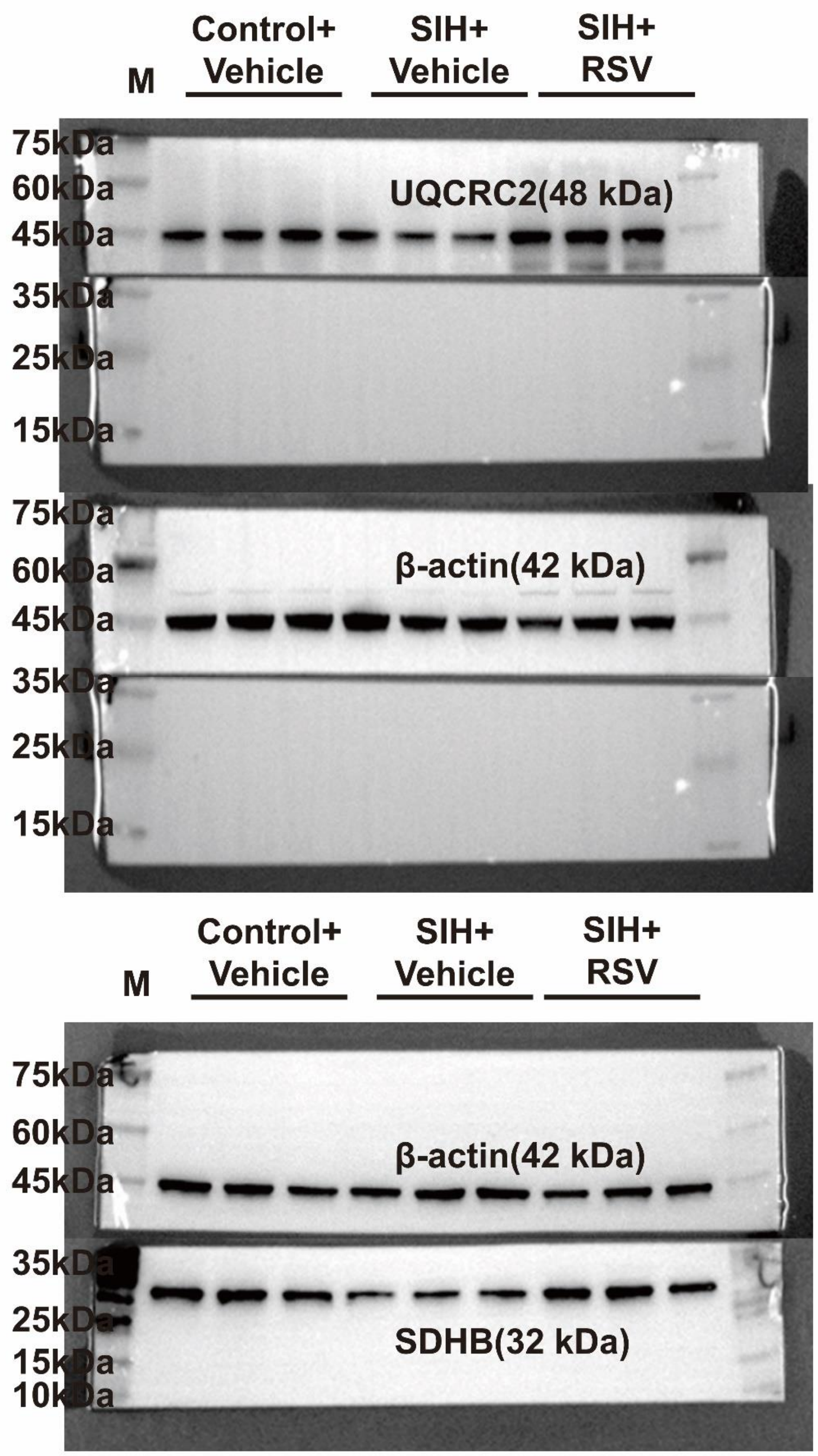

Full unedited blot for Figure 3A. M, marker; SIH, stress-induced hypertension; RSV, resveratrol.

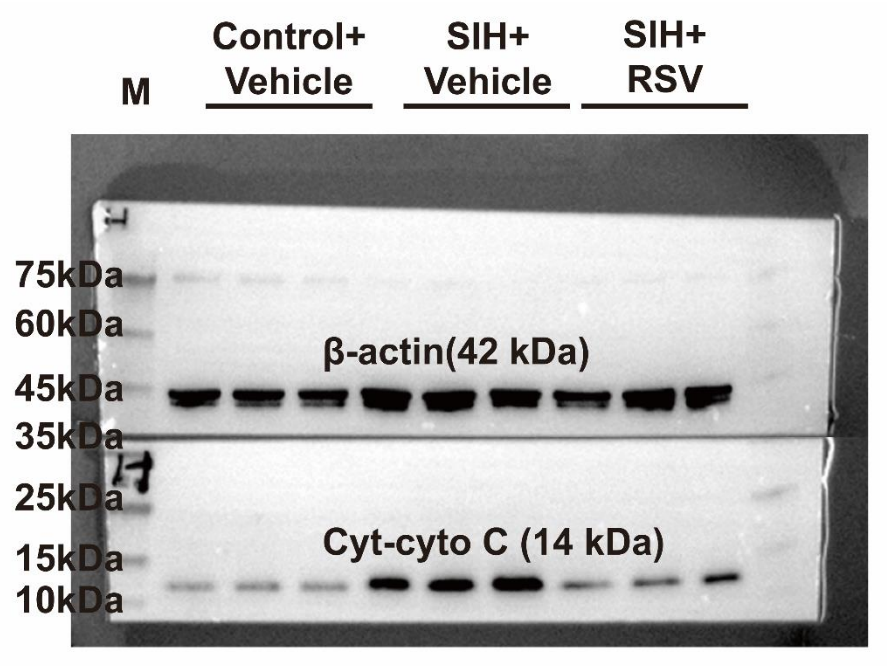

Full unedited blot for Figure 3E. M, marker; SIH, stress-induced hypertension; RSV, resveratrol.

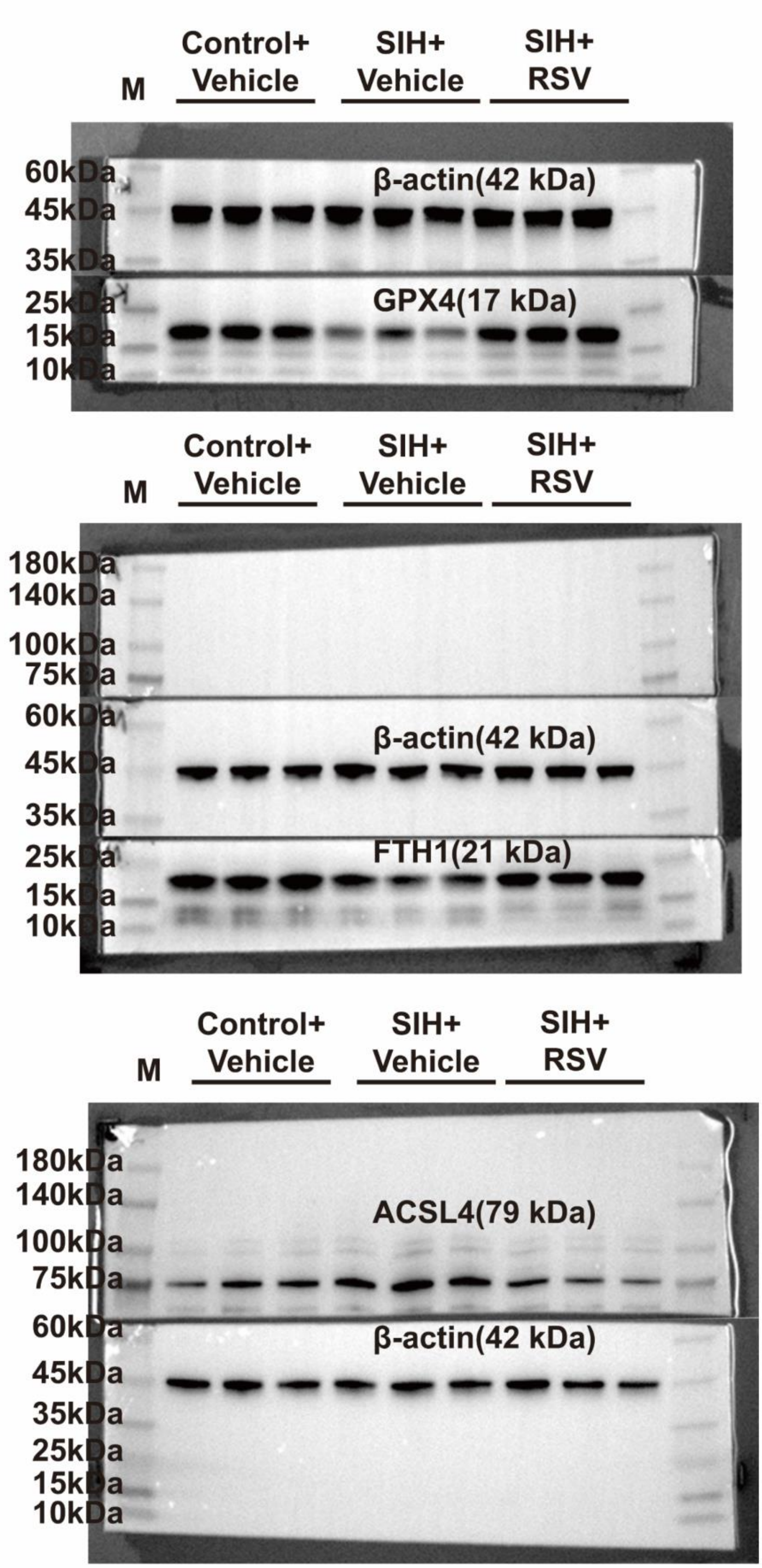

Full unedited blot for Figure 3H. M, marker; SIH, stress-induced hypertension; RSV, resveratrol.

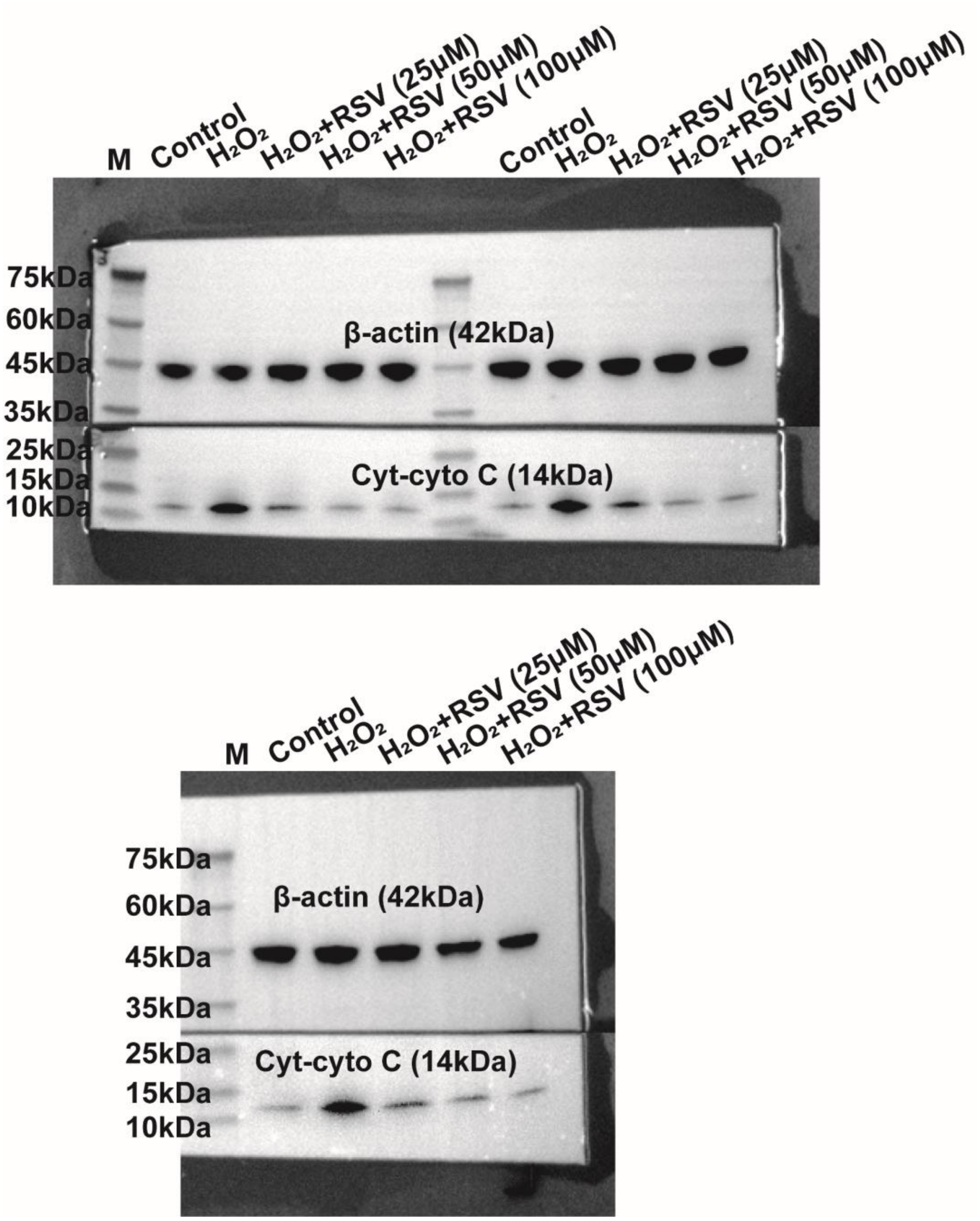

Full unedited blot for Figure 4E. M, marker; RSV, resveratrol.

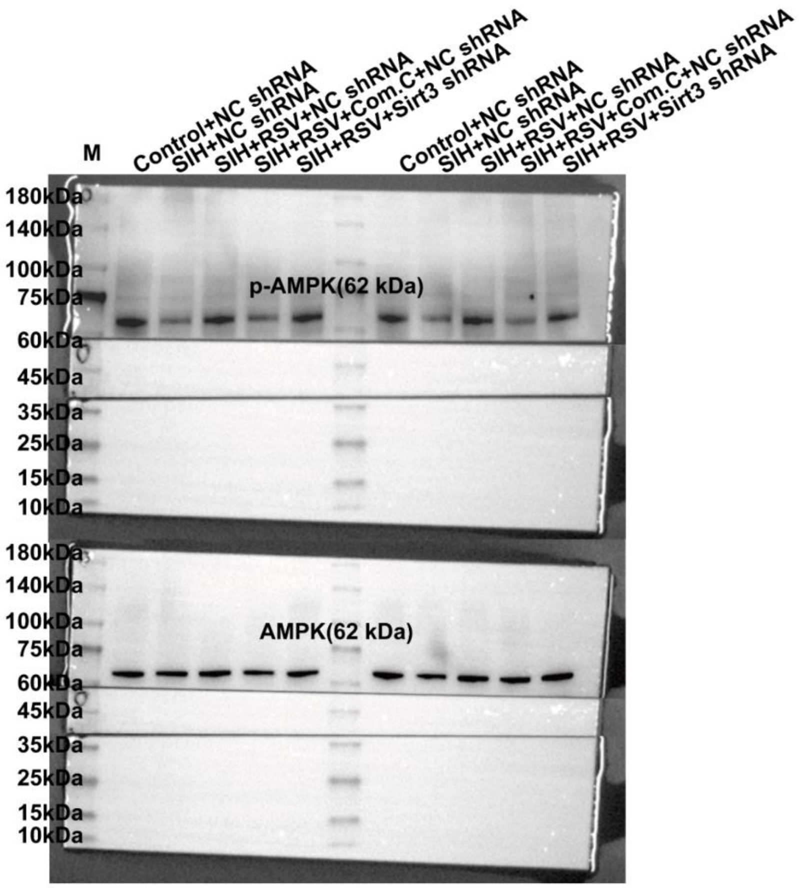
M
Control+NC shRNA
SIH+NC shRNA
SIH+RSV+NC shRNA
SIH+RSV+Com.C+NC shRNA
SIH+RSV+Sirt3 shRNA
Control+NC shRNA
SIH+NC shRNA
SIH+RSV+NC shRNA
SIH+RSV+Com.C+NC shRNA
SIH+RSV+Sirt3 shRNA
180kDa
140kDa
100kDa
75kDa
60kDa
45kDa
35kDa
25kDa
15kDa
10kDa
p-AMPK(62 kDa)
180kDa
140kDa
100kDa
75kDa
60kDa
45kDa
35kDa
25kDa
15kDa
10kDa
AMPK(62 kDa)

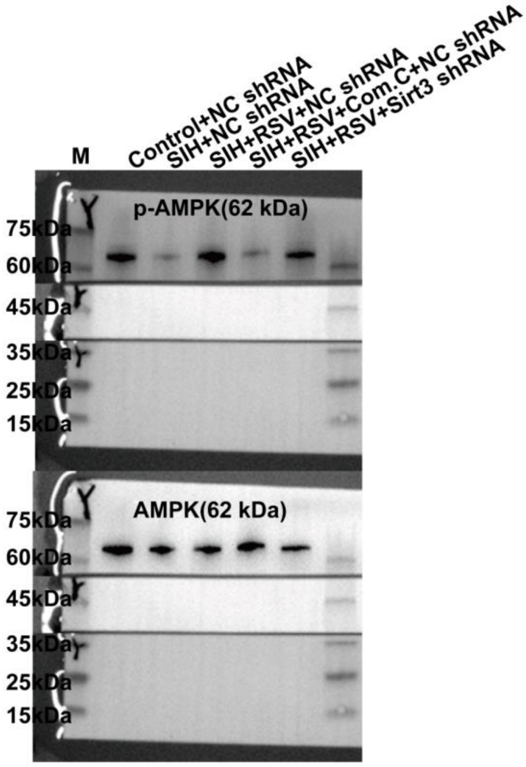
M
Control+NC shRNA
SIH+NC shRNA
SIH+RSV+NC shRNA
SIH+RSV+Com.C+NC shRNA
SIH+RSV+Sirt3 shRNA
p-AMPK(62 kDa)
75kDa
60kDa
45kDa
35kDa
25kDa
15kDa
AMPK(62 kDa)
75kDa
60kDa
45kDa
35kDa
25kDa
15kDa

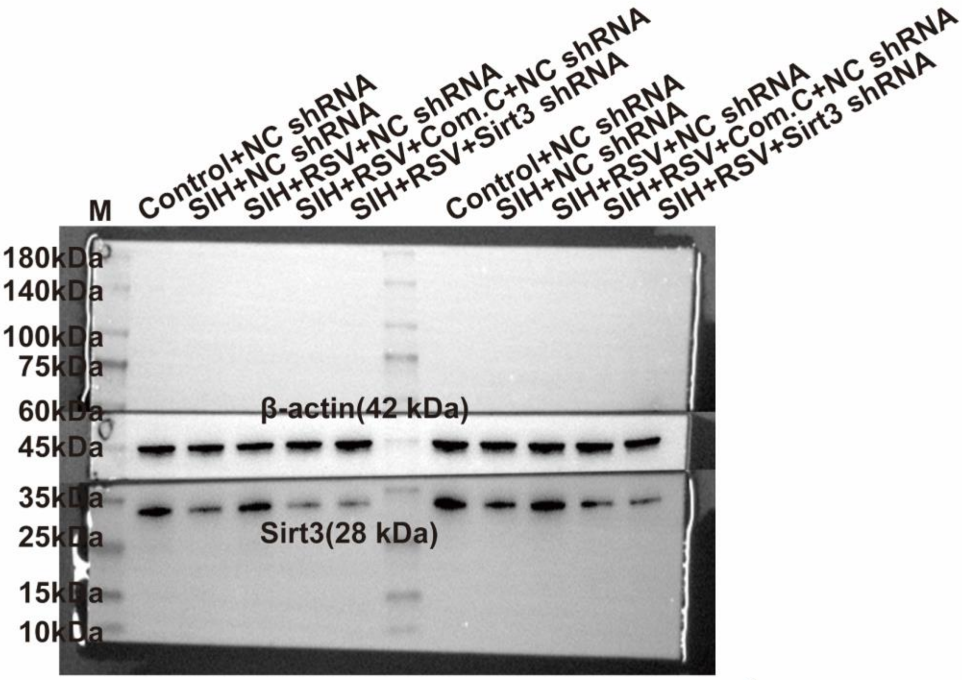
M
Control+NC shRNA
SIH+NC shRNA
SIH+RSV+NC shRNA
SIH+RSV+Com.C+NC shRNA
SIH+RSV+Sirt3 shRNA
Control+NC shRNA
SIH+NC shRNA
SIH+RSV+NC shRNA
SIH+RSV+Com.C+NC shRNA
SIH+RSV+Sirt3 shRNA
180kDa
140kDa
100kDa
75kDa
60kDa
45kDa
35kDa
25kDa
15kDa
10kDa
β-actin(42 kDa)
Sirt3(28 kDa)

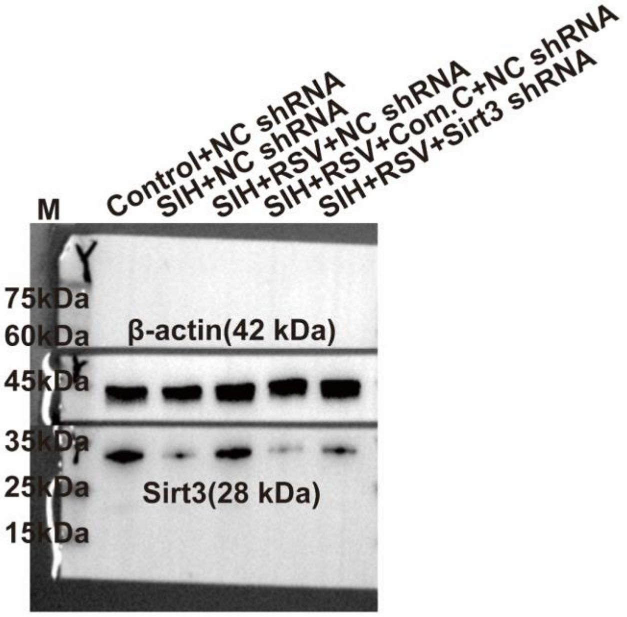
M
Control+NC shRNA
SIH+NC shRNA
SIH+RSV+NC shRNA
SIH+RSV+Com.C+NC shRNA
SIH+RSV+Sirt3 shRNA
75kDa
60kDa
45kDa
35kDa
25kDa
15kDa
β-actin(42 kDa)
Sirt3(28 kDa)

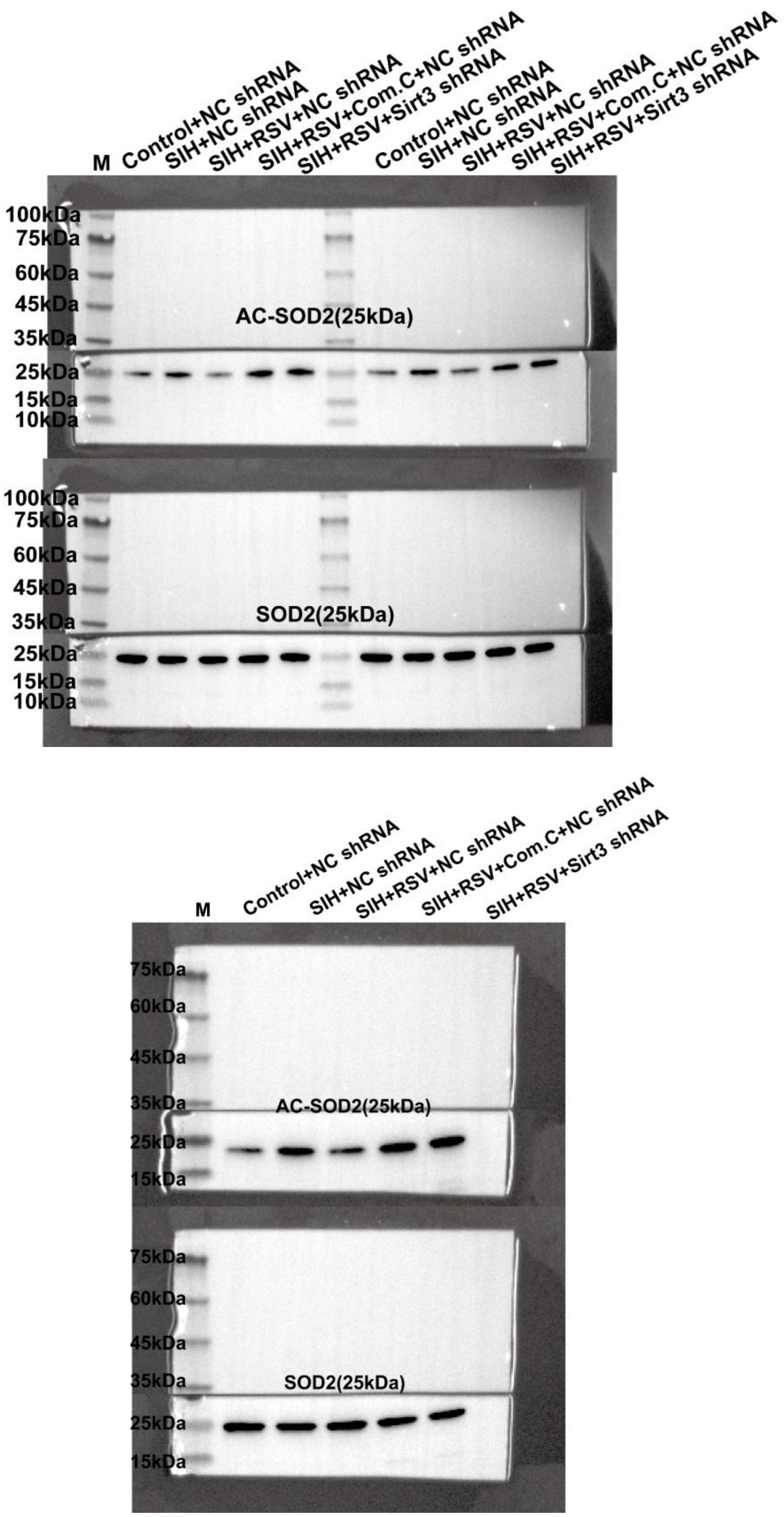

Full unedited blot for Figure 5B. M, marker; SIH, stress-induced hypertension; RSV, resveratrol; Com.C, compound C; NC, negative control.

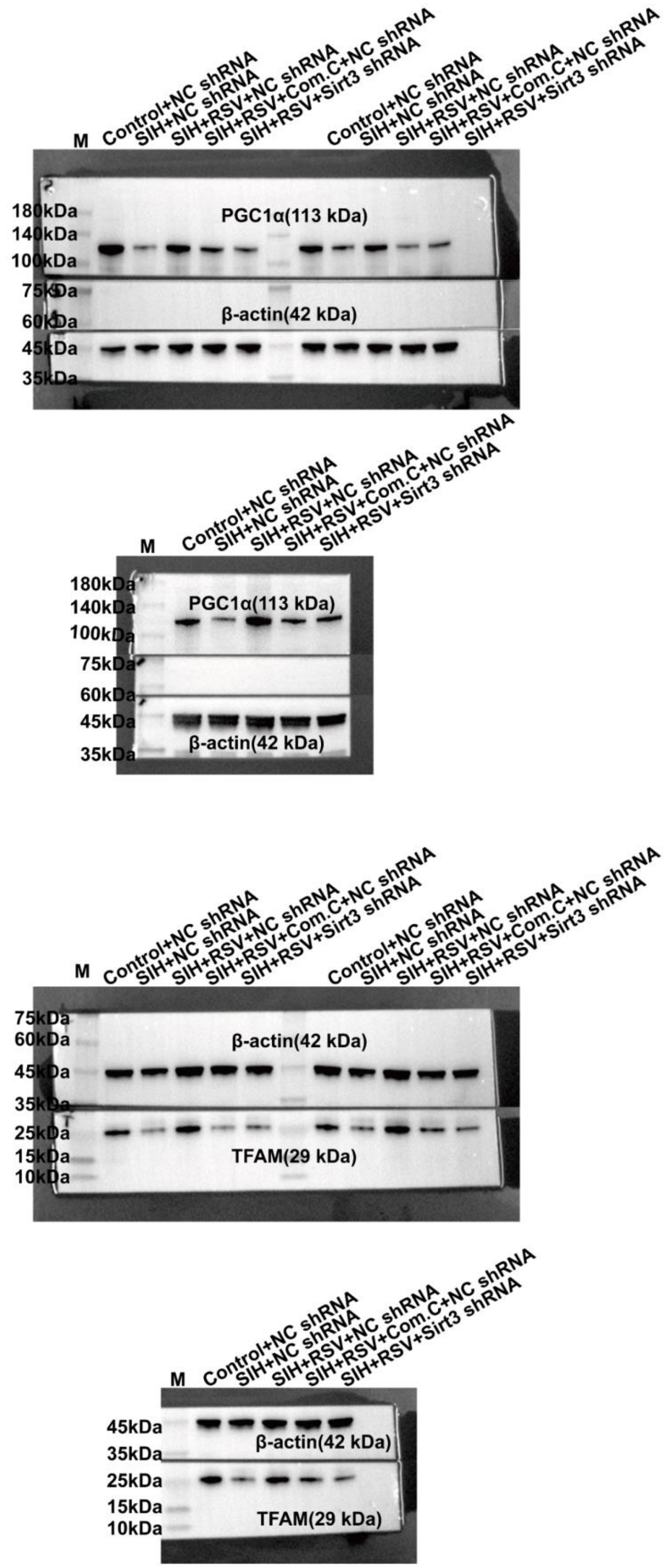

Full unedited blot for Figure 5C. M, marker; SIH, stress-induced hypertension; RSV, resveratrol; Com.C, compound C; NC, negative control.

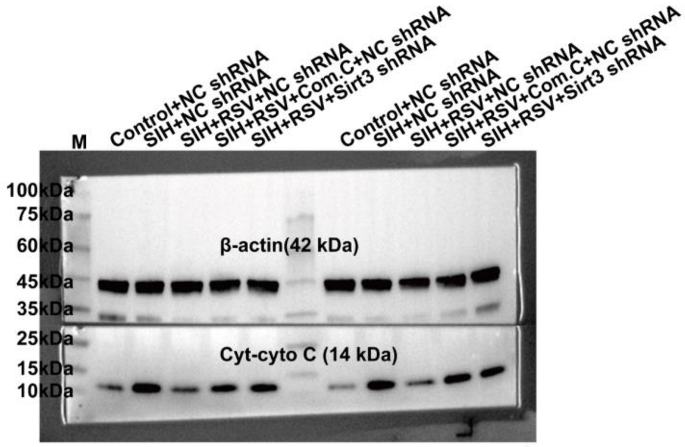

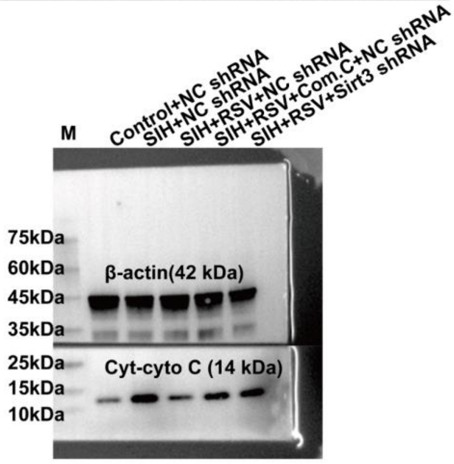

Full unedited blot for Figure 5E. M, marker; SIH, stress-induced hypertension; RSV, resveratrol; Com.C, compound C; NC, negative control.

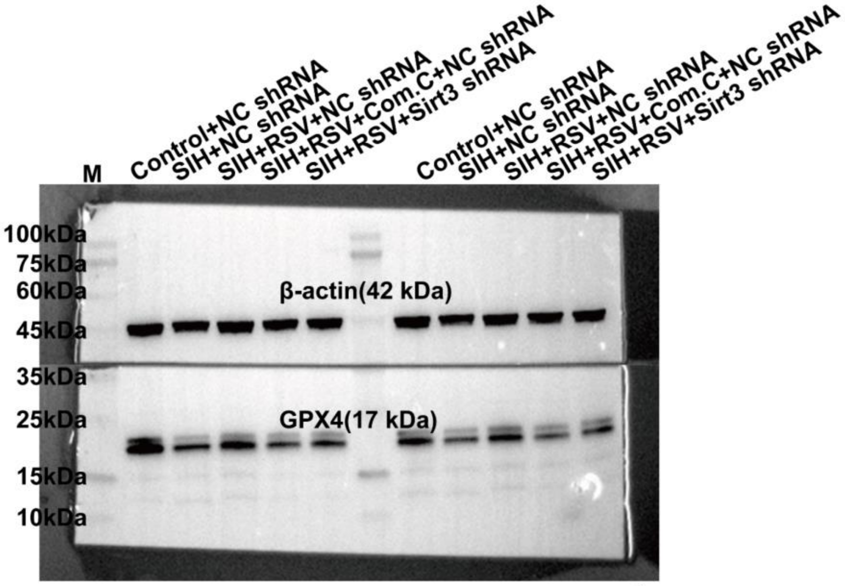
M
Control+NC shRNA
SIH+NC shRNA
SIH+RSV+NC shRNA
SIH+RSV+Com.C+NC shRNA
SIH+RSV+Sirt3 shRNA
Control+NC shRNA
SIH+NC shRNA
SIH+RSV+NC shRNA
SIH+RSV+Com.C+NC shRNA
SIH+RSV+Sirt3 shRNA
100kDa
75kDa
60kDa
45kDa
35kDa
25kDa
15kDa
10kDa
β-actin(42 kDa)
GPX4(17 kDa)

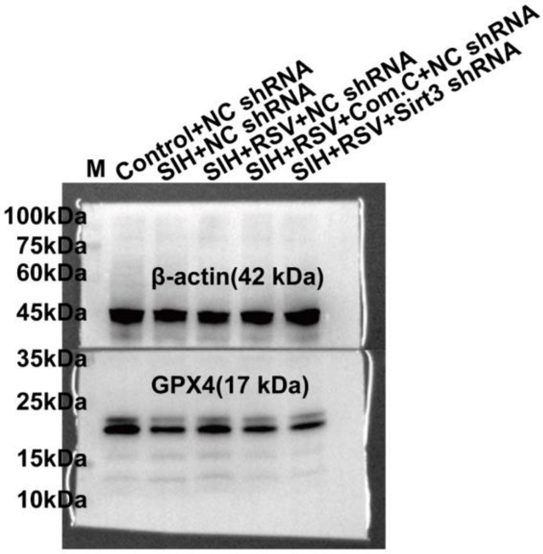
M
Control+NC shRNA
SIH+NC shRNA
SIH+RSV+NC shRNA
SIH+RSV+Com.C+NC shRNA
SIH+RSV+Sirt3 shRNA
100kDa
75kDa
60kDa
45kDa
35kDa
25kDa
15kDa
10kDa
β-actin(42 kDa)
GPX4(17 kDa)

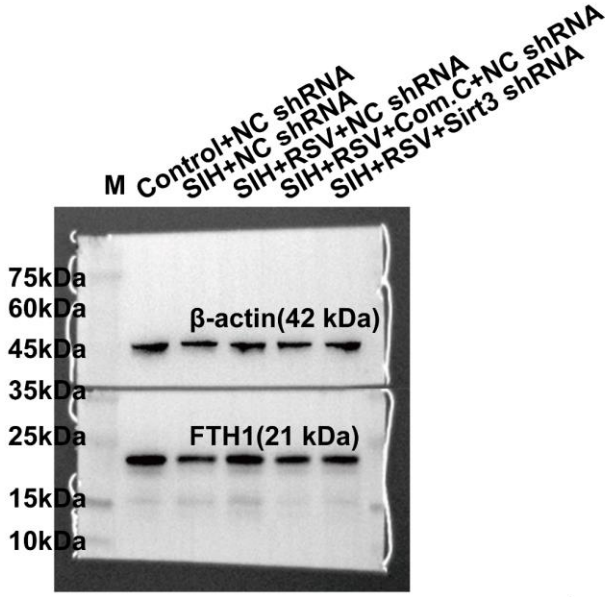
M
Control+NC shRNA
SIH+NC shRNA
SIH+RSV+NC shRNA
SIH+RSV+Com.C+NC shRNA
SIH+RSV+Sirt3 shRNA
75kDa
60kDa
β-actin(42 kDa)
45kDa
35kDa
25kDa
FTH1(21 kDa)
15kDa
10kDa

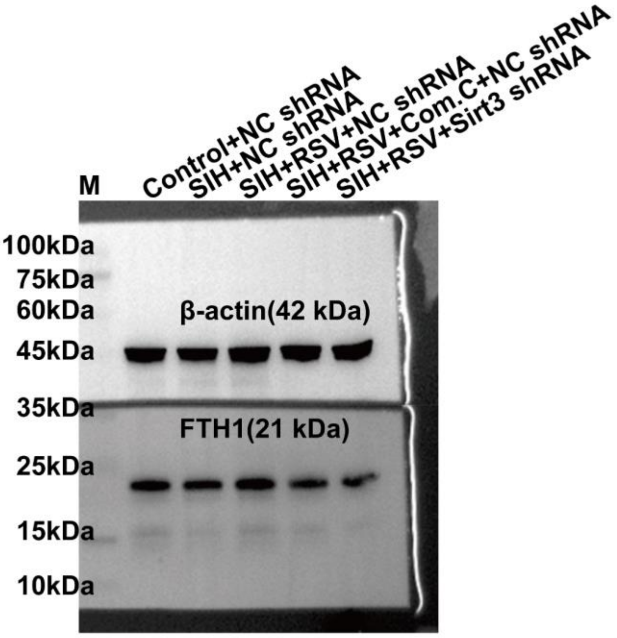
M
Control+NC shRNA
SIH+NC shRNA
SIH+RSV+NC shRNA
SIH+RSV+Com.C+NC shRNA
SIH+RSV+Sirt3 shRNA
100kDa
75kDa
60kDa
β-actin(42 kDa)
45kDa
35kDa
FTH1(21 kDa)
25kDa
15kDa
10kDa

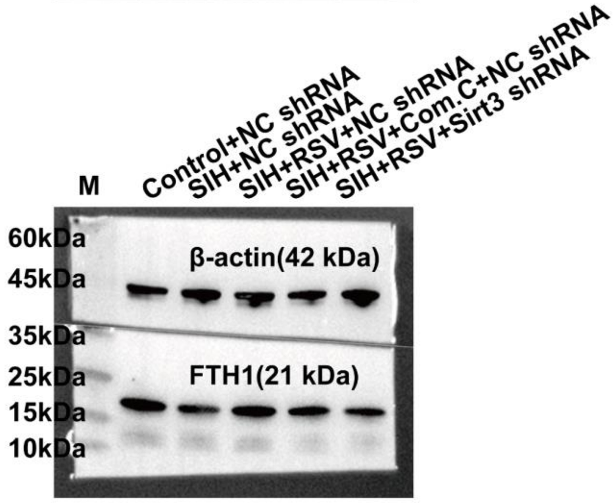
M
Control+NC shRNA
SIH+NC shRNA
SIH+RSV+NC shRNA
SIH+RSV+Com.C+NC shRNA
SIH+RSV+Sirt3 shRNA
60kDa
β-actin(42 kDa)
45kDa
35kDa
25kDa
FTH1(21 kDa)
15kDa
10kDa

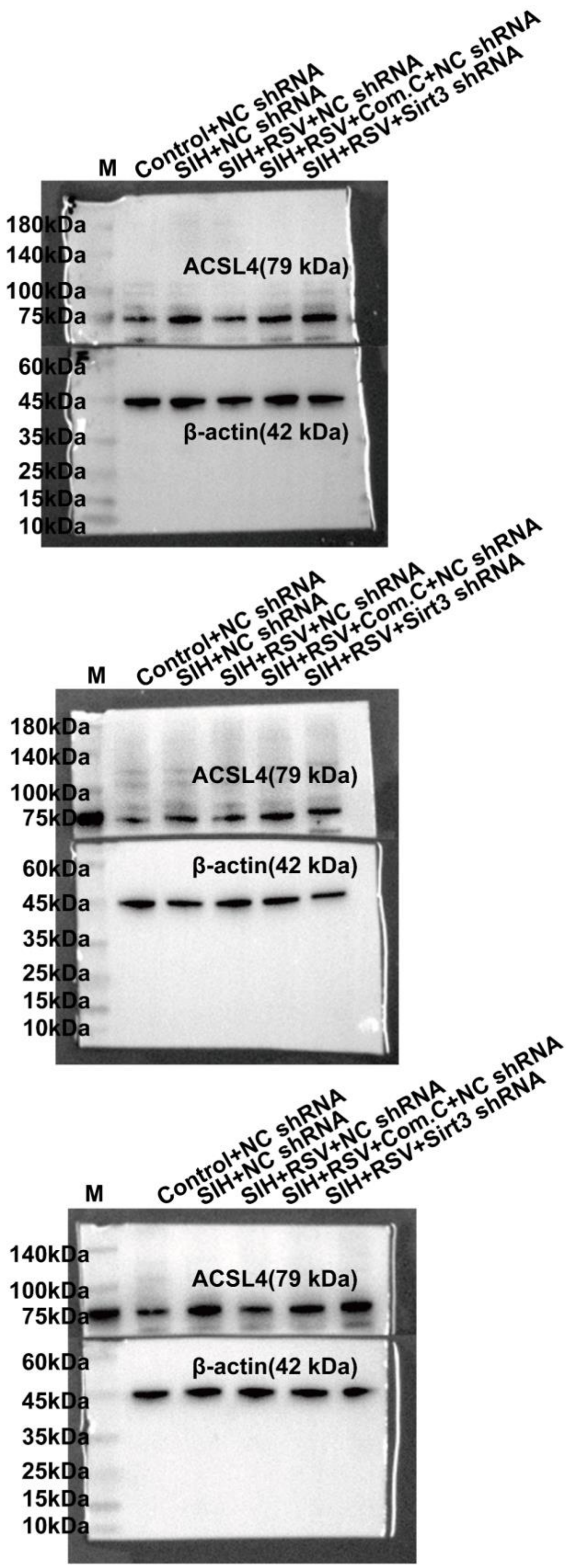

Full unedited blot for Figure 5G. M, marker; SIH, stress-induced hypertension; RSV, resveratrol; Com.C, compound C; NC, negative control.

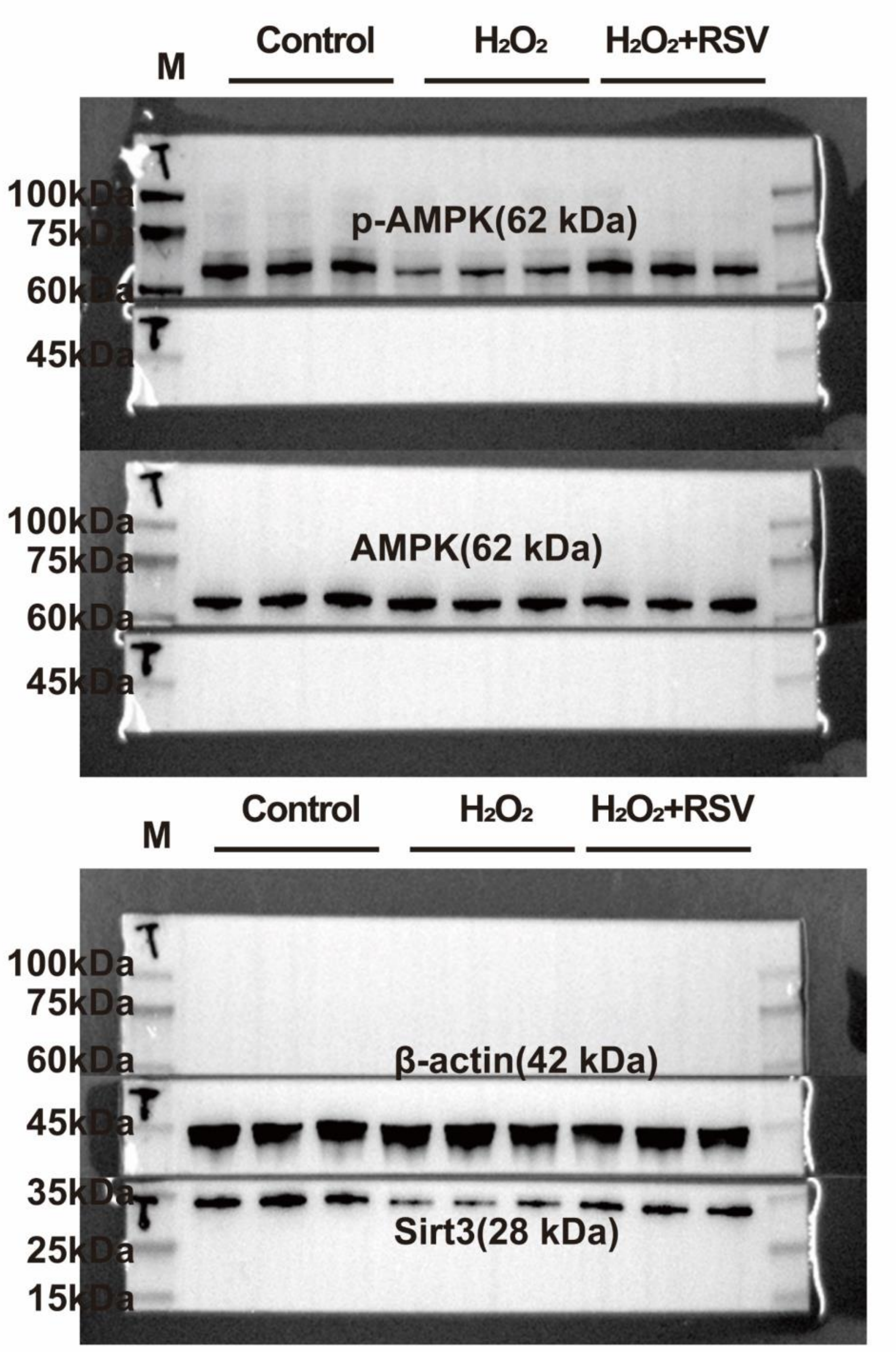

M
Control
H_2O_2
H_2O_2+RSV
100kDa
75kDa
60kDa
45kDa
p-AMPK(62 kDa)
AMPK(62 kDa)
35kDa
25kDa
15kDa
β-actin(42 kDa)
Sirt3(28 kDa)

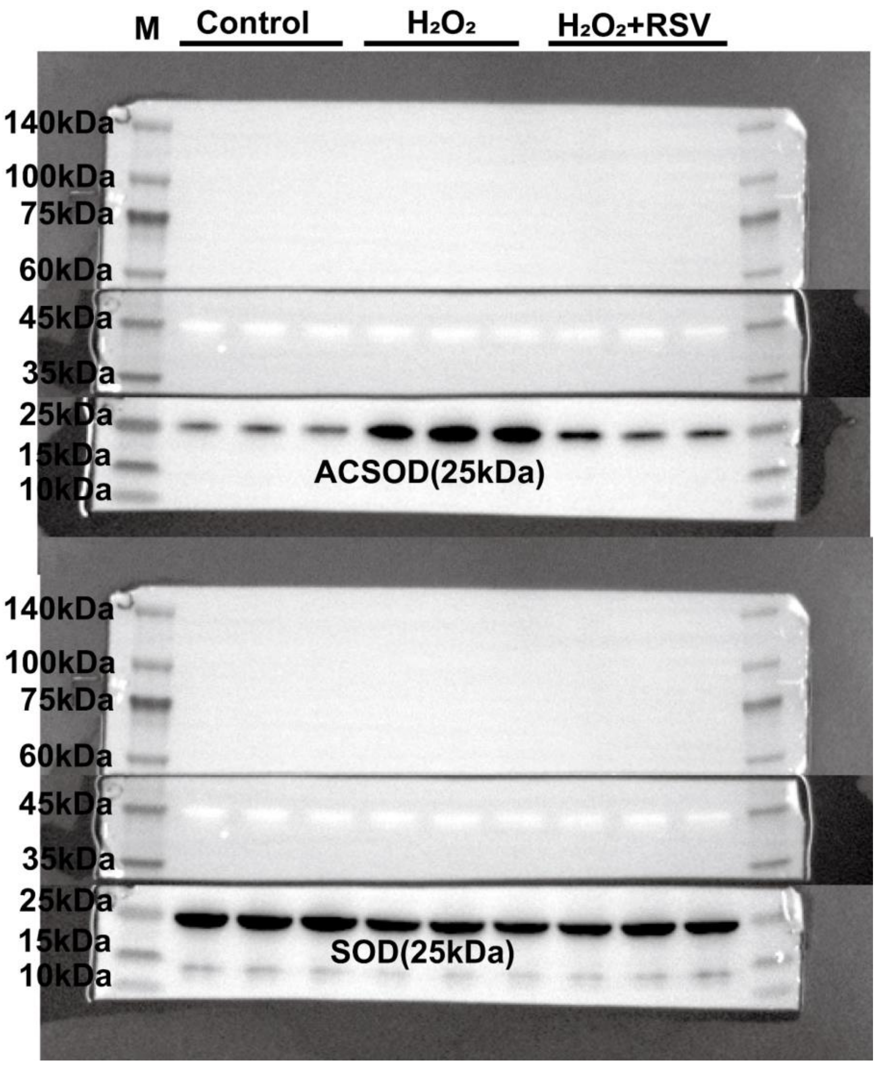

Full unedited blot for Figure 6A. M, marker; RSV, resveratrol.

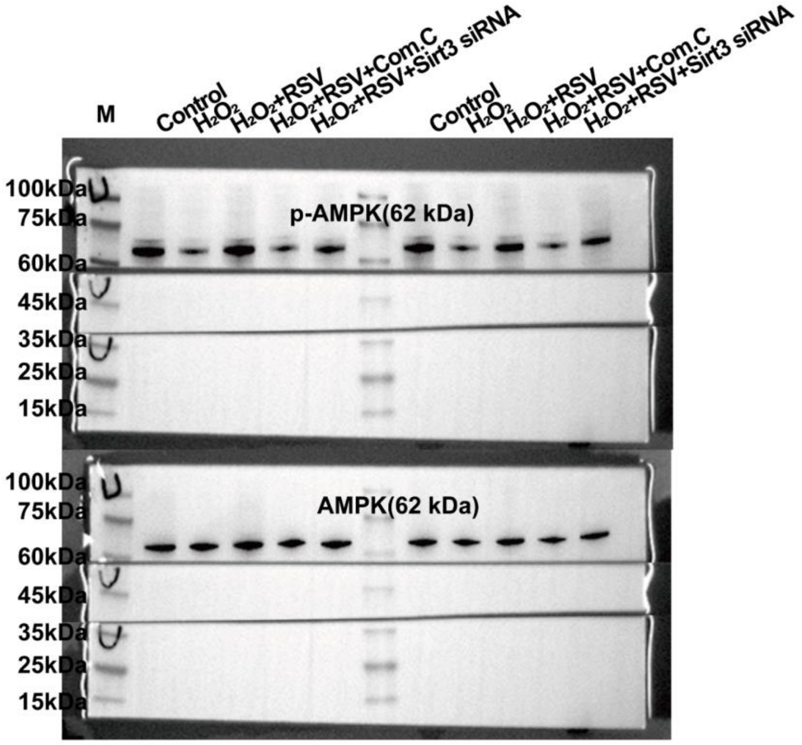
M
Control
H_2O_2
H_2O_2+RSV
H_2O_2+RSV+Com.C
H_2O_2+RSV+Sirt3 siRNA
Control
H_2O_2
H_2O_2+RSV
H_2O_2+RSV+Com.C
H_2O_2+RSV+Sirt3 siRNA
100kDa
75kDa
60kDa
45kDa
35kDa
25kDa
15kDa
p-AMPK(62 kDa)
100kDa
75kDa
60kDa
45kDa
35kDa
25kDa
15kDa
AMPK(62 kDa)

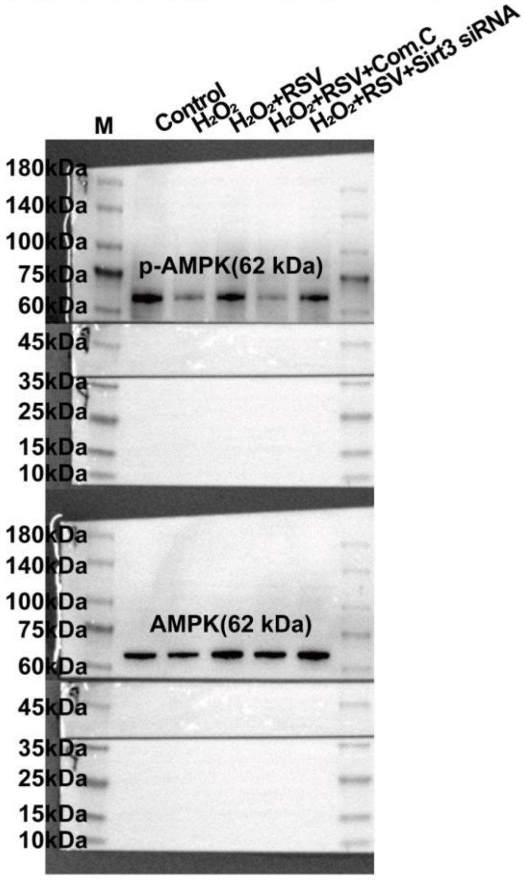
M
Control
H_2O_2
H_2O_2+RSV
H_2O_2+RSV+Com.C
H_2O_2+RSV+Sirt3 siRNA
180kDa
140kDa
100kDa
75kDa
60kDa
45kDa
35kDa
25kDa
15kDa
10kDa
p-AMPK(62 kDa)
180kDa
140kDa
100kDa
75kDa
60kDa
45kDa
35kDa
25kDa
15kDa
10kDa
AMPK(62 kDa)

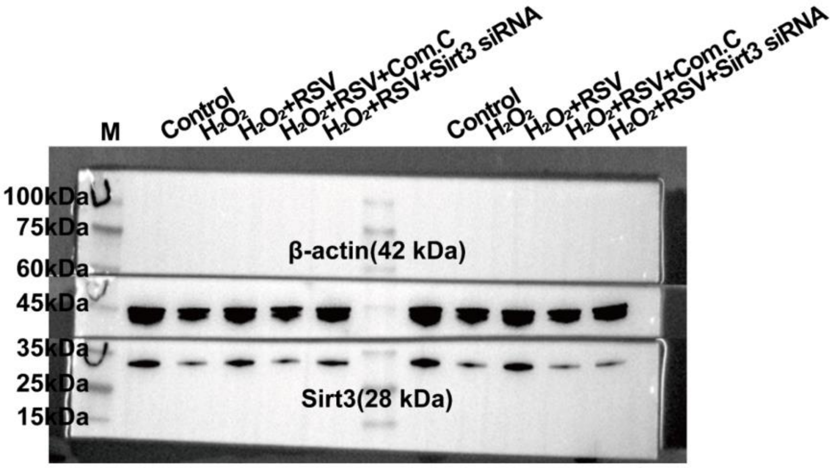
M
Control
H_2O_2
H_2O_2+RSV
H_2O_2+RSV+Com.C
H_2O_2+RSV+Sirt3 siRNA
Control
H_2O_2
H_2O_2+RSV
H_2O_2+RSV+Com.C
H_2O_2+RSV+Sirt3 siRNA
100kDa
75kDa
60kDa
45kDa
35kDa
25kDa
15kDa
β-actin(42 kDa)
Sirt3(28 kDa)

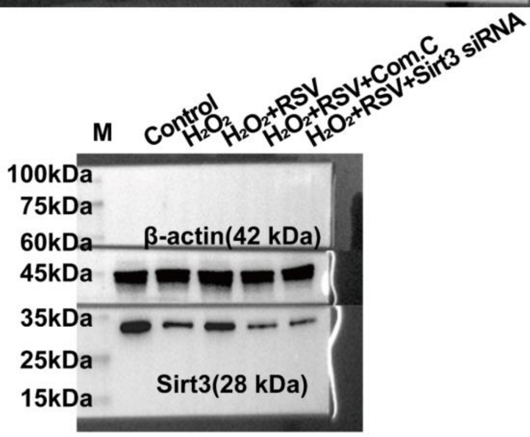
M
Control
H_2O_2
H_2O_2+RSV
H_2O_2+RSV+Com.C
H_2O_2+RSV+Sirt3 siRNA
100kDa
75kDa
60kDa
45kDa
35kDa
25kDa
15kDa
β-actin(42 kDa)
Sirt3(28 kDa)

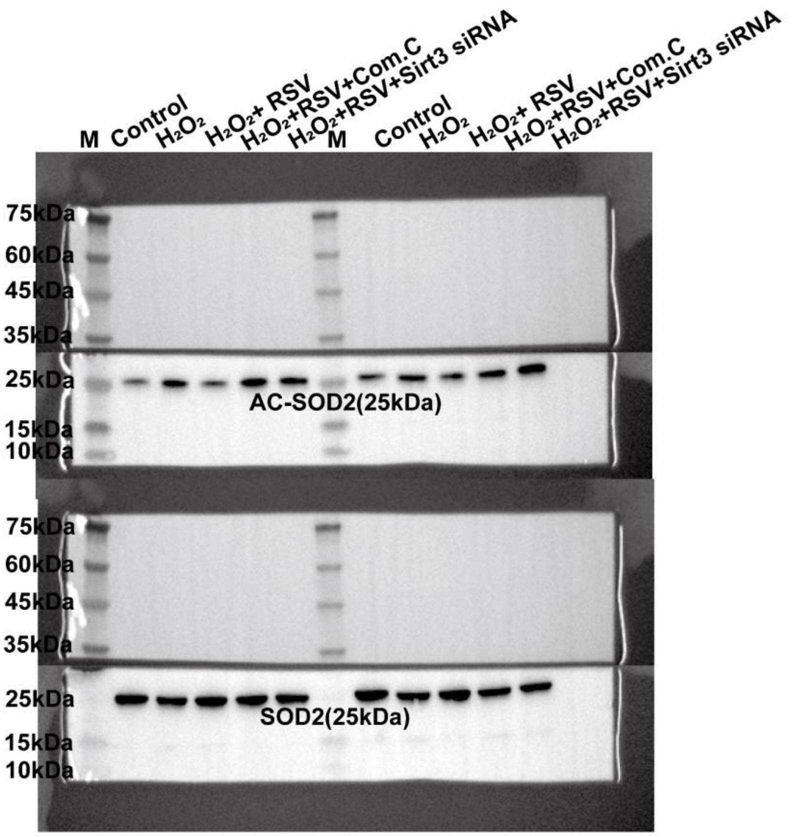

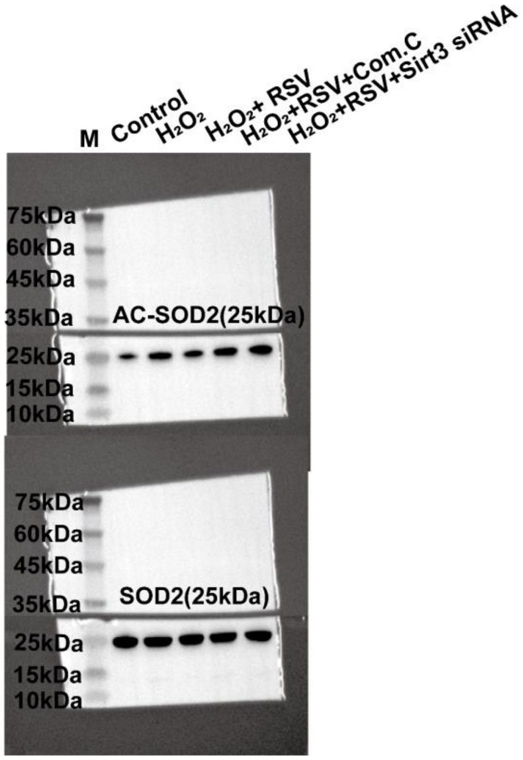

Full unedited blot for Figure 6B. M, marker; RSV, resveratrol; Com.C, compound C.

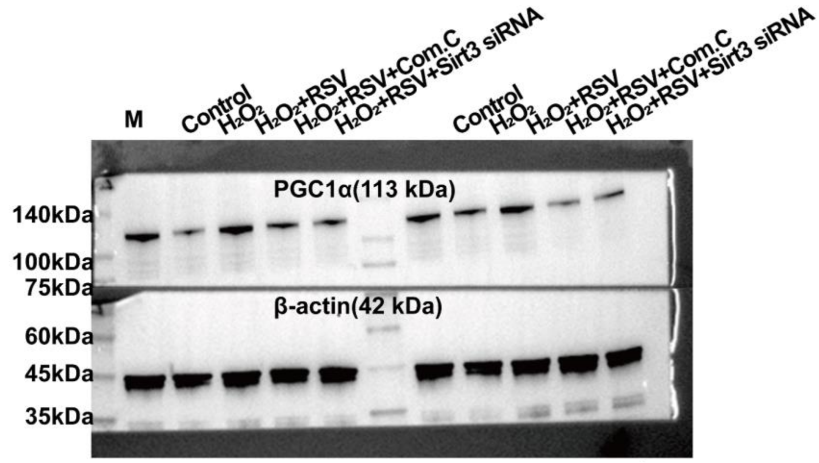
M
Control
H2O2
H2O2+RSV
H2O2+RSV+Com.C
H2O2+RSV+Sirt3 siRNA
Control
H2O2
H2O2+RSV
H2O2+RSV+Com.C
H2O2+RSV+Sirt3 siRNA
PGC1α(113 kDa)
140kDa
100kDa
75kDa
β-actin(42 kDa)
60kDa
45kDa
35kDa

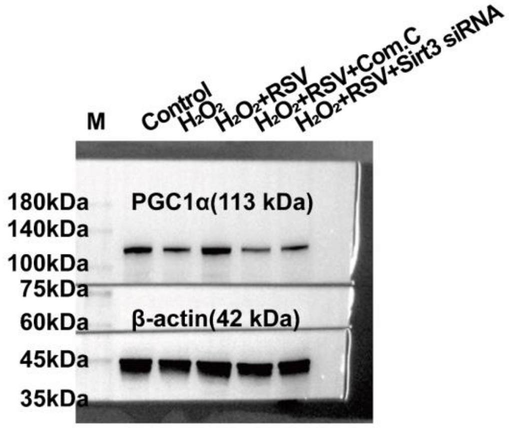
M
Control
H2O2
H2O2+RSV
H2O2+RSV+Com.C
H2O2+RSV+Sirt3 siRNA
180kDa
PGC1α(113 kDa)
140kDa
100kDa
75kDa
60kDa
β-actin(42 kDa)
45kDa
35kDa

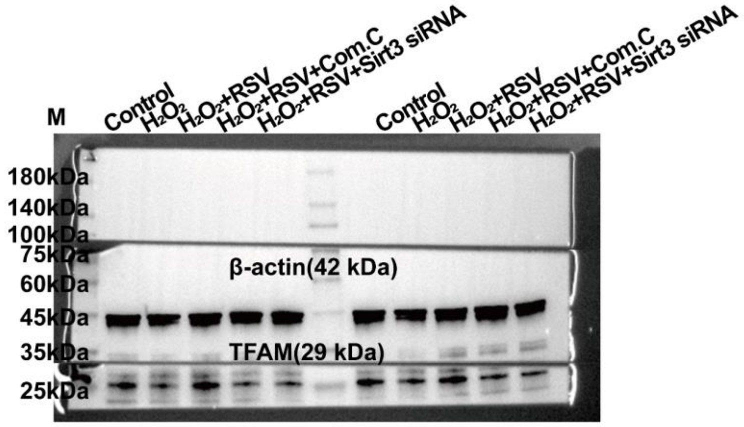
M
Control
H2O2
H2O2+RSV
H2O2+RSV+Com.C
H2O2+RSV+Sirt3 siRNA
Control
H2O2
H2O2+RSV
H2O2+RSV+Com.C
H2O2+RSV+Sirt3 siRNA
180kDa
140kDa
100kDa
75kDa
β-actin(42 kDa)
60kDa
45kDa
35kDa
TFAM(29 kDa)
25kDa

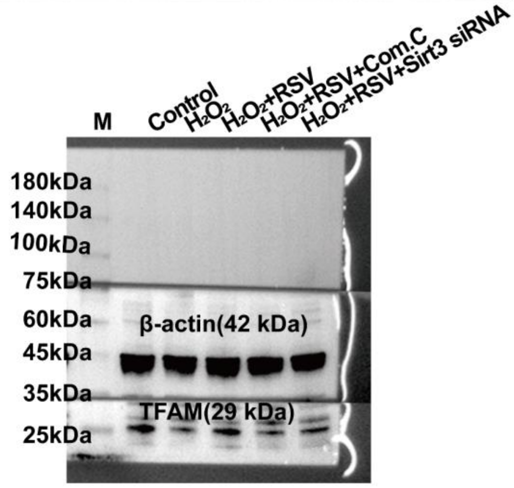
M
Control
H2O2
H2O2+RSV
H2O2+RSV+Com.C
H2O2+RSV+Sirt3 siRNA
180kDa
140kDa
100kDa
75kDa
60kDa
β-actin(42 kDa)
45kDa
35kDa
TFAM(29 kDa)
25kDa

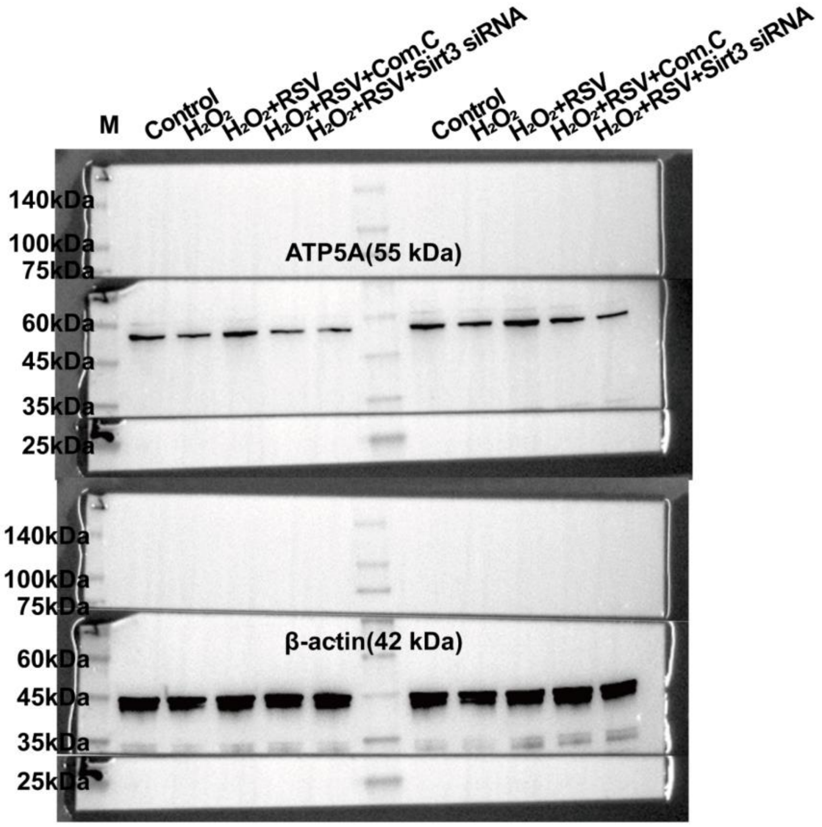
M
Control
H_2O_2
H_2O_2+RSV
H_2O_2+RSV+Com.C
H_2O_2+RSV+Sirt3 siRNA
Control
H_2O_2
H_2O_2+RSV
H_2O_2+RSV+Com.C
H_2O_2+RSV+Sirt3 siRNA
140kDa
100kDa
75kDa
60kDa
45kDa
35kDa
25kDa
ATP5A(55 kDa)
140kDa
100kDa
75kDa
60kDa
45kDa
35kDa
25kDa
β-actin(42 kDa)

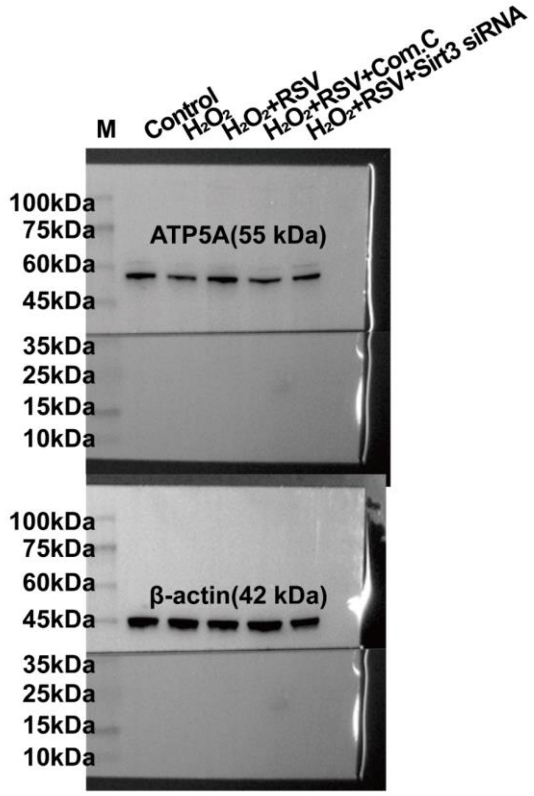
M
Control
H_2O_2
H_2O_2+RSV
H_2O_2+RSV+Com.C
H_2O_2+RSV+Sirt3 siRNA
100kDa
75kDa
60kDa
45kDa
35kDa
25kDa
15kDa
10kDa
ATP5A(55 kDa)
100kDa
75kDa
60kDa
45kDa
35kDa
25kDa
15kDa
10kDa
β-actin(42 kDa)

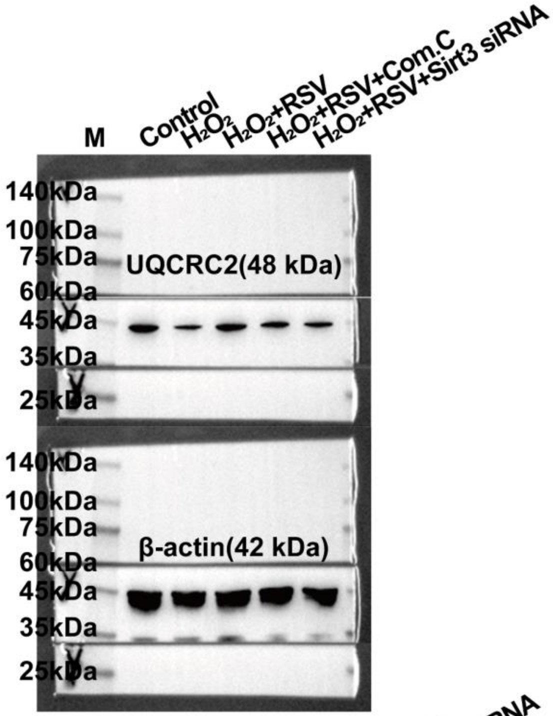
M
Control
H_2O_2
H_2O_2+RSV
H_2O_2+RSV+Com.C
H_2O_2+RSV+Sirt3 siRNA
140kDa
100kDa
75kDa
60kDa
45kDa
35kDa
25kDa
UQCRC2(48 kDa)
β-actin(42 kDa)

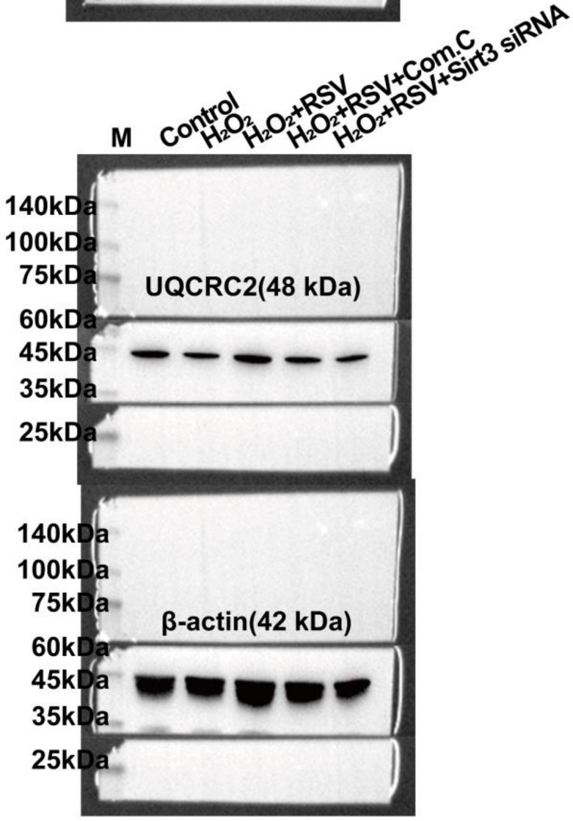
M
Control
H_2O_2
H_2O_2+RSV
H_2O_2+RSV+Com.C
H_2O_2+RSV+Sirt3 siRNA
140kDa
100kDa
75kDa
60kDa
45kDa
35kDa
25kDa
UQCRC2(48 kDa)
β-actin(42 kDa)

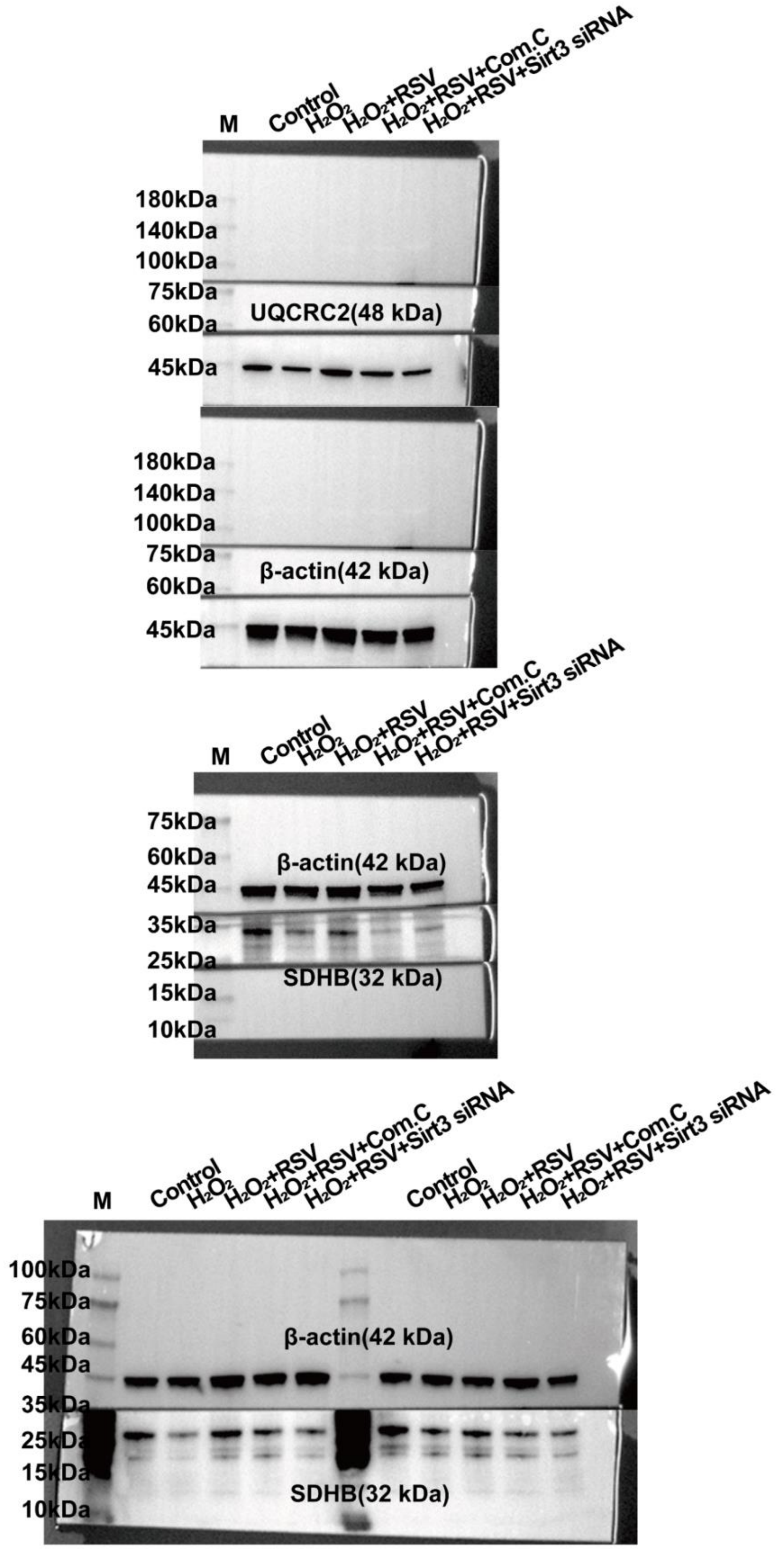

Full unedited blot for Figure 6C. M, marker; RSV, resveratrol; Com.C, compound C.

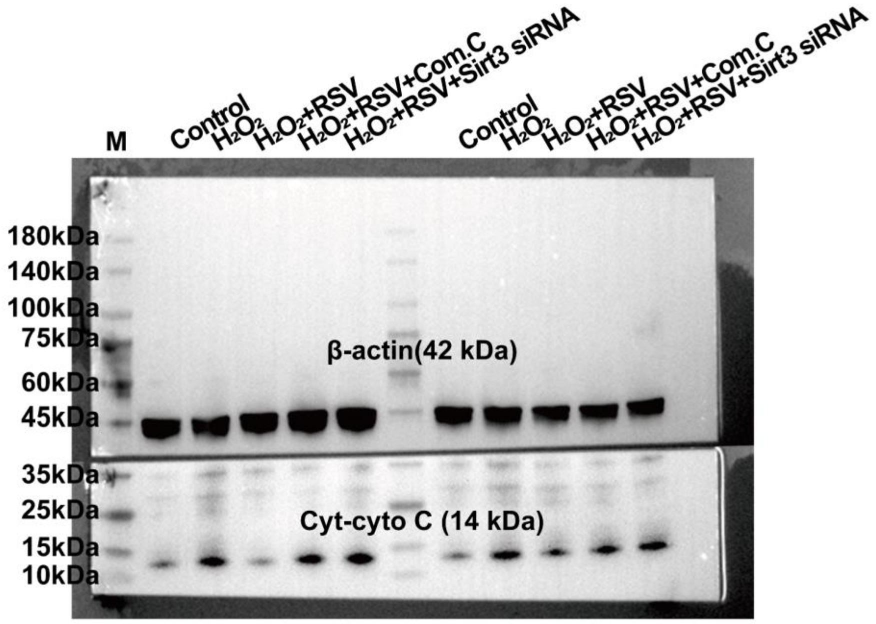

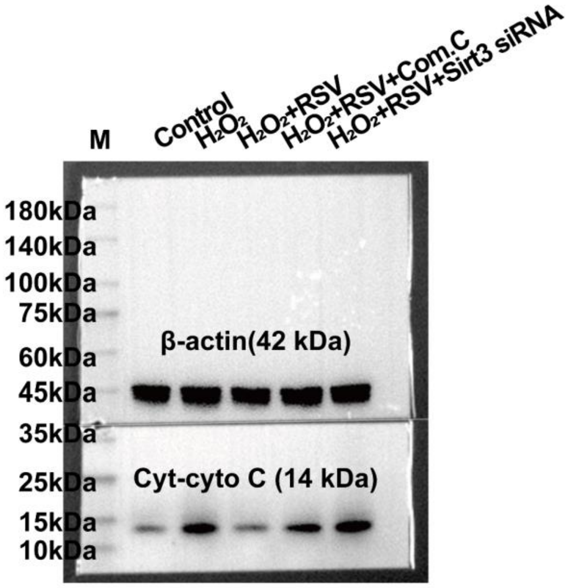

Full unedited blot for Figure 6F. M, marker; RSV, resveratrol; Com.C, compound C.

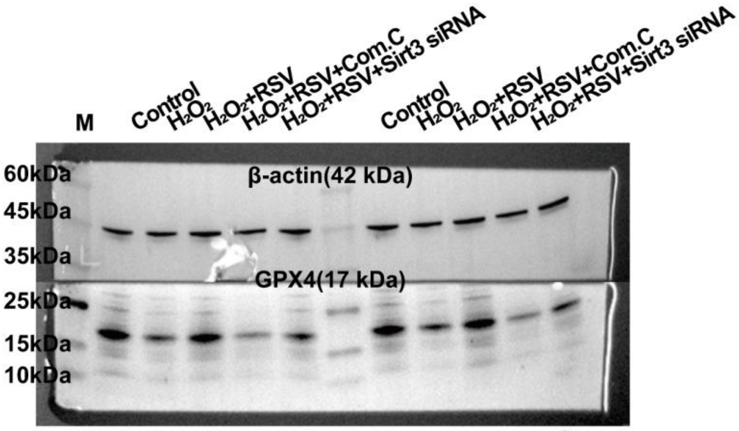
M
Control
H_2O_2
H_2O_2+RSV
H_2O_2+RSV+Com.C
H_2O_2+RSV+Sirt3 siRNA
Control
H_2O_2
H_2O_2+RSV
H_2O_2+RSV+Com.C
H_2O_2+RSV+Sirt3 siRNA
60kDa
45kDa
35kDa
25kDa
15kDa
10kDa
β-actin(42 kDa)
GPX4(17 kDa)

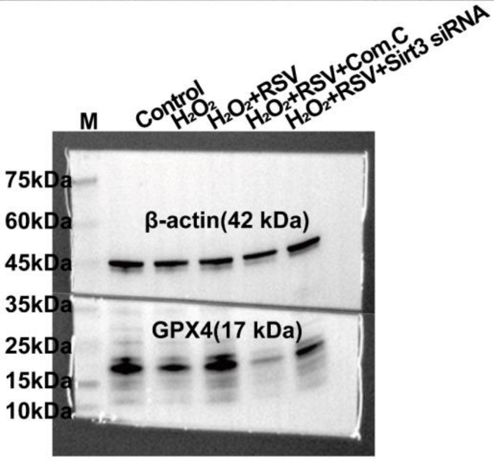
M
Control
H_2O_2
H_2O_2+RSV
H_2O_2+RSV+Com.C
H_2O_2+RSV+Sirt3 siRNA
75kDa
60kDa
45kDa
35kDa
25kDa
15kDa
10kDa
β-actin(42 kDa)
GPX4(17 kDa)

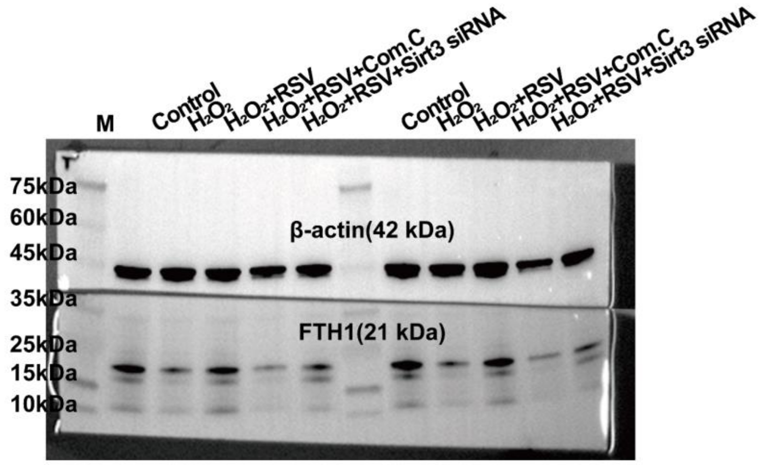
M
Control
H_2O_2
H_2O_2+RSV
H_2O_2+RSV+Com.C
H_2O_2+RSV+Sirt3 siRNA
Control
H_2O_2
H_2O_2+RSV
H_2O_2+RSV+Com.C
H_2O_2+RSV+Sirt3 siRNA
75kDa
60kDa
45kDa
35kDa
25kDa
15kDa
10kDa
β-actin(42 kDa)
FTH1(21 kDa)

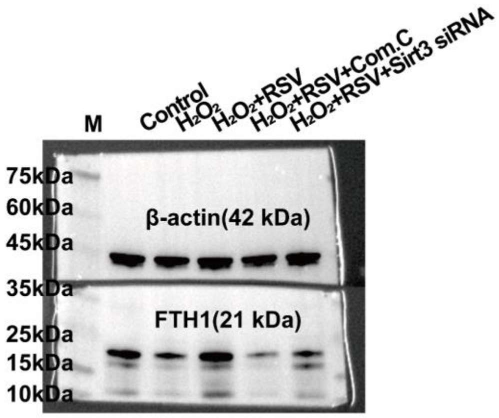
M
Control
H_2O_2
H_2O_2+RSV
H_2O_2+RSV+Com.C
H_2O_2+RSV+Sirt3 siRNA
75kDa
60kDa
45kDa
35kDa
25kDa
15kDa
10kDa
β-actin(42 kDa)
FTH1(21 kDa)

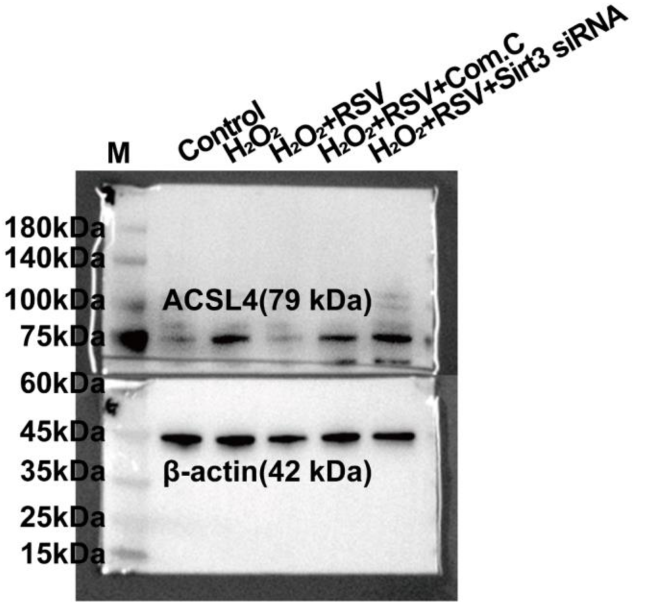

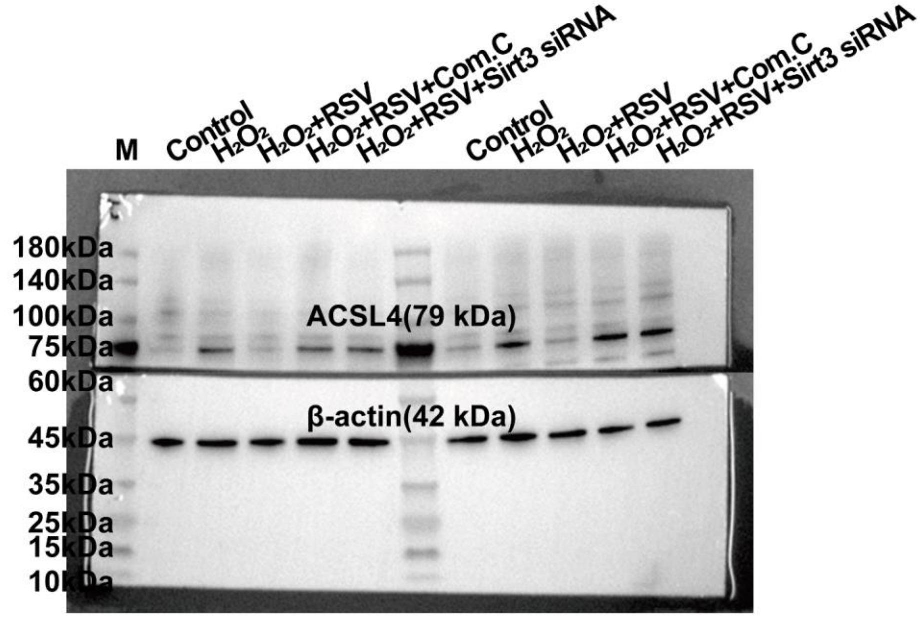

Full unedited blot for Figure 6H. M, marker; RSV, resveratrol; Com.C, compound C.

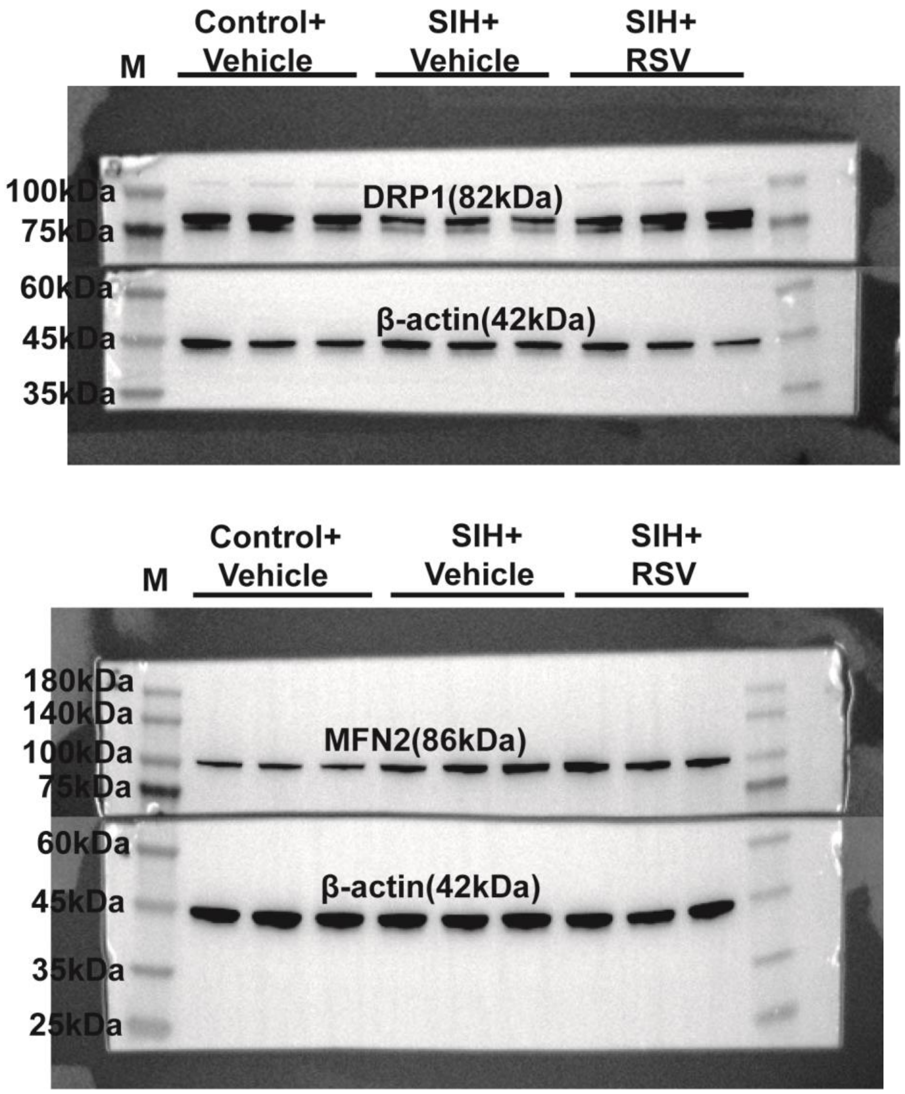

Full unedited blot for Figure S1A. M, marker; SIH, stress-induced hypertension; RSV, resveratrol.

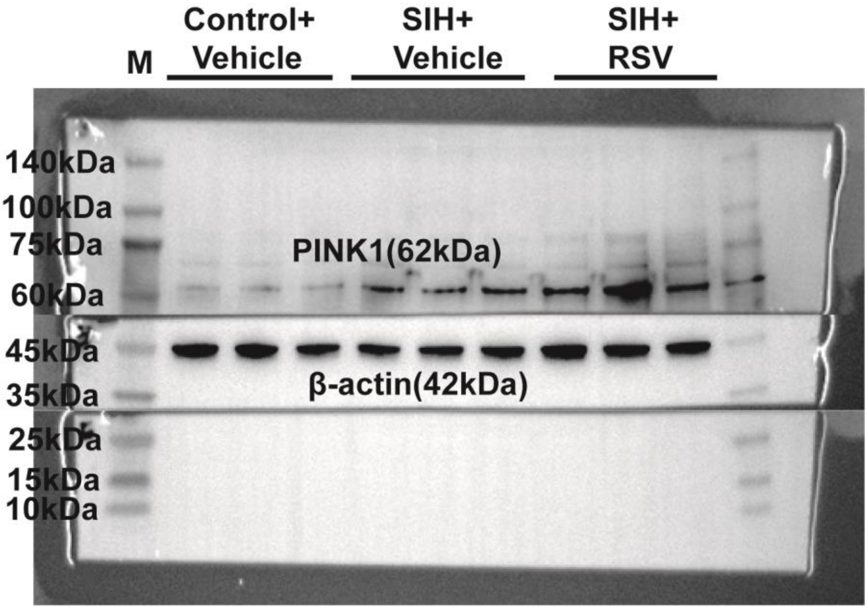

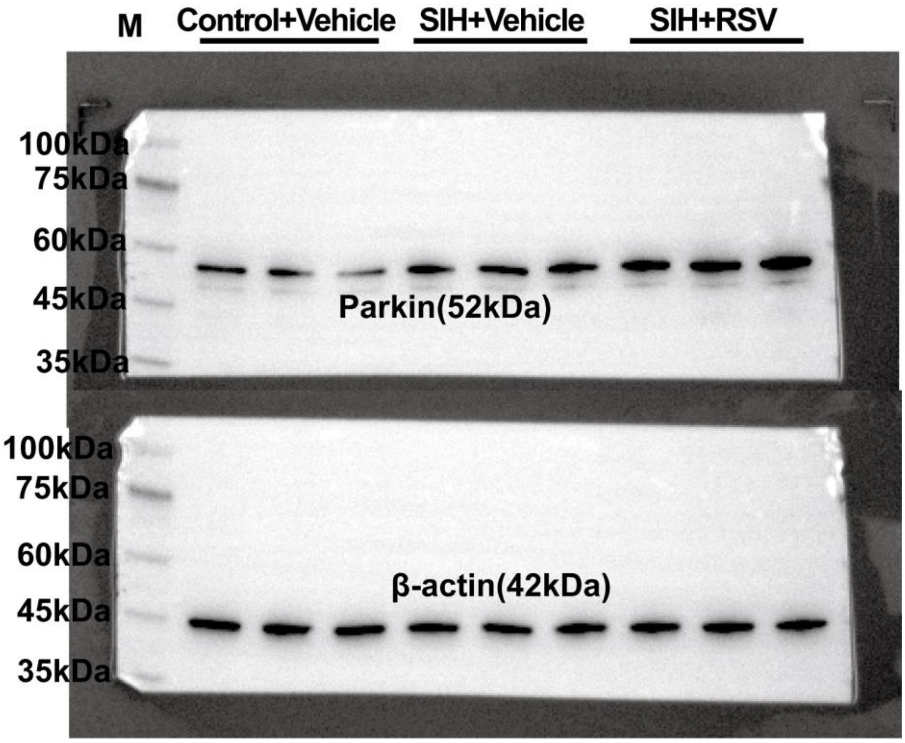

Full unedited blot for Figure S1B. M, marker; SIH, stress-induced hypertension; RSV, resveratrol.

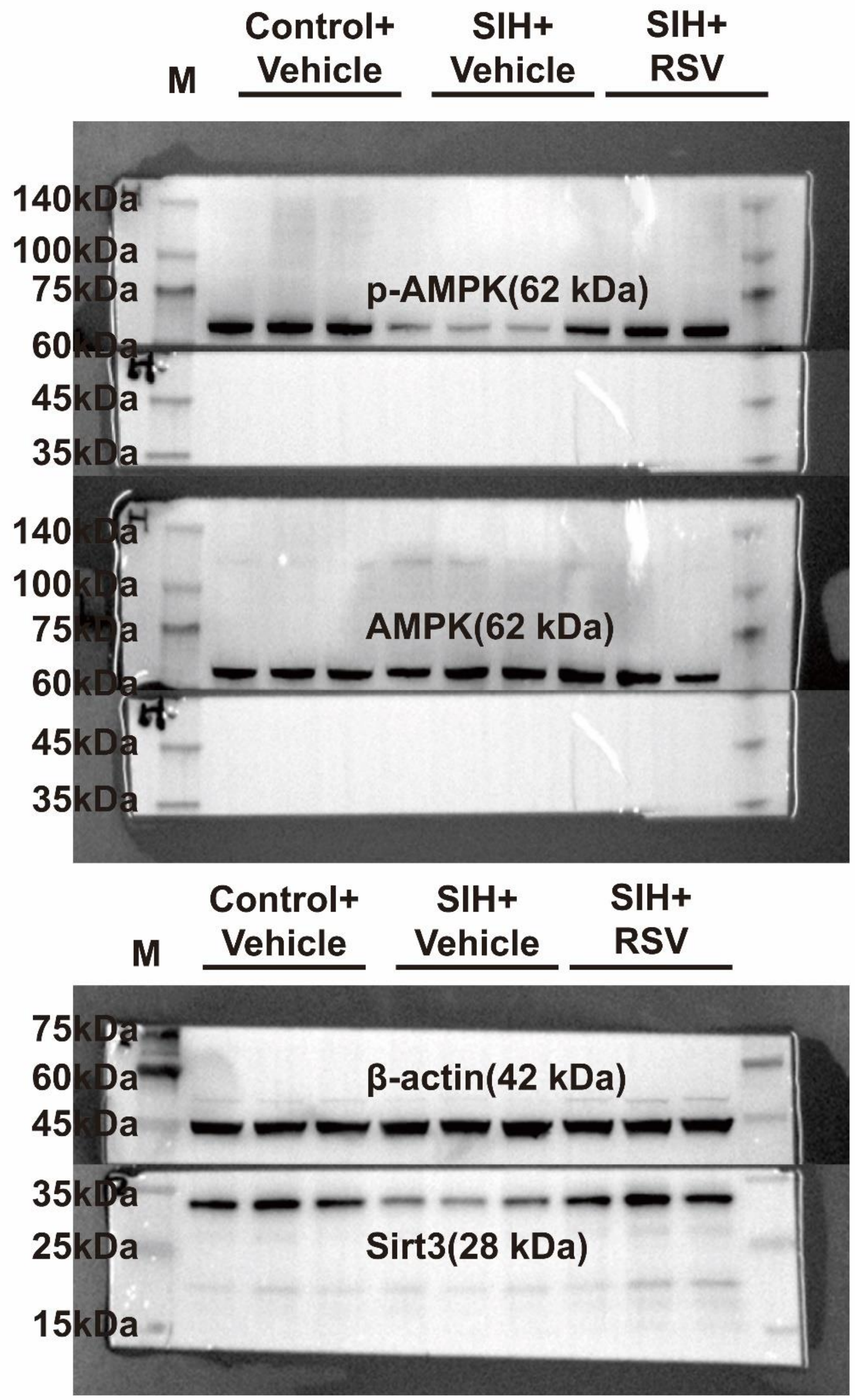
M
Control+ Vehicle
SIH+ Vehicle
SIH+ RSV
140kDa
100kDa
75kDa
p-AMPK(62 kDa)
60kDa
45kDa
35kDa
140kDa
100kDa
75kDa
AMPK(62 kDa)
60kDa
45kDa
35kDa
M
Control+ Vehicle
SIH+ Vehicle
SIH+ RSV
75kDa
60kDa
β-actin(42 kDa)
45kDa
35kDa
Sirt3(28 kDa)
25kDa
15kDa

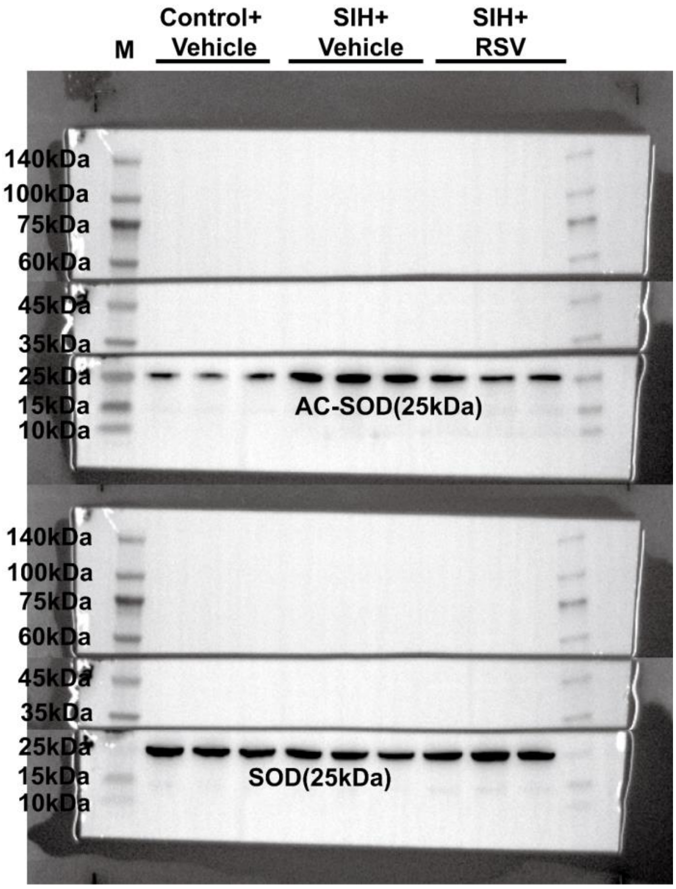

Full unedited blot for Figure S3. M, marker; SIH, stress-induced hypertension; RSV, resveratrol.

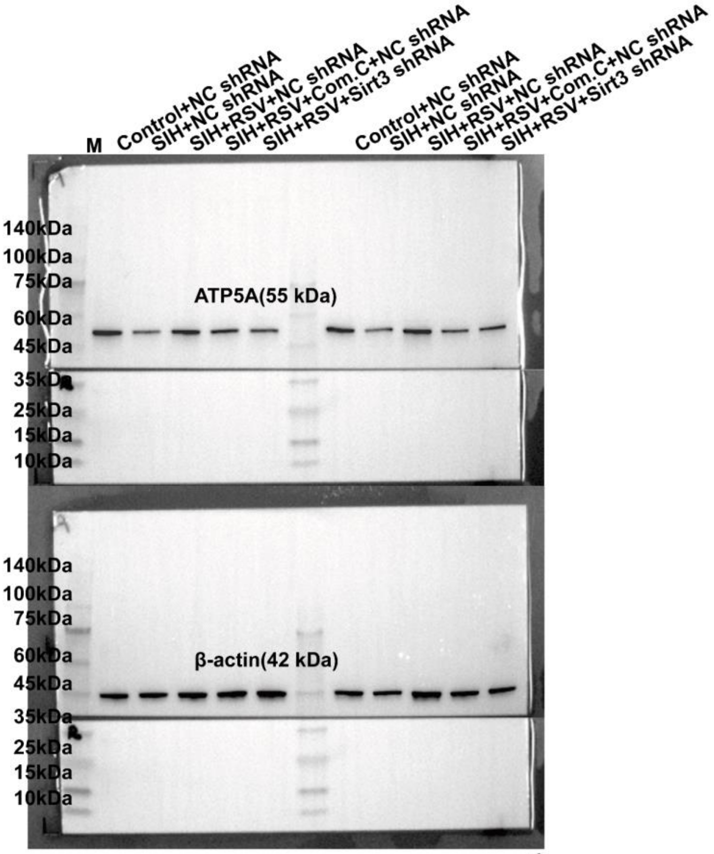
M
Control+NC shRNA
SIH+NC shRNA
SIH+RSV+NC shRNA
SIH+RSV+Com.C+NC shRNA
SIH+RSV+Sirt3 shRNA
Control+NC shRNA
SIH+NC shRNA
SIH+RSV+NC shRNA
SIH+RSV+Com.C+NC shRNA
SIH+RSV+Sirt3 shRNA
140kDa
100kDa
75kDa
60kDa
45kDa
35kDa
25kDa
15kDa
10kDa
ATP5A(55 kDa)
140kDa
100kDa
75kDa
60kDa
45kDa
35kDa
25kDa
15kDa
10kDa
β-actin(42 kDa)

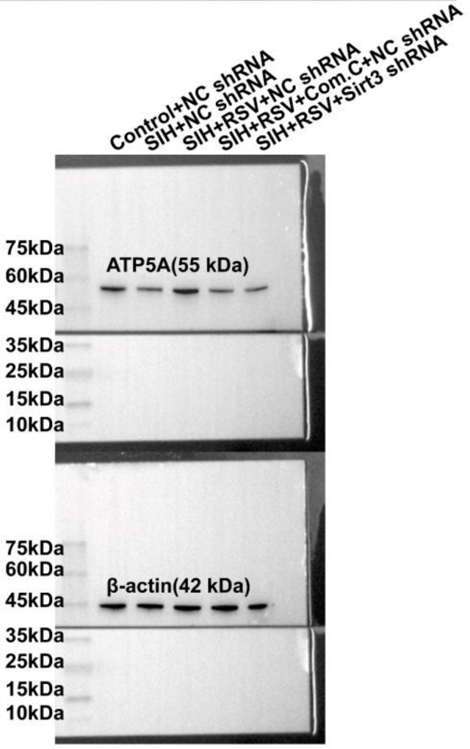
Control+NC shRNA
SIH+NC shRNA
SIH+RSV+NC shRNA
SIH+RSV+Com.C+NC shRNA
SIH+RSV+Sirt3 shRNA
75kDa
60kDa
45kDa
35kDa
25kDa
15kDa
10kDa
ATP5A(55 kDa)
75kDa
60kDa
45kDa
35kDa
25kDa
15kDa
10kDa
β-actin(42 kDa)

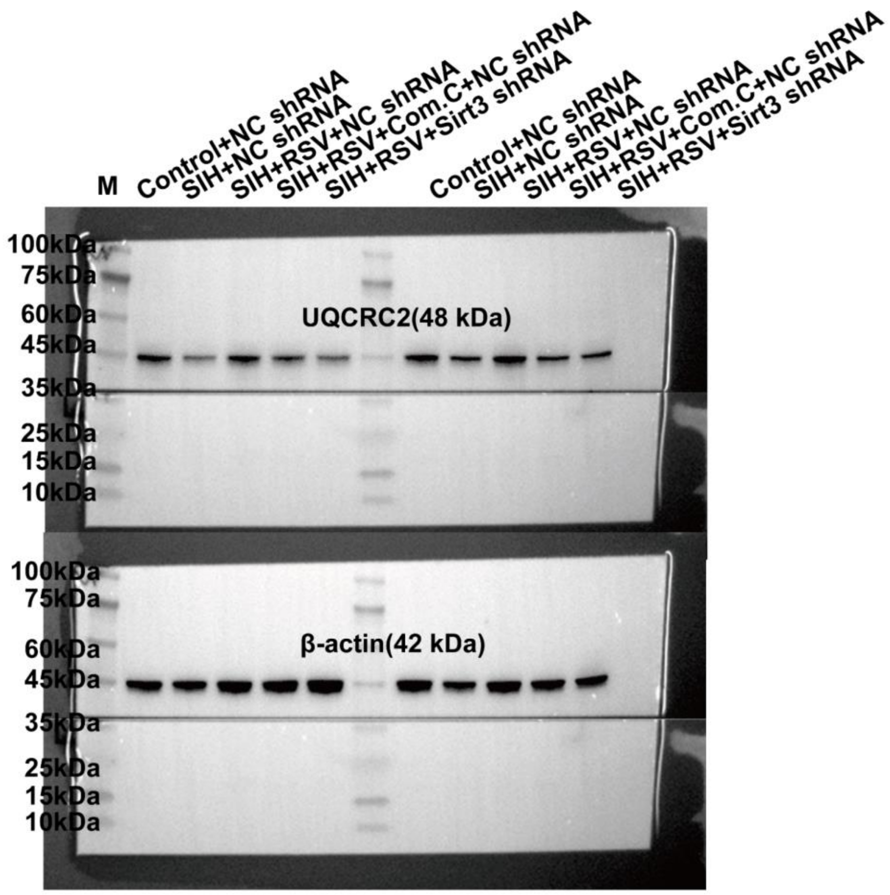
M
Control+NC shRNA
SIH+NC shRNA
SIH+RSV+NC shRNA
SIH+RSV+Com.C+NC shRNA
SIH+RSV+Sirt3 shRNA
Control+NC shRNA
SIH+NC shRNA
SIH+RSV+NC shRNA
SIH+RSV+Com.C+NC shRNA
SIH+RSV+Sirt3 shRNA
100kDa
75kDa
60kDa
45kDa
35kDa
25kDa
15kDa
10kDa
UQCRC2(48 kDa)
100kDa
75kDa
60kDa
45kDa
35kDa
25kDa
15kDa
10kDa
β-actin(42 kDa)

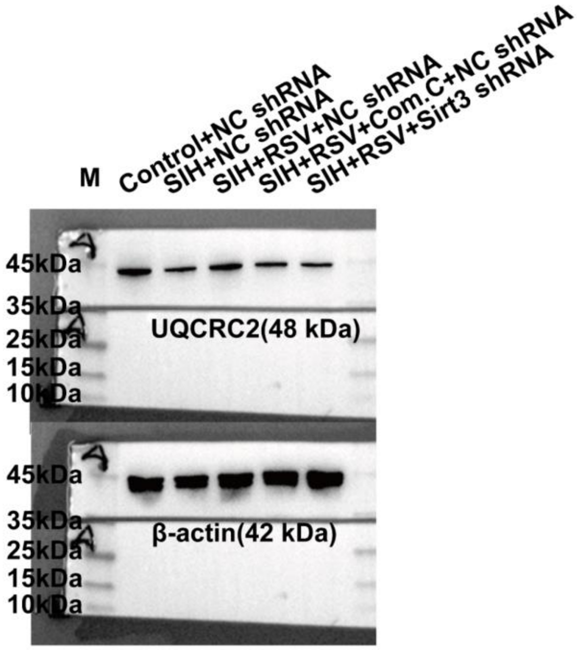
M
Control+NC shRNA
SIH+NC shRNA
SIH+RSV+NC shRNA
SIH+RSV+Com.C+NC shRNA
SIH+RSV+Sirt3 shRNA
45kDa
35kDa
25kDa
15kDa
10kDa
UQCRC2(48 kDa)
45kDa
35kDa
25kDa
15kDa
10kDa
β-actin(42 kDa)

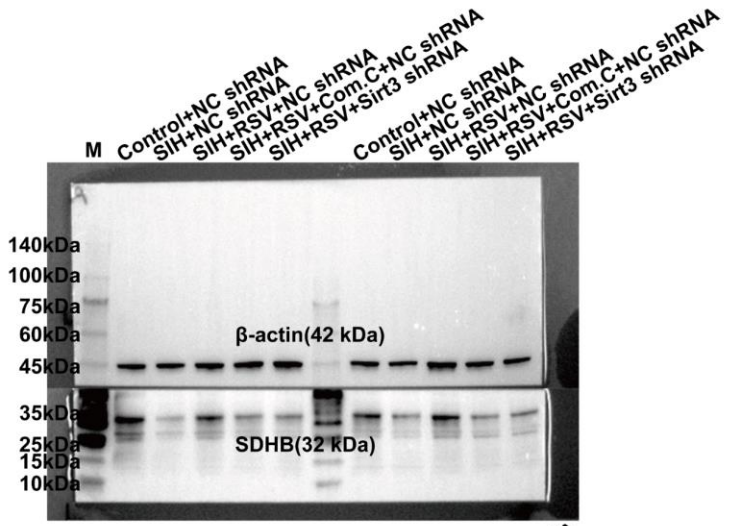

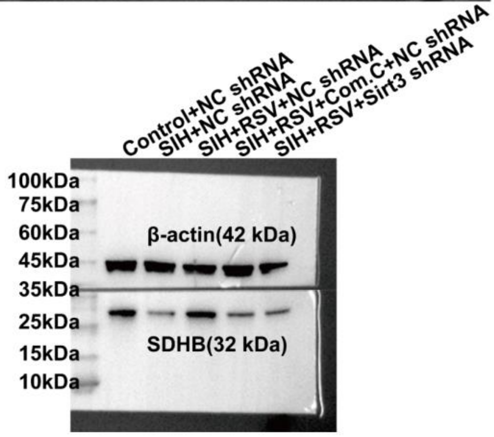

Full unedited blot for Figure S4. M, marker; SIH, stress-induced hypertension; RSV, resveratrol; Com.C, compound C; NC, negative control.

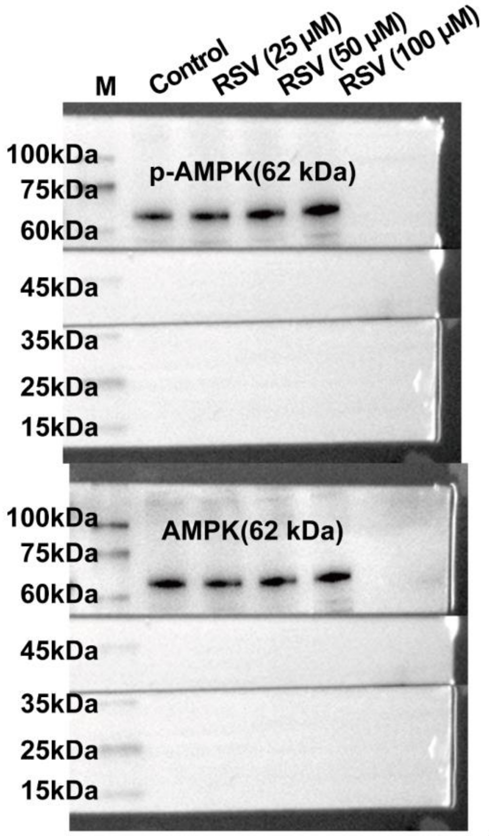
M
Control
RSV (25 μM)
RSV (50 μM)
RSV (100 μM)
p-AMPK(62 kDa)
100kDa
75kDa
60kDa
45kDa
35kDa
25kDa
15kDa
AMPK(62 kDa)
100kDa
75kDa
60kDa
45kDa
35kDa
25kDa
15kDa

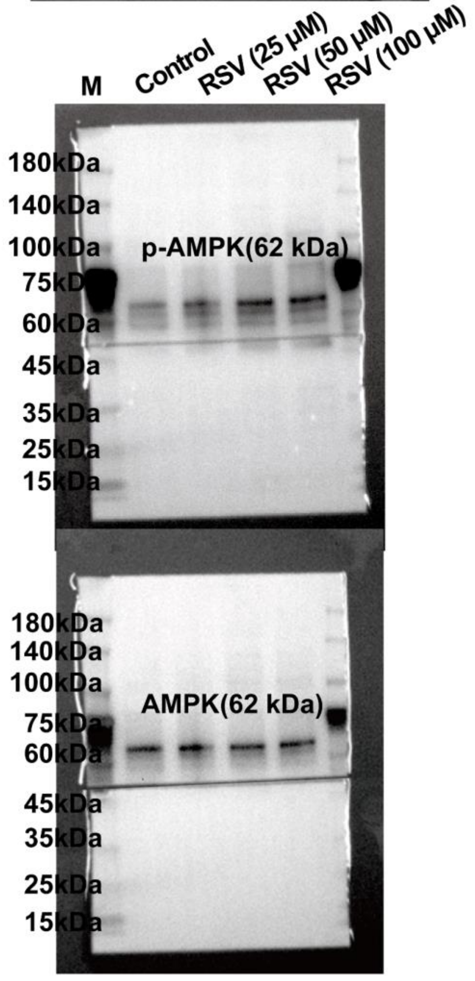
M
Control
RSV (25 μM)
RSV (50 μM)
RSV (100 μM)
180kDa
140kDa
100kDa
p-AMPK(62 kDa)
75kD
60kDa
45kDa
35kDa
25kDa
15kDa
180kDa
140kDa
100kDa
AMPK(62 kDa)
75kDa
60kDa
45kDa
35kDa
25kDa
15kDa

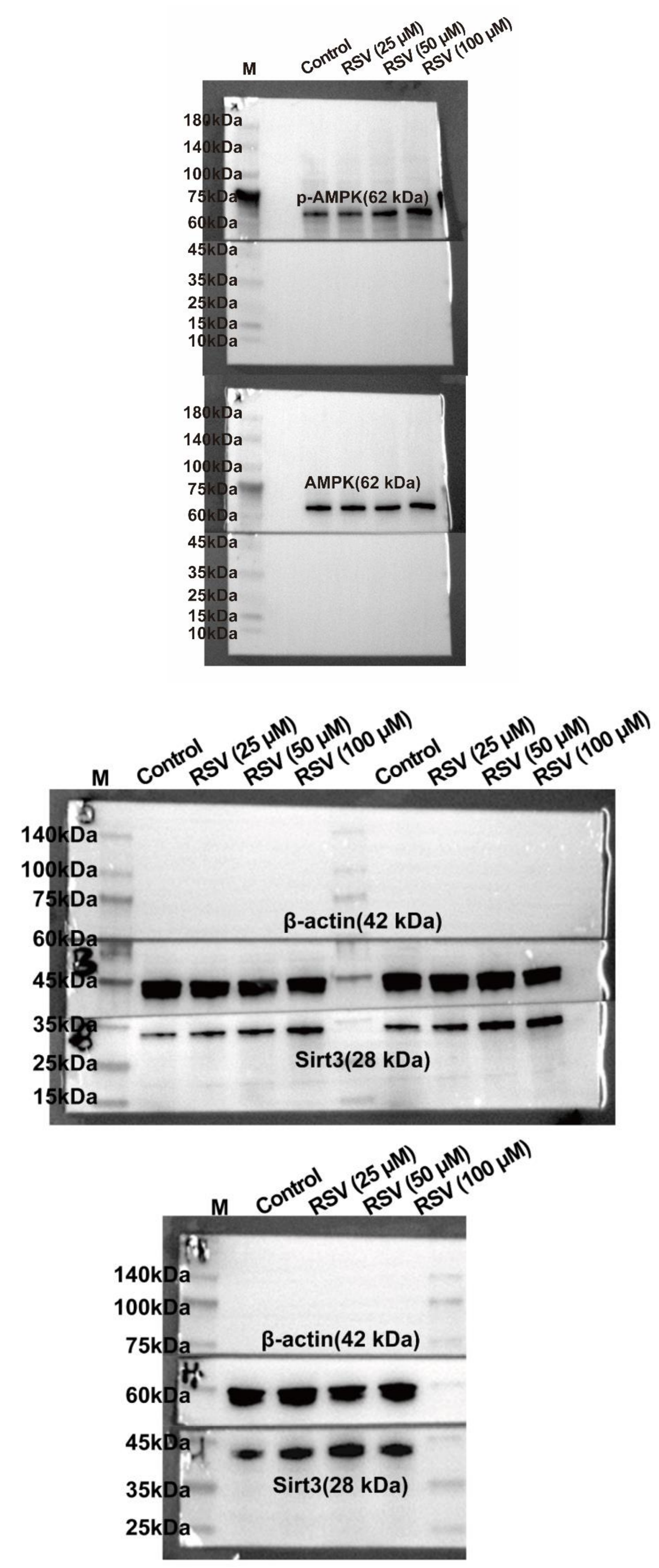

Full unedited blot for Figure S6A. M, marker; RSV, resveratrol.

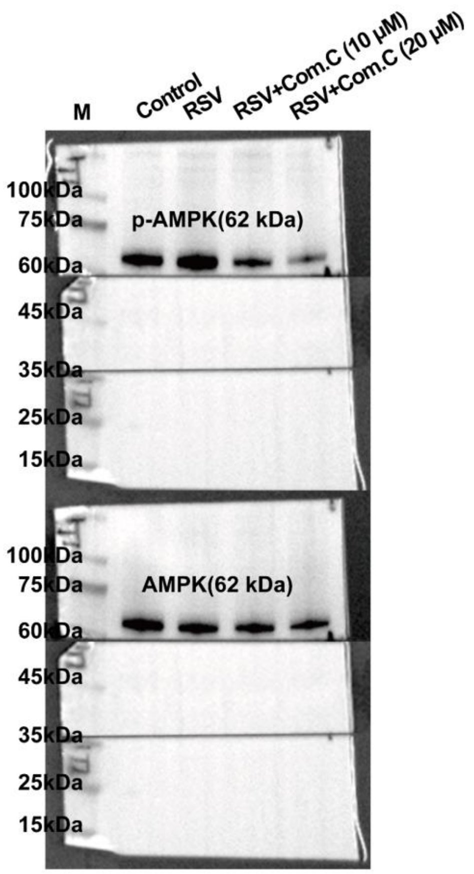
M
Control
RSV
RSV+Com.C (10 μM)
RSV+Com.C (20 μM)
100kDa
75kDa
p-AMPK(62 kDa)
60kDa
45kDa
35kDa
25kDa
15kDa
100kDa
75kDa
AMPK(62 kDa)
60kDa
45kDa
35kDa
25kDa
15kDa

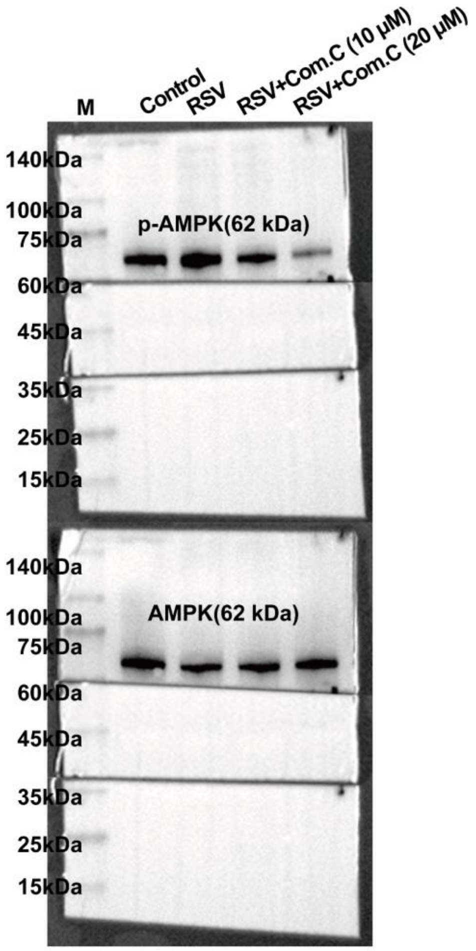
M
Control
RSV
RSV+Com.C (10 μM)
RSV+Com.C (20 μM)
140kDa
100kDa
p-AMPK(62 kDa)
75kDa
60kDa
45kDa
35kDa
25kDa
15kDa
140kDa
100kDa
AMPK(62 kDa)
75kDa
60kDa
45kDa
35kDa
25kDa
15kDa

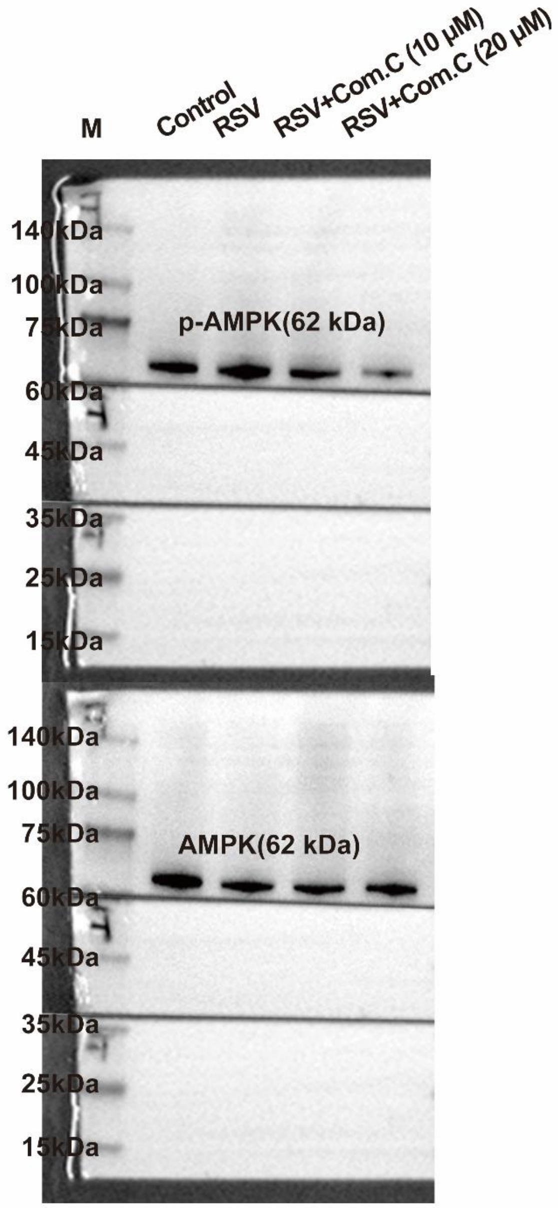
M
Control
RSV
RSV+Com.C (10 μM)
RSV+Com.C (20 μM)
140kDa
100kDa
75kDa
p-AMPK(62 kDa)
60kDa
45kDa
35kDa
25kDa
15kDa
140kDa
100kDa
75kDa
AMPK(62 kDa)
60kDa
45kDa
35kDa
25kDa
15kDa

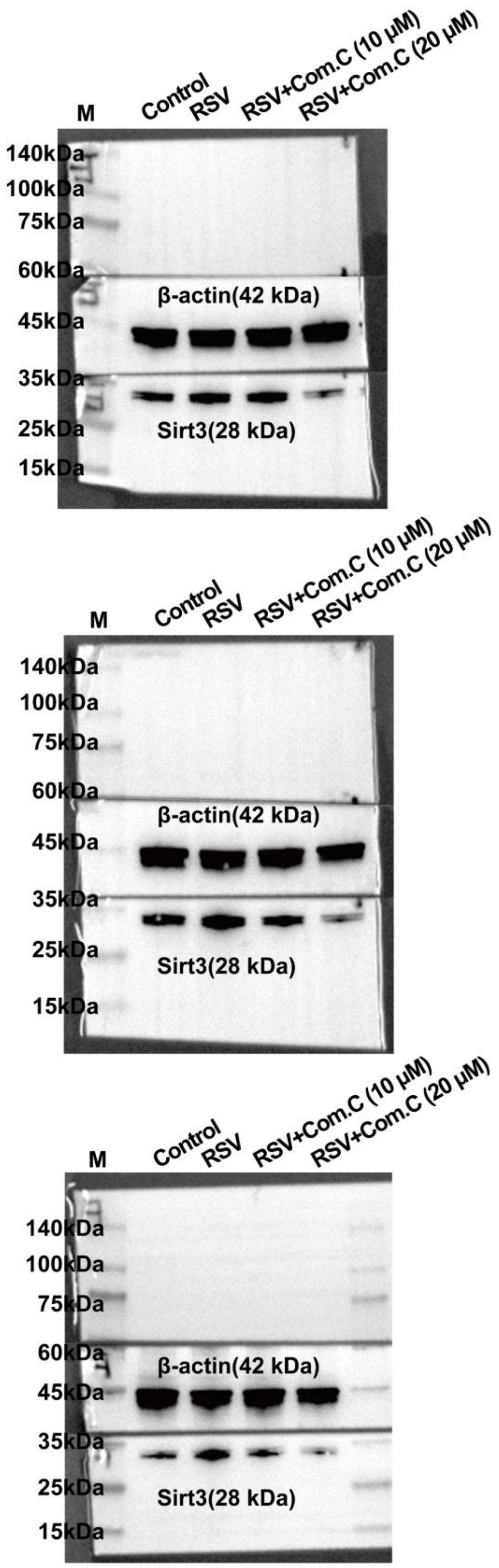

Full unedited blot for Figure S6B. M, marker; RSV, resveratrol; Com.C, compound C.

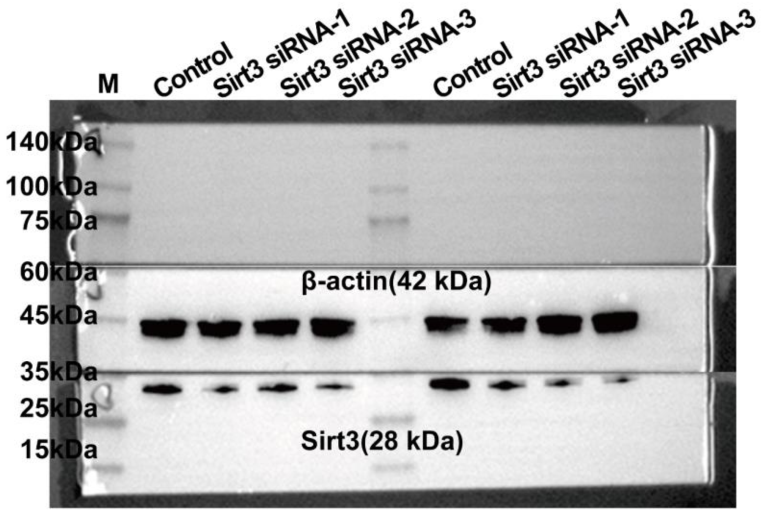

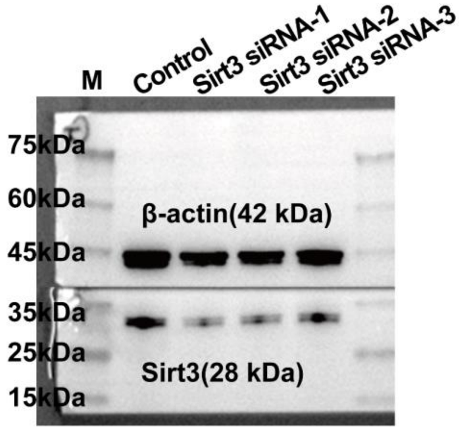

Full unedited blot for Figure S8. M, marker.
